# Trilobatin, a Naturally Occurring Food Additive, Ameliorates Exhaustive Exercise-Induced Fatigue in Mice: Involvement of Nrf2/ARE/Ferroptosis Signaling Pathway

**DOI:** 10.3389/fphar.2022.913367

**Published:** 2022-06-24

**Authors:** Ran Xiao, Yu Wei, Yueping Zhang, Fan Xu, Congjian Ma, Qihai Gong, Jianmei Gao, Yingshu Xu

**Affiliations:** ^1^ School of Pharmacy, Zunyi Medical University, Zunyi, China; ^2^ Key Laboratory of Basic Pharmacology of Ministry of Education and Joint International Research Laboratory of Ethnomedicine of Ministry of Education, Zunyi Medical University, Zunyi, China; ^3^ Department of Neurology, The Affiliated Hospital of Zunyi Medical University, Zunyi, China; ^4^ Spemann Graduate School of Biology and Medicine (SGBM), Albert-Ludwigs-University Freiburg, Freiburg, Germany

**Keywords:** exhaustive exercise-induced fatigue, trilobatin, oxidative stress, nuclear factor erythroid 2-related factor 2, ferroptosis

## Abstract

Nrf2-mediated oxidative stress is a promising target of exhaustive exercise-induced fatigue (EEIF). Trilobatin (TLB) is a naturally occurring food additive with antioxidant effect and Nrf2 activation potency. The present study aimed to investigate the effect of TLB on EEIF and elucidate its underlying mechanism. Our results showed that TLB exerted potent anti-EEIF effect, as reflected by the rope climbing test and exhaustive swimming test. Moreover, TLB also effectively reduced the levels of lactate, creatine kinase, and blood urea nitrogen, and increased liver glycogen and skeletal muscle glycogen in mice after EEIF insult. Additionally, TLB also balanced the redox status as evidenced by decreasing the generation of reactive oxygen species and improving the antioxidant enzyme activities including superoxide dismutase, catalase, and glutathione peroxidase, as well as the level of glutathione both in the tissue of muscle and myocardium. Furthermore, TLB promoted nuclear factor erythroid 2-related factor 2 (Nrf2) from the cytoplasm to the nucleus, and upregulated its downstream antioxidant response element (ARE) including quinone oxidoreductase-1 and heme oxygenase-1. Intriguingly, TLB also upregulated the GPx4 protein expression and reduced iron overload in mice after EEIF insult. Encouragingly, the beneficial effect of TLB on EEIF-induced oxidative stress and ferroptosis were substantially abolished in Nrf2-deficient mice. In conclusion, our findings demonstrate, for the first time, that TLB alleviates EEIF-induced oxidative stress through mediating Nrf2/ARE/ferroptosis axis.

## 1 Introduction

Exhaustive exercise-induced fatigue (EEIF) is generally termed as the incapacity of muscles to produce strength due to severe and/or extended exercise (Yang et al., 2020). EEIF not only lowers work efficiency but also evokes a series of fatigue-associated clinical features such as endocrine disorders, compromised immunity, and even organic illness (Abbasi et al., 2014; Dalsgaard et al., 2004; da Rocha et al., 2018). The exact mechanisms involved in EEIF are not completely revealed, but it is commonly recognized that the dominating causes of EEIF include oxidative stress, pro-inflammatory factors, depletion of energy, and accumulation of metabolites (Knoop et al., 2021). Among them, oxidative stress, defined as an unbalance between oxidants and antioxidants, plays a vital role in the EEIF. Regular physical exercise facilitates health benefits, while EEIF elicits a rapid increase in the generation of reactive oxygen species (ROS) that leads to demolition of redox signaling or molecular injury. Thus, delayed EEIF is always reflected by prolonged physical exercise endurance and reduced oxidative stress (Ma et al., 2021). Emerging evidence reveals that a dominating mechanism of the cellular defensive system against oxidative stress is the activation of the nuclear factor erythroid 2-related factor 2 (Nrf2)/antioxidant response element (ARE) signaling pathway (Zhang et al., 2021a). In physiological conditions, Nrf2 is bound with Kelch-like ECH-associated protein-1 (Keap1), which is an inhibitory protein settled in the cytoplasm, suppressing nuclear translocation of Nrf2. Upon oxidative stress stimuli, Nrf2 is dissociated from Keap1, translocates from cytoplasm into the nucleus, and activates its downstream ARE that propels antioxidant enzymes. Most strikingly, recent evidence demonstrates that Nrf2 harmonizes the cellular antioxidative defense in the regulation of ferroptosis, which is termed as a newly discovered form of programmed cell death that is induced by the iron-dependent lipid peroxidation and oxidative stress (Chen et al., 2022). However, whether Nrf2/ARE/ferroptosis is involved in the EEIF remains unclear.

Regrettably, until now, effective agents against EEIF are still unavailable in the clinic due to its severe or unexpected adverse drug reactions such as psychiatric disorder, physical dependence, and addiction (Smith, 2002; Kerksick et al., 2017; Nygård et al., 2018). Hence, safer and more effective agents or tactics need to be urgently developed in the clinic. Fortunately, food raw ingredients defined as “generally recognized as safe” (GRAS) are a reliable source of secure chemical substances for developing new drugs for EEIF. TLB is a sweetener from *Lithocarpus polystachyus* Rehd and acts as a food additive with its splendid safety profile (Gao et al., 2022). Recent evidence reveals that TLB exhibits multiple pharmacological properties including anti-ischemic stroke effect, anti-HIV-1 effect, anti-aging, and anti-Alzheimer’s disease effect (Yin et al., 2018; Chen et al., 2020a; Li et al., 2021; Gao et al., 2022). Interestingly, our previous studies have revealed that TLB protects against hydrogen peroxide-induced PC12 cell injury or cerebral ischemia reperfusion injury *via* mediating the Nrf2/ARE signaling pathway (Gao et al., 2018; Gao et al., 2020a). Thus, we come up with a hypothesis that TLB can ameliorate EEIF-induced oxidative injury *via* mediating Nrf2/ARE/ferroptosis. To test our hypothesis, this study was aimed to probe the effect of TLB on EEIF in mice and elucidate its possible mechanism.

## 2 Materials and Methods

### 2.1 Chemicals and Reagents

TLB (purity 98% by HPLC) was purchased from Chengdu Push Biotechnology Medical Technology Corporation (Chengdu, China). Taurine (purity 99% by HPLC) was purchased from Qianjiang Yongan Pharmaceutical Co., Ltd. (Hubei, China). Blood urea nitrogen (BUN), reactive oxygen species (ROS), malondialdehyde (MDA), creatine kinase (CK), lactic acid (LA), liver glycogen (LG), muscle glycogen (MG), superoxide dismutase (SOD), catalase (CAT), glutathione peroxidase (GPX), glutathione (GSH), glutathione peroxidase 4 (GPx4), and iron porphyrin assay kits were obtained from Shanghai Renjie Bioengineering Institute (Shanghai, China).

### 2.2 Animals

Male wild-type (WT) C57BL/6 mice (6–8 week old, 23–25 g) were purchased from Hunan SJA Laboratory Animal Co., Ltd. (Certificate No. SYXK 2018–0006, Hunan, China). Male Nrf2-deficient (Nrf2^−/−^) mice and littermate WT mice (6–8 week old, 23–25 g) were purchased from Cyagen Biosciences Inc. (Certificate No. KOCMP-21018-Nfe2l2, Suzhou, China). WT mice and Nrf2^−/−^ mice were allowed to acclimatize to the experimental conditions (temperature of 25 ± 1°C, relative humidity of 55% ± 5%, and a 12-h light–dark cycle) for a week. Laboratory chow and tap water were provided *ad libitum*. All animal experimental protocols in the present study were operated according to the Guide for the Care and Use of Laboratory Animals published by the United States National Institutes of Health (National Institutes of Health Publication 85–23, revised 1996), and were approved by the Experimental Animal Ethics Committee of the Zunyi Medical University (Guizhou, China).

### 2.3 EEIF Protocol and Drug Treatments

The EEIF mouse model was established using a weight-loaded forced swimming test (WST) according to previous studies (Ma et al., 2022). In brief, mice were placed into a plastic water tank (80 cm long, 80 cm wide, 60 cm high, 30 cm deep, and water temperature 25 ± 1°C), and all mice swam for 60 min (once a day). After 28 days, mice swam loaded with a lead (weight of 5% of their body weight) in the tail, and endurance time was documented when mice were completely exhausted and could not return to the water surface within 3 s.

Protocol 1: Mice were randomly divided into five groups: vehicle group, vehicle + TLB (2.5 mg/kg) group, vehicle + TLB (5 mg/kg) group, vehicle + TLB (10 mg/kg) group, and vehicle + taurine-positive (1 g/kg) group.

Protocol 2: WT and Nrf2^−/−^ mice were randomly divided into four groups: WT-vehicle group, WT-vehicle + TLB (10 mg/kg) group, Nrf2^−/−^-vehicle group, and Nrf2^−/−^-vehicle + TLB (10 mg/kg) group. The drug treatment groups were treated with TLB or taurine (1 g/kg) by gavage for 28 days (twice a day), and the vehicle groups were given volume-matched saline. Taurine was used as a positive drug as it is not only a natural food additive but also utilized for anti-EEIF in the clinic.

### 2.4 Rope Climbing Test

After the weight-loaded forced swimming test, the resting endurance of mice was measured by performing the rope-climbing test as reported in a previous study. Before the formal test, each mouse underwent three days of adaptive training. In brief, mice are placed on the center of a rope with a diameter of 5 mm and a length of 35 cm with a rough surface. The time from the start of climbing to the time of slipping into the water is recorded as the rope-climbing time. Tissue collection was conducted after the rope-climbing test.

### 2.5 Enzyme-Linked Immunosorbent Assay

The biochemical indexes and oxidative stress indicators were determined using the ELISA assay. In brief, supernatant of the serum, gastrocnemius muscle in the calf of the mouse, heart, or liver tissues were collected and centrifuged at 3000 × g for 20 min. The levels of LA (RJ17548), CK (RJ17220), BUN (RJ17469), LG (RJ17115), MG (RJ24589), ROS (RJ17213), MDA (RJ16984), SOD (RJ17004), CAT (RJ17177), GPx (RJ17154), GSH (RJ17183), GPx4 (RJ17992), and iron porphyrin (KD-23021) were detected according to the instructions of appropriate ELISA kits. The absorbance value was detected at a wavelength of 450 nm on a microplate reader and the concentration was calculated according to the standard curve.

### 2.6 Hematoxylin and Eosin Staining

Histological examination was determined using HE staining. In brief, slices (5 μm) of gastrocnemius muscle tissues were embedded in paraffin and stained with HE for 15 min at 60°C. Thereafter, the histological change was observed under the microscope (BX 43 Olympus, Tokyo, Japan).

### 2.7 Western Blot

Western blot was performed as described in our previous study (Gao et al., 2020b; Xu et al., 2020). Protein samples were extracted from gastrocnemius muscle tissues in the calf of the mouse using RIPA buffer supplemented with the protease inhibitor PMSF. After incubation and centrifugation (12,000 ×g, 15 min, 4°C), the supernatant was collected and total protein levels were quantified using a BCA Assay Kit. Equal amounts of total protein (30 μg) were separated on a 10% SDS-polyacrylamide gel. Following that, anti-Nrf2 (1:1000, ab92946), anti-heme oxygenase 1 (HO-1, 1:1000, ab68477), anti-NADPH quinone oxidoreductase 1 (NQO-1, 1:1000, ab137550), anti-Keap-1(1:1000, ab119403), anti-β-actin (1: 5000, ab8227), anti-GAPDH (1: 5000, ab8245), anti-GPx4(1:1000, ab125066), and anti-PCNA (1: 5000, ab29) were incubated at 4°C for 24 h. Next, the proteins were cultured with species-specific HRP-conjugated secondary antibodies (1:5000) at room temperature for 1 h. Then, immunoreactive bands were visualized with ECL detection reagents and quantified on a ChemiDoc MP imaging system (Bio-Rad Laboratories, Inc., Hercules, CA, United States).

### 2.8 Statistical Analysis

All values are presented as the mean ± standard error of the mean (SEM) and were analyzed using SPSS 18.0 (SPSS, Inc., Chicago, United States). Statistical comparisons were made using two-way analysis of variance (ANOVA) with Bonferroni’s *post hoc* test or one-way ANOVA with Tukey’s *post hoc* test or Student’s unpaired *t*-test where appropriate. *p *< 0.05 was considered a statistically significant difference.

## 3 Results

### 3.1 Effect of TLB on EEIF in Mice

**FIGURE 1:**
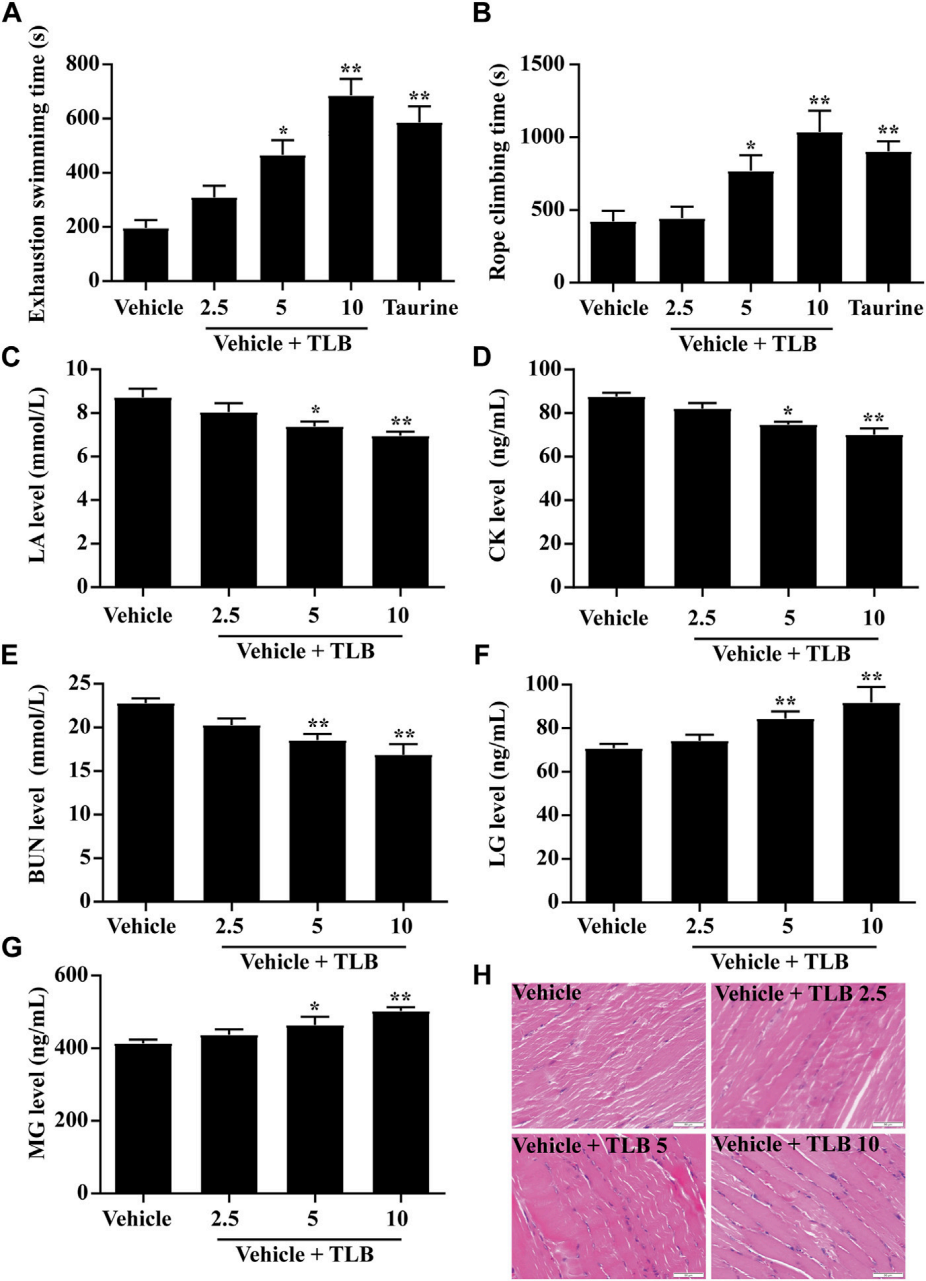
Effect of TLB on EEIF in mice. **(A)** Exhaustive swimming time (*n* = 10), **(B)** roping fatigue time (*n* = 10), **(C)** LA level (n = 6), **(D)** CK level (*n* = 6), **(E)** BUN level (*n* = 6), **(F)** LG level (*n* = 6), **(G)** MG level (*n* = 6), and **(H)** histological change of skeletal muscle tissue was determined using HE staining. The data were presented as the mean ± SEM. ^*^ *p* < 0.05 and ^**^ *p* < 0.01 *vs* vehicle group (magnification ×400 and scale bar = 50 μm).

The effect of TLB on EEIF was evaluated by WST and the rope-climbing test. The results showed that TLB (5, 10 mg/kg) or taurine (a positive drug, 1 g/kg) significantly increased the exhaustive swimming time of mice compared with the vehicle group (*p* < 0.05) (Figure 1A). In addition, in keeping with the findings of WST, TLB (5 and 10 mg/kg) or taurine also markedly increased climbing rope dwell time compared with the vehicle group (*p* < 0.05) (Figure 1B). Moreover, TLB (5 and 10 mg/kg) evidently mitigated LA, CK, and BUN levels, and elevated LG and MG levels of mice compared with the vehicle group (*p* < 0.05) (Figures 1C–G). Meanwhile, obvious structural damage including high contraction, uneven thickness, and muscle fiber fracture of skeletal muscle was observed in mice of the vehicle group, while these changes were reversed by TLB at doses of 5 and 10 mg/kg (Figure 1H). These findings suggest that TLB exerts a potent anti-fatigue effect on EEIF in mice.

### 3.2 Effect of TLB on EEIF-Induced Oxidative Stress and Ferroptosis in Skeletal Muscle

**FIGURE 2:**
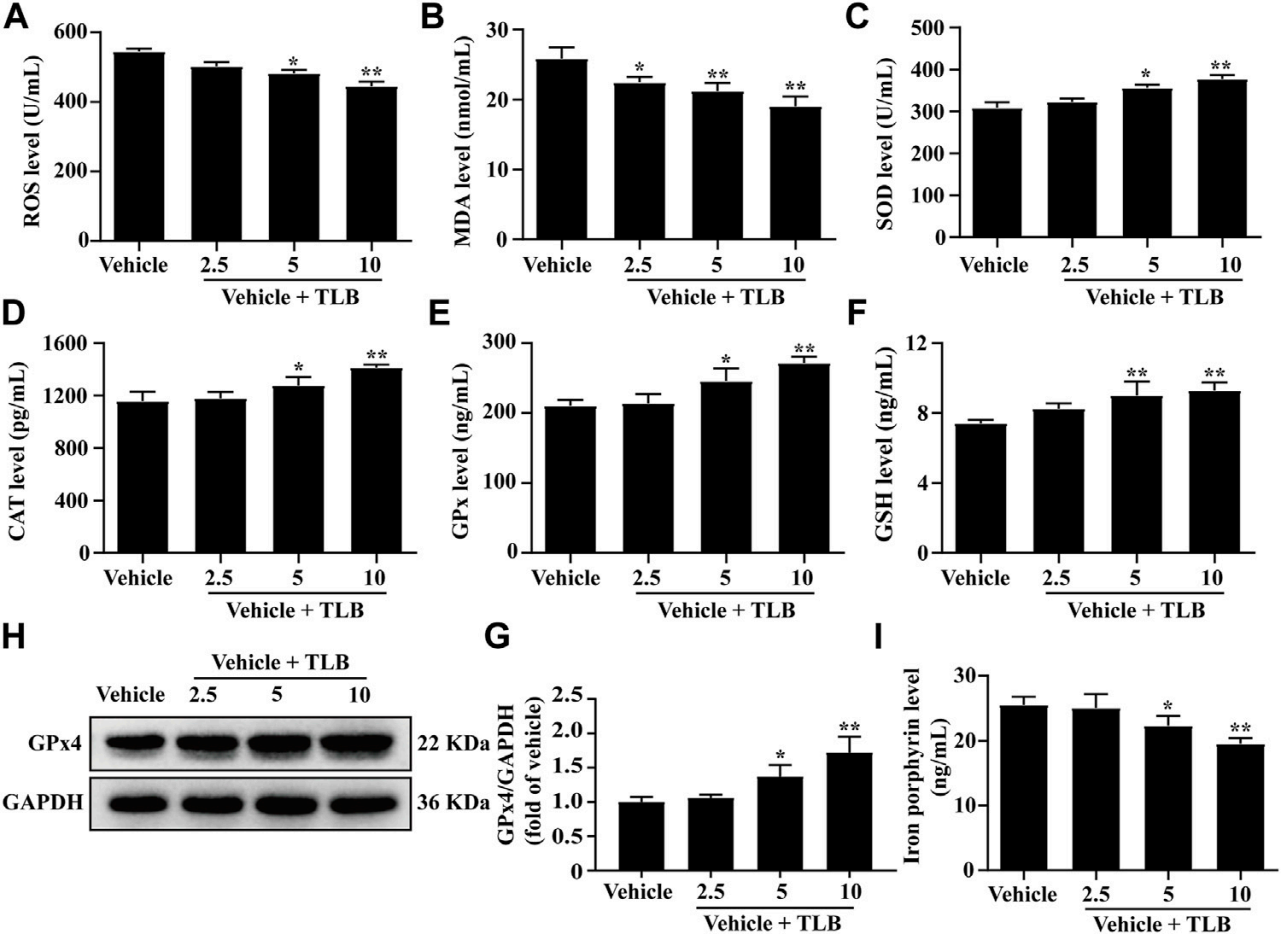
Effect of TLB on EEIF-induced oxidative stress and ferroptosis in mice. **(A)** ROS level (*n* = 6), **(B)** MDA level (*n* = 6), **(C)** CAT activity (*n* = 6), **(D)** SOD activity (*n* = 6), **(E)** GPx activity (*n* = 6), **(F)** GSH level (*n* = 6), **(H)** representative Western blots of GPx4, **(G)** quantitation of GPx4 (*n* = 5), and **(I)** iron porphyrin level (*n* = 6). The data were presented as the mean ± SEM. ^*^ *p* < 0.05 and ^**^ *p* < 0.01 *vs* vehicle group.

The effect of TLB on EEIF-induced oxidative stress and ferroptosis in skeletal muscle of mice was investigated by the ELISA assay. The results showed that TLB (5 and 10 mg/kg) apparently lowered the levels of ROS and MDA in mice compared with the vehicle group (*p* < 0.05) (Figures 2A,B). Moreover, TLB (5 and 10 mg/kg) also increased the activities of SOD, CAT, GPx, and GSH in mice compared with the vehicle group (*p* < 0.05) (Figures 2C–F). What is more, TLB (5 and 10 mg/kg) obviously increased the protein expression of GPx4 and decreased iron porphyrin levels in mice compared with the vehicle group (*p* < 0.05) (Figures 2G,H). These results indicate that TLB effectively inhibited EEIF-induced oxidative stress and ferroptosis in mice.

### 3.3 Effect of TLB on EEIF-Induced Oxidative Stress in Myocardium

**FIGURE 3:**
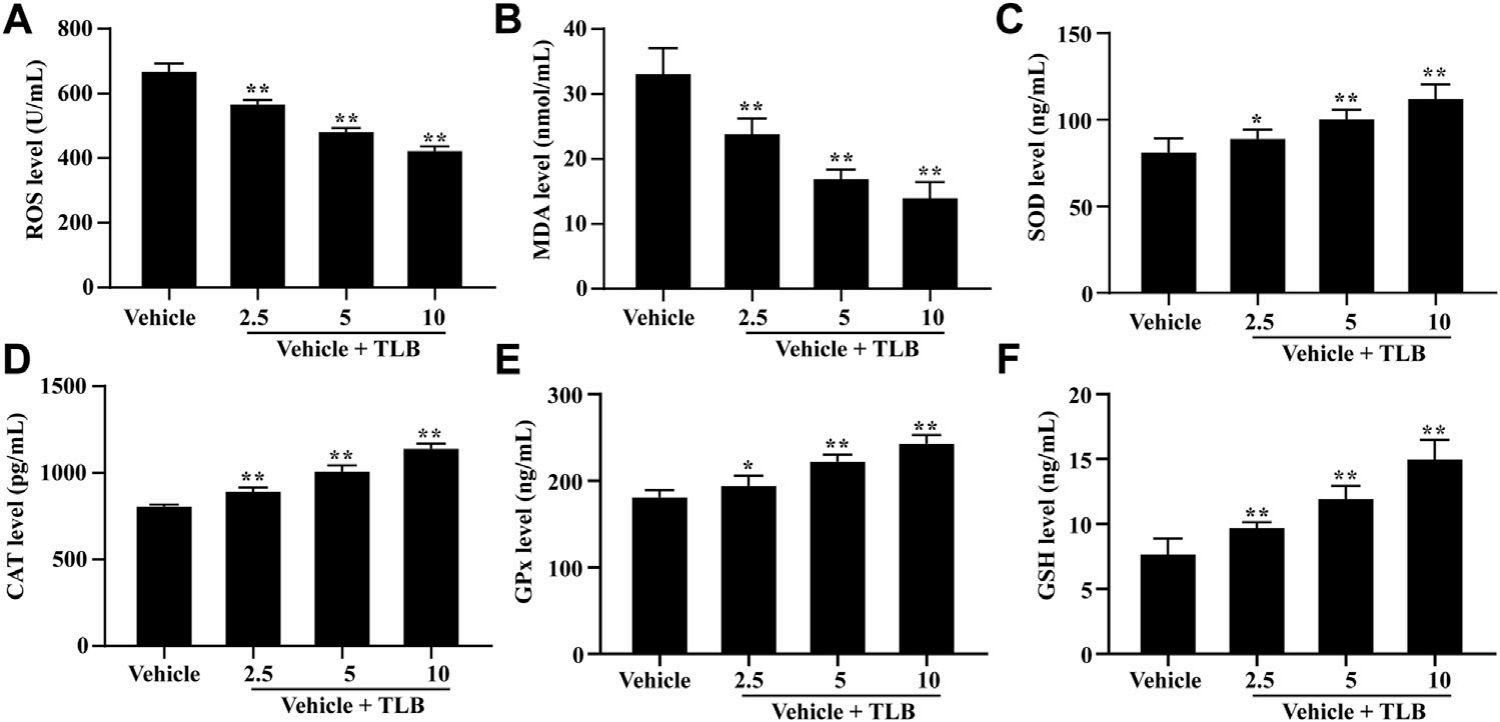
Effect of TLB on oxidative stress in heart of fatigue mice. **(A)** ROS level, **(B)** MDA level, **(C)** CAT activity, **(D)** SOD activity, **(E)** GPx activity, and **(F)** GSH level. The data were presented as the mean ± SEM (*n* = 10). ^*^ *p* < 0.05 and ^**^ *p* < 0.01 *vs* vehicle group.

To explore the effect of TLB on EEIF-induced oxidative stress in the heart, we also determined the oxidative stress in the myocardium of mice after EEIF using an ELISA assay. The results showed that TLB (2.5, 5, and 10 mg/kg) significantly reduced the levels of ROS and MDA in the myocardium of mice compared with the vehicle group (*p* < 0.05) (Figures 3A,B). What is more, TLB (2.5, 5, and 10 mg/kg) also enhanced the SOD, CAT, and GPx activities, as well as the GSH content in the myocardium of mice compared with the vehicle group (*p* < 0.05) (Figures 3C–F). These findings demonstrate that TLB also exerts protective effects in EEIF-induced heart damage in mice.

### 3.4 Effect of TLB on the Nrf2/ARE Signaling Pathway in EEIF Mice

**FIGURE 4:**
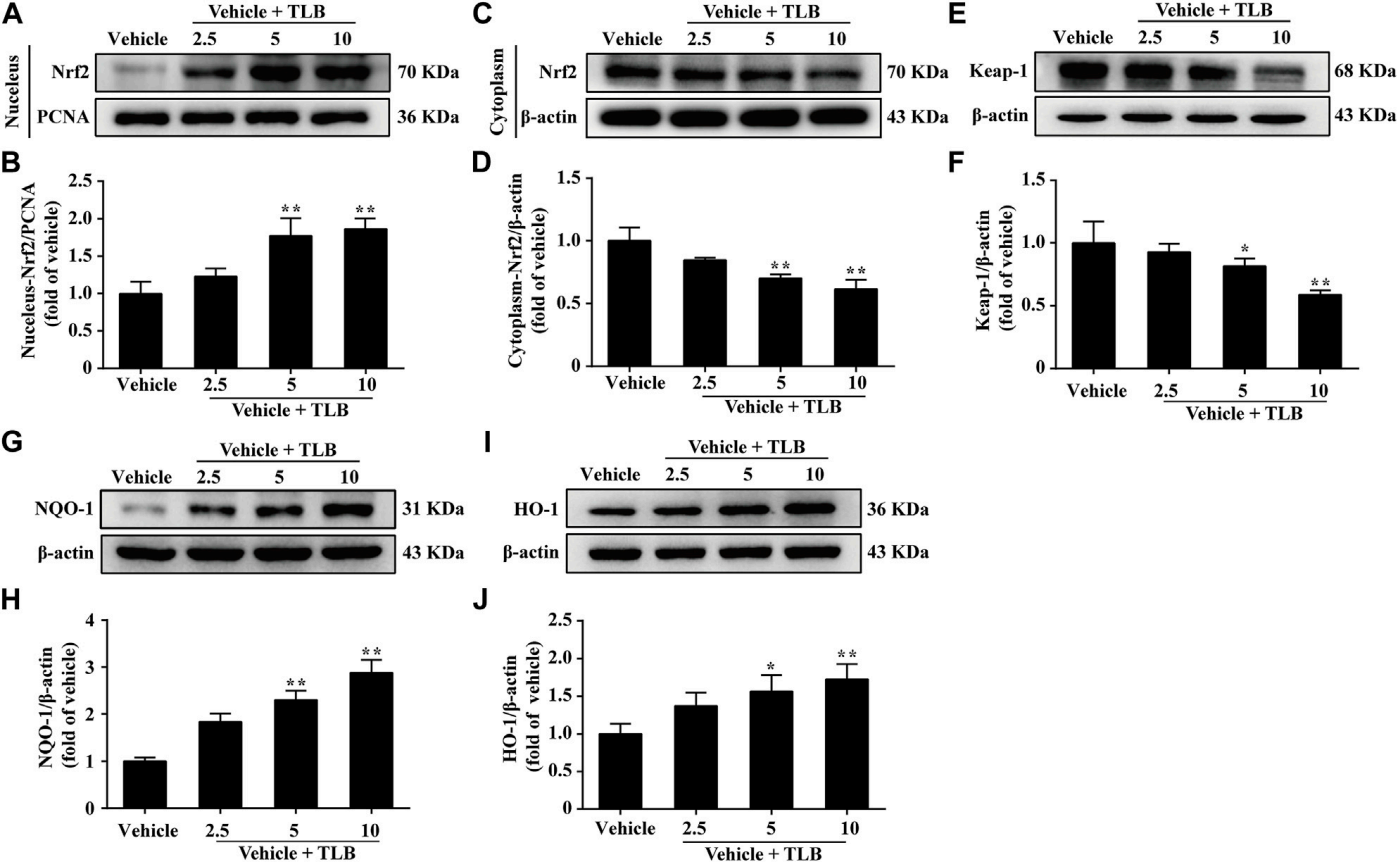
Effect of TLB on Nrf2/ARE signaling pathway on EEIF in mice. **(A)** Representative Western blots of nucleus-Nrf2. **(B)** Quantitation of nucleus-Nrf2. **(C)** Representative Western blots of cytoplasm-Nrf2. **(D)** Quantitation of cytoplasm-Nrf2. **(E)** Representative Western blots of Keap-1. **(F)** Quantitation of Keap-1. **(G)** Representative Western blots of NQO-1. **(H)** Quantitation of NQO-1. **(I)** Representative Western blots of HO-1. **(J)** Quantitation of HO-1. The data were presented as the mean ± SEM (*n* = 5). ^*^ *p* < 0.05 and ^**^ *p* < 0.01 *vs* vehicle group.

Furthermore, the effect of TLB on the Nrf2/ARE signaling pathway in EEIF mice was using the Western blot analysis. The results showed that TLB (5, 10 mg/kg) significantly increased the expression levels of the nuclear Nrf2 protein (*p* < 0.05) (Figures 4A,B) but decreased the cytoplasmic Nrf2 protein level and Keap1 protein expression (*p* < 0.05) (Figures 4C–F). Subsequently, TLB (5, 10 mg/kg) also remarkably up-regulated the expressions of its downstream HO-1 and NQO-1 proteins (*p* < 0.05) (Figures 4G–J). These results suggest that the protective effect of TLB on EEIF in mice, at least partly, through activation of the Nrf2/ARE signaling pathway.

### 3.5 Effect of TLB on EEIF in Nrf2^-/-^Mice

**FIGURE 5:**
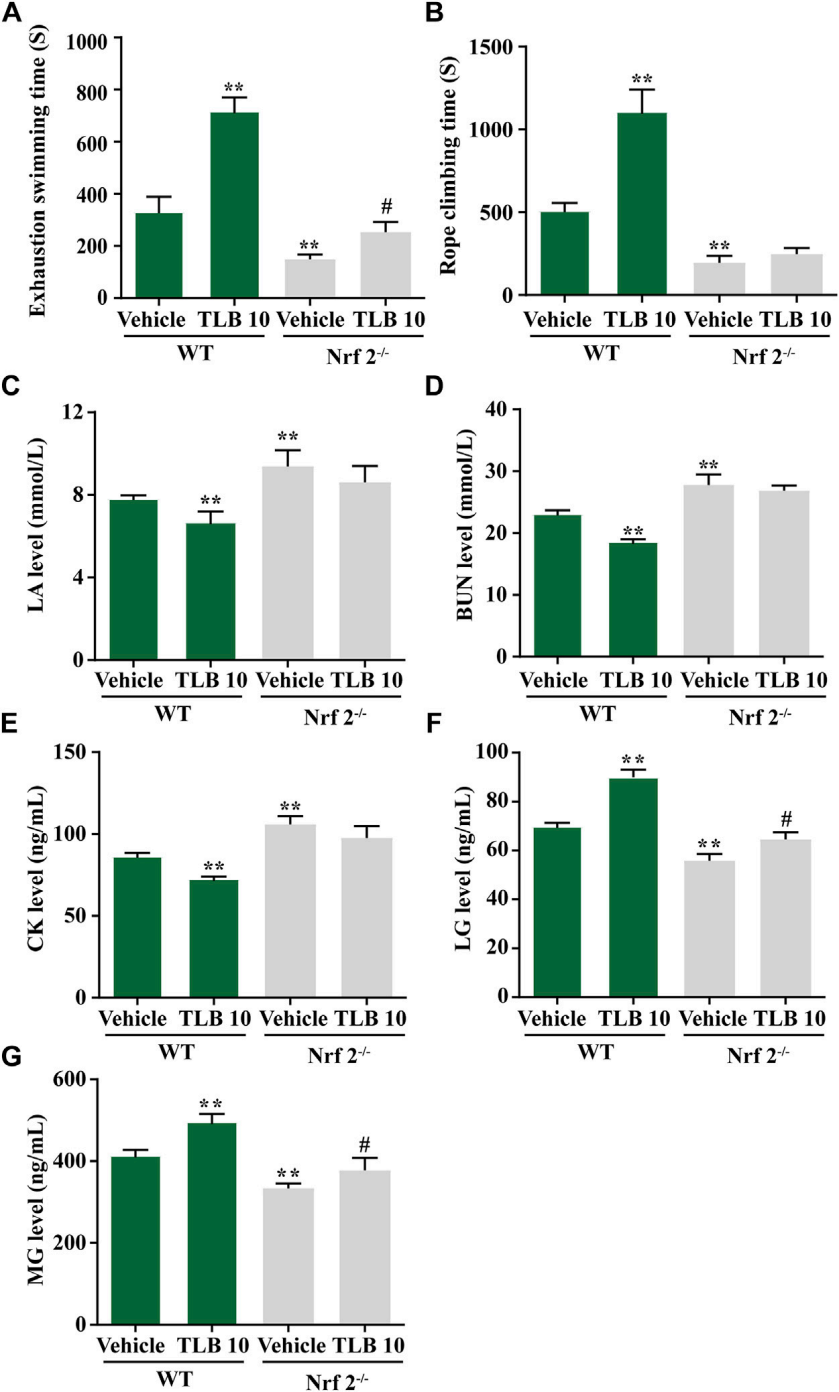
Effect of TLB on EEIF in Nrf2^-/-^mice. **(A)** Exhaustive swimming time, **(B)** roping fatigue time, **(C)** LA level, **(D)** CK level, **(E)** BUN level, **(F)** LG level, and **(G)** MG level. The data were presented as the mean ± SEM (*n* = 10). ^*^ *p* < 0.05 and ^**^ *p* < 0.01 *vs* vehicle group. ^#^ *p* < 0.05 vs Nrf2^−/−^ vehicle group.

Nrf2^−/−^ mice were utilized to confirm whether Nrf2 is a latent therapeutic target of TLB against EEIF. The results displayed that exhaustive swimming time and climbing rope dwell time of the Nrf2^-/-^-vehicle group were clearly reduced compared with the WT-vehicle group; however, promotive effects of TLB on exhaustive swimming time and climbing rope dwell time were substantially offset in Nrf2^−/−^ mice (*p* < 0.05) (Figures 5A,B). Also, consistent with the results of behavioral assessment outcome, LA, CK, and BUN levels of mice were distinctly augmented in the Nrf2^-/-^-vehicle group compared with the WT-vehicle group, whereas the inhibitory effects of TLB on LA, CK, and BUN were almost offset (*p* < 0.05) (Figures 5C–E). Additionally, LG and MG levels were overtly reduced in the Nrf2^-/-^-vehicle group compared with the WT-vehicle group, whereas the enhancement effects of TLB on LG and MG were largely abrogated in Nrf2^−/−^ mice (*p* < 0.05) (Figures 5F,G). These findings confirm that Nrf2 plays a vital role in the protective effect of TLB on EEIF.

### 3.6 Effect of TLB on EEIF-Induced Oxidative Stress and Ferroptosis in Skeletal Muscle of Nrf2^−/−^ Mice

**FIGURE 6:**
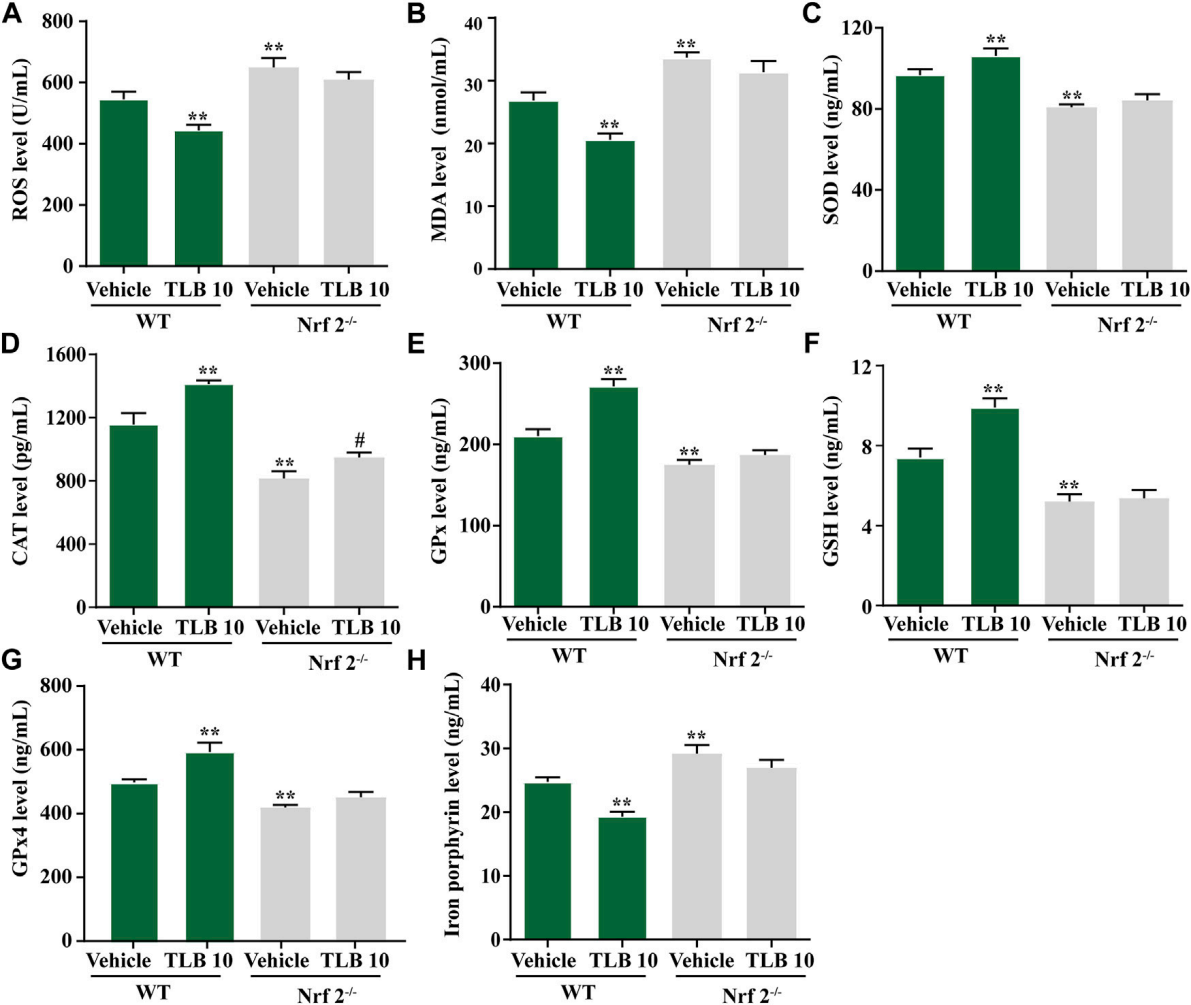
Effect of TLB on EEIF-induced oxidative stress and ferroptosis in Nrf2^−/−^ mice. **(A)** ROS level, **(B)** MDA level, **(C)** CAT activity, **(D)** SOD activity, **(E)** GPx activity, **(F)** GSH level, **(G)** iron porphyrin, and **(H)** iron porphyrin. The data were presented as the mean ± SEM (*n* = 10). ^*^ *p* < 0.05 and ^**^ *p* < 0.01 *vs* vehicle group. ^#^ *p* < 0.05 vs Nrf2^−/−^ vehicle group.

To further investigate the role of Nrf2 on the protective effects of TLB on EEIF-induced oxidative stress and ferroptosis in skeletal muscle, we tested indicators of oxidative stress and ferroptosis in Nrf2^−/−^ mice using the ELISA assay. The results exhibited that ROS and MDA levels in mice were obviously fortified in the Nrf2^-/-^-vehicle group compared with the WT-vehicle group, while the inhibitory effects of TLB on ROS and MDA were almost abolished in Nrf2^−/−^ mice (*p* < 0.05) (Figures 6A,B). Also, SOD, CAT, and GPx activities as well as the GSH content were markedly decreased in mice of the Nrf2^-/-^-vehicle group compared with the WT-vehicle group, whereas the promotive effects of TLB were substantially canceled in Nrf2^−/−^ mice (*p* < 0.05) (Figures 6C–F). Of note, GPx4 was reduced and the iron level was increased in mice of the Nrf2^-/-^-vehicle group compared with the WT-vehicle group, while the beneficial effect of TLB on the change of GPx4 and iron was dramatically reversed in Nrf2^−/−^ mice (*p* < 0.05) (Figures 6G,H). These results suggest the protective effect of TLB on EEIF-induced oxidative stress through mediation of the Nrf2/ferroptosis signaling pathway.

### 3.7 Effect of TLB on EEIF-Induced Oxidative Stress in the Myocardium of Nrf2^−/−^ Mice

**FIGURE 7:**
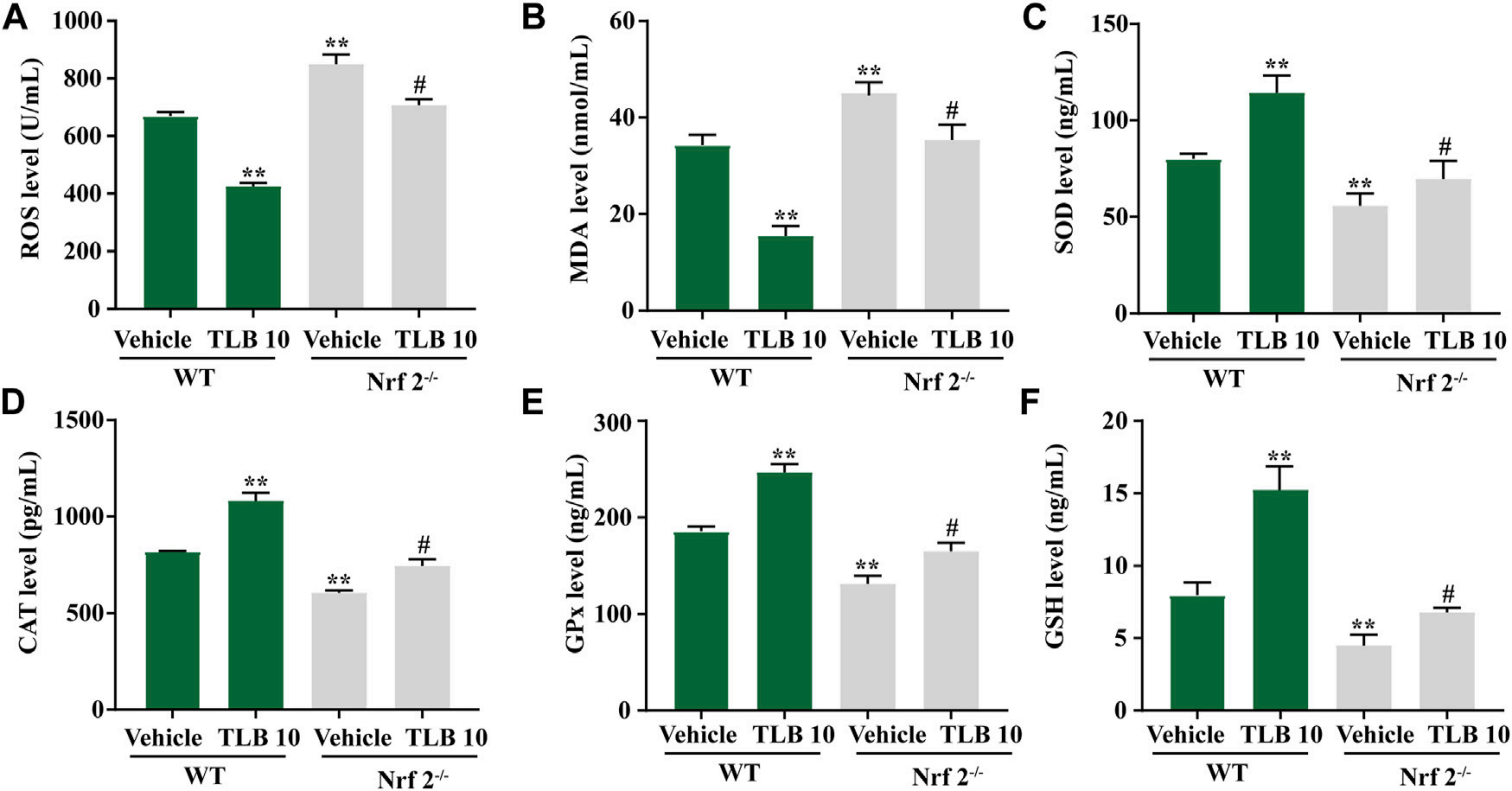
Effect of TLB on oxidative stress in heart of fatigue Nrf2−/− mice. **(A)** ROS level, **(B)** MDA level, **(C)** CAT activity, **(D)** SOD activity, **(E)** GPx activity, and **(F)** GSH level. The data were presented as the mean ± SEM (n = 10). ^*^ *p* < 0.05 and ^**^ *p* < 0.01 vs vehicle group. ^#^ *p* < 0.05 vs Nrf2^−/−^ vehicle group.

To further investigate the role of Nrf2 on the protective effects of TLB on EEIF-induced oxidative stress in the myocardium, we tested indicators of oxidative stress in Nrf2^−/−^ mice using an ELISA assay. Similar to the results in skeletal muscle, ROS and MDA levels in mice were clearly elevated in the Nrf2^-/-^-vehicle group compared with the WT-vehicle group, while the inhibitory effects of TLB on ROS and MDA were almost counteracted in Nrf2^−/−^ mice (*p* < 0.05) (Figures 7A,B). Also, SOD, CAT, and GPx activities as well as the GSH content were markedly decreased in mice of the Nrf2^-/-^-vehicle group compared with the WT-vehicle group, whereas the promotive effects of TLB were substantially canceled in Nrf2^−/−^ mice (*p* < 0.05) (Figures 7C–F). These findings confirm that Nrf2 not only plays a crucial role in the beneficial effect of TLB in skeletal muscle but also in the myocardium after EEIF insult.

## 4 Discussion

**FIGURE 8:**
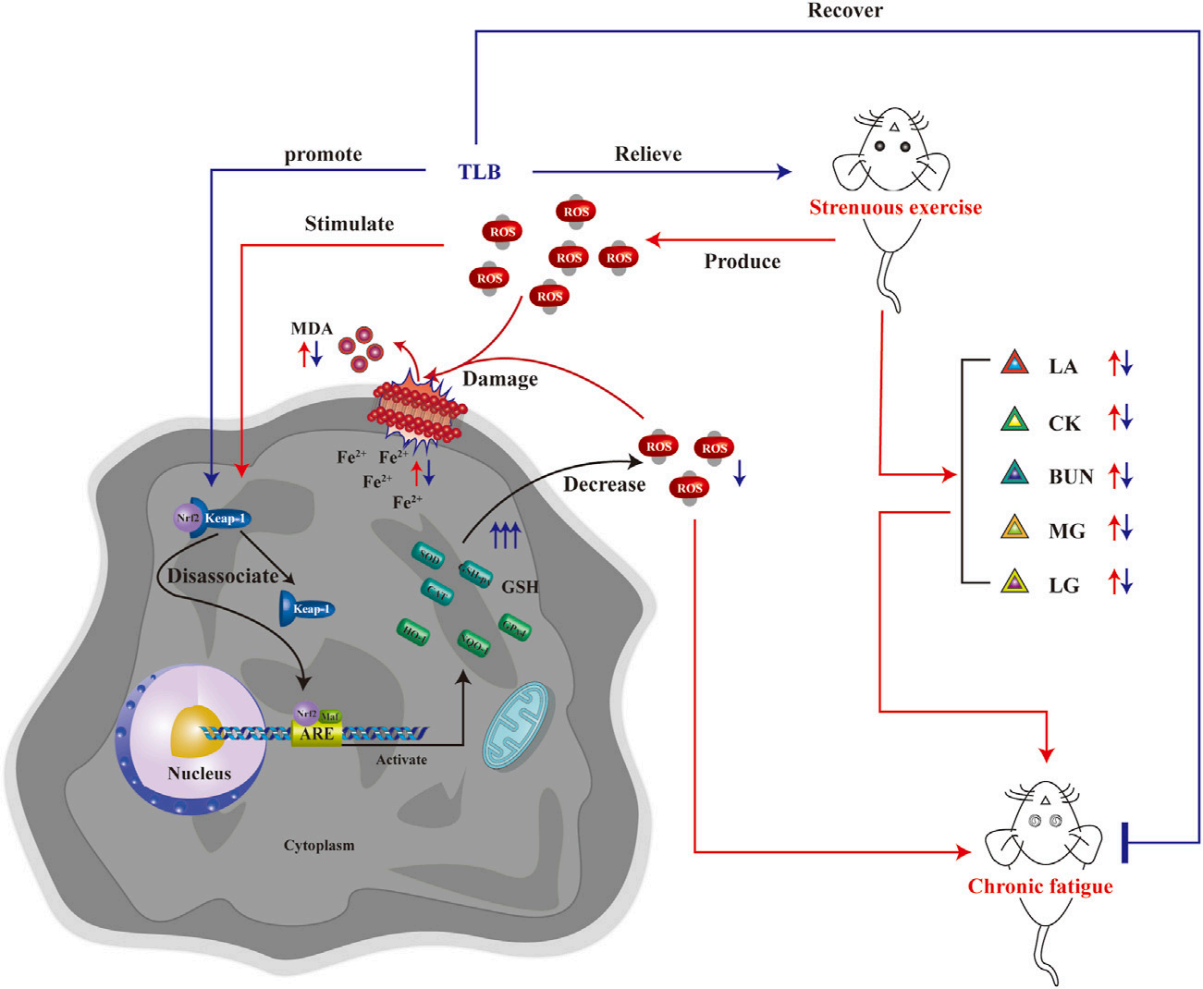
Schematic illustration of molecular mechanisms for the protective effect of TLB on EEIF in mice. TLB exerts a potent protective effect on EEIF in mice through mediating the Nrf2/ARE/ferroptosis signaling pathway, thereby suppressing oxidative stress.

The current study revealed that the following: 1) TLB, a naturally occurring food additive, exerted potent anti-EEIF effect in mice with a splendid safety profile, and 2) the beneficial effect of TLB on EEIF through mediating Nrf2/ARE/ferroptosis, thereby reducing oxidative stress (Figure 8). Corporately, our findings uncover the latent therapeutic target of TLB against EEIF and put forward the “proof-of-concept” for TLB to treat fatigue.

As safer and more effective drugs were definitely desired in the clinic, we focus on the TLB, a naturally occurring sweetener and approved as a food additive with outstanding safety property. First, we observed the effect of TLB on EEIF in mice by performing a weight-bearing swimming test and rope-climbing endurance test, which are the measurement indicators that directly reflect the exercise ability and the anti-fatigue capacity of drugs (Jiao et al., 2021). As we expected, the results in the present study indicated that TLB at doses of 5 and 10 mg/kg significantly prolonged exhaustive swimming time and the rope-climbing dwell time of mice, in keeping with our previous finding that TLB act as a food additive against diabetes at the doses of 10 mg/kg (Shi et al., 2022). In addition, the consumption of energy substances and the accumulation of metabolites occur during EEIF. Among those biomarkers, LA is an important indicator of the anaerobic glucose metabolism and is the end product of glycolysis during high-intensity exercise. During EEIF, LA was accumulated in the blood (Shen et al., 2021). Likewise, EEIF leads to the overproduction of BUN, which is a major factor in protein consumption in the body (Zhang et al., 2021b). CK is an important biomarker of muscle damage and increases significantly with EEIF (Chen et al., 2020b). LG and MG, which act as a substrate source for glycolysis and energy production, are also key indicators of EEIF (Chen et al., 2021). Our results showed that the change of biomarkers as aforementioned after EEIF challenge was reversed by TLB. These findings demonstrate that TLB exerts anti-fatigue effect and promotes eliminating detrimental metabolites as well as recovers the body energy.

Scientific focus on exercise-induced ROS production is whether it is friend or foe? Theoretically, exercise-induced ROS production is considered as a double-edged sword, whereby an appropriate generation of ROS during exercise facilitates skeletal muscles to better adapt to active physiological activity such as antioxidant enzymes synthesis, while excessive ROS production generates injury to proteins or DNA (Dan Dunn et al., 2015). Previous studies confirm that EEIF-induced oxidative stress occurs, which results in increase of oxidative stress indicator (e.g., ROS and MDA) productions and decrease in antioxidant enzymes activities, namely, SOD, CAT, and GPx, which are the three dominating antioxidant enzymes anchored in cells (Ma et al., 2022). SOD, a body’s first line defense against superoxide anion, can dismutate superoxide anion to form hydrogen peroxide and oxygen, which is vital to prevent oxidative injury (McCord and Edeas, 2005). Similar to SOD, CAT is also anchored in numerous cellular compartments, and its primary function is to eliminate hydrogen peroxide (Yi et al., 2021). Additionally, five subtypes of GPx exist in mammals (GPx1–GPx5), which are responsible for eliminating hydrogen peroxide or hydroperoxide to form water or alcohol (Martins Alves et al., 2020). Moreover, GPx reactions need an electron donor, and GSH is the dominating electron donor for these GPx reactions (Hsiao et al., 2021). As expected, our results demonstrated that TLB not only reduced the productions of ROS and MDA but also increased the activities of SOD, CAT, and GPx, as well as the level of GSH. These findings indicated that TLB effectively inhibited oxidative stress *via* promoting anti-oxidant enzyme activity to reduce excessive ROS production. Of note, the heart muscle is one of the most vulnerable tissues to oxidative stress, and therefore, we also investigated the anti-oxidant effect of TLB on the myocardium after EEIF insult. Intriguingly, our results showed that TLB efficiently inhibited oxidative injury as evidenced by a decrease in excessive ROS and an increase in antioxidant enzyme activities. Interestingly, since the Nrf2 signaling pathway is involved in EEIF-induced oxidative damage, Nrf2 might be a potential therapeutic target to treat EEIF. As expected, our results showed that TLB effectively promoted the disassociation of Keap1 and Nrf2 complex, facilitated the nuclear translocation of Nrf2, and accelerated the degradation of the inhibitory protein Keap-1, thereby activating its downstream genes HO-1 and NQO-1. These findings suggest the protective effect of TLB on EEIF, at least partly, through activating the Nrf2/ARE signaling pathway. However, the detailed mechanism by which TLB activates Nrf2 will be deciphered in our next study. Furthermore, we also determined the role of ferroptosis in the Nrf2-mediated anti-oxidative stress effect of TLB on EEIF. Ferroptosis, which is different from other forms of cell death (Zhang et al., 2022a), is associated with MDA accumulation and iron-overloading, and is negatively controlled by GPx4 (Zhang et al., 2022b), which is also a target of Nrf2 and a primary depressor for ferroptosis through reducing MDA and ROS production (Dong et al., 2021; Shi et al., 2022). As we expected, our results showed that TLB significantly up-regulated GPx4 protein expression and decreased the iron overload after EEIF insult. These findings indicate that the Nrf2/ferroptosis pathway is involved in the anti-fatigue effect of TLB, and its detailed mechanism is worth deciphering in depth.

Based on the findings of our previous studies and the results mentioned earlier, we thus speculated that Nrf2 might be a potential therapeutic target of TLB to alleviate EEIF. We next applied Nrf2^−/−^ mice to test our hypothesis. Interestingly, our results showed that Nrf2^−/−^ mice exhibited lower exhaustive swimming time and rope climbing time than WT mice, which confirmed that Nrf2 plays a crucial role during EEIF. However, the beneficial effects of TLB on the aforementioned metrics were largely abolished in Nrf2^−/−^ mice. Moreover, the effect of TLB on the biochemical indexes was also reversed by TLB. These findings verify that TLB exerted anti-EEIF effect *via* targeting Nrf2. Next, we also found that oxidative stress was aggravated and anti-oxidant enzyme activities were dramatically decreased in Nrf2^−/−^ mice, in keeping with previous studies (Zhang et al., 2022c). However, the beneficial effects of TLB on EEIF-induced oxidative stress were substantially offset in Nrf2^−/−^ mice. Most interestingly, HO-1 when activated will increase the release of free iron, and it has been reported in previous studies (Fernández-Mendívil et al., 2021). However, recent research also found that activation of the Nrf2/HO-1 pathway can inhibit ferroptosis (Wang et al., 2022; Yang et al., 2022). Thus, we then explored the relationship between Nrf2 and ferroptosis, and confirmed whether the Nrf2/ARE/ferroptosis was involved in the anti-fatigue effect of TLB. Encouragingly, we discovered that the GPx4 level was markedly reduced and iron overload was significantly increased in Nrf2^−/−^ mice after EEIF challenge, which suggested that Nrf2/ARE/ferroptosis was involved in fatigue. However, the effect of TLB on the GPx4 level and iron overload after EEIF insult was dramatically abrogated, confirming that TLB protected against EEIF *via* mediating the Nrf2/ARE/ferroptosis axis.

Of note, the study preliminarily reveals that TLB effectively relieved EEIF through mediation of Nrf2/ARE/ferroptosis.

Although we achieve exciting experimental evidence, there are still outstanding questions to be resolved in the future. First, albeit we offer direct proof that TLB exerts potent anti-fatigue, its detailed mechanism or exact therapeutic target remains unclear. Second, Nrf2/ARE/ferroptosis plays a crucial role in the beneficial effect of TLB on fatigue; whether TLB can be developed as a naturally occurring Nrf2 activator is worth to probe in our next story.

In conclusion, we report, for the first time, that TLB effectively alleviated EEIF-induced oxidative stress in mice by mediating the Nrf2/ferroptosis signaling pathway. It can be deduced that TLB might be a powerful agent with an excellent safety profile to overcome fatigue.

## Data Availability

The original contributions presented in the study are included in the article/Supplementary Materials; further inquiries can be directed to the corresponding author.
