# Supplementary figures and images for: Trilobatin, a Naturally Occurring Food Additive, Ameliorates Exhaustive Exercise-Induced Fatigue in Mice: Involvement of Nrf2/ARE/Ferroptosis Signaling Pathway
Source: Front Pharmacol. 2022 Jun 24;13:913367. doi: 10.3389/fphar.2022.913367 (PMC9263197; doi:10.3389/fphar.2022.913367)

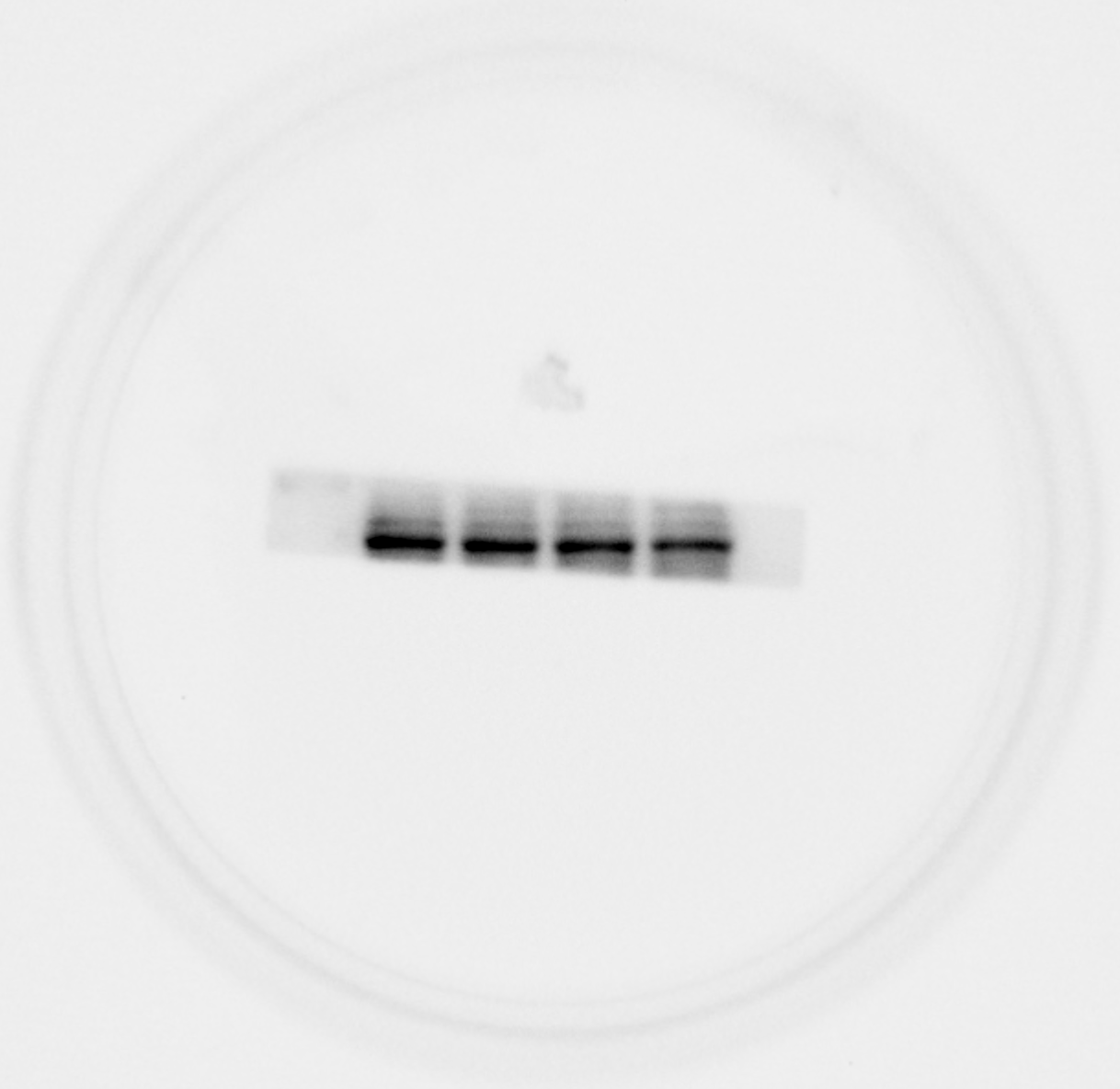

Supplement: Supplementary file 1 [file DataSheet1.ZIP › Oringinal western blots/Cytoplasm-Nrf2/Cytoplasm-Nrf2/Cytoplasm-Nrf2 (1).tif]

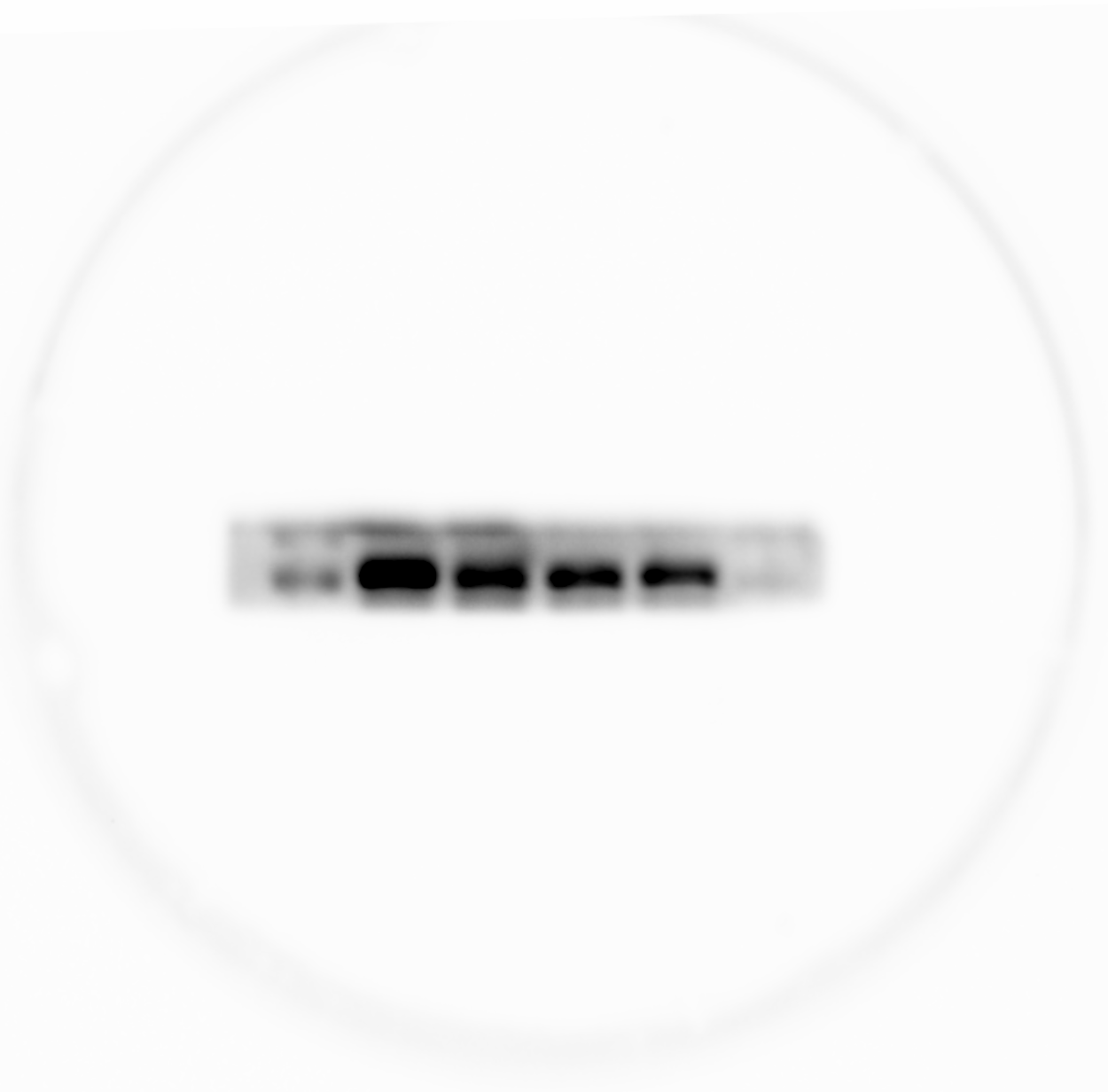

Supplement: Supplementary file 1 [file DataSheet1.ZIP › Oringinal western blots/Cytoplasm-Nrf2/Cytoplasm-Nrf2/Cytoplasm-Nrf2 (2).tif]

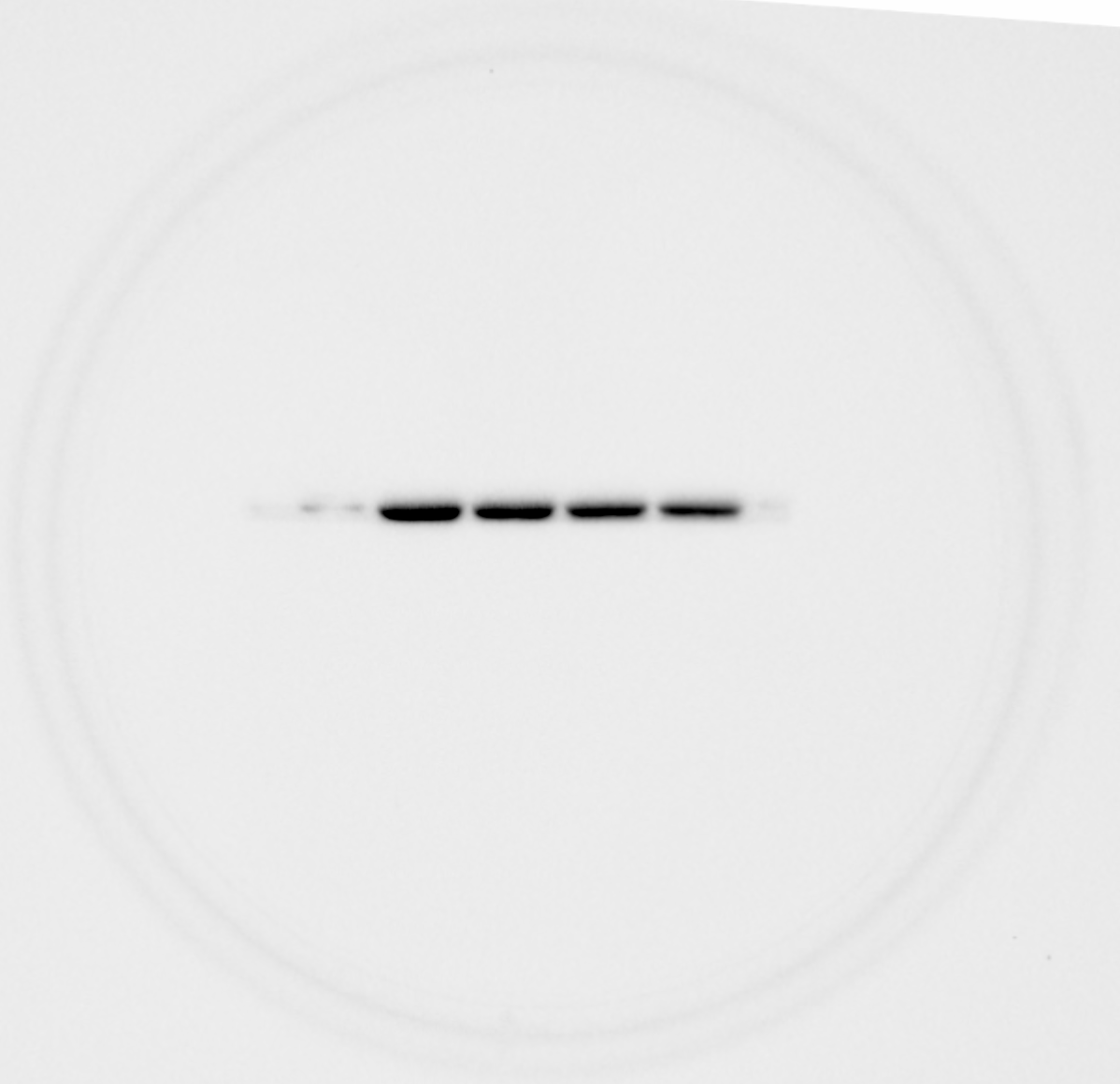

Supplement: Supplementary file 1 [file DataSheet1.ZIP › Oringinal western blots/Cytoplasm-Nrf2/Cytoplasm-Nrf2/Cytoplasm-Nrf2 (3).tif]

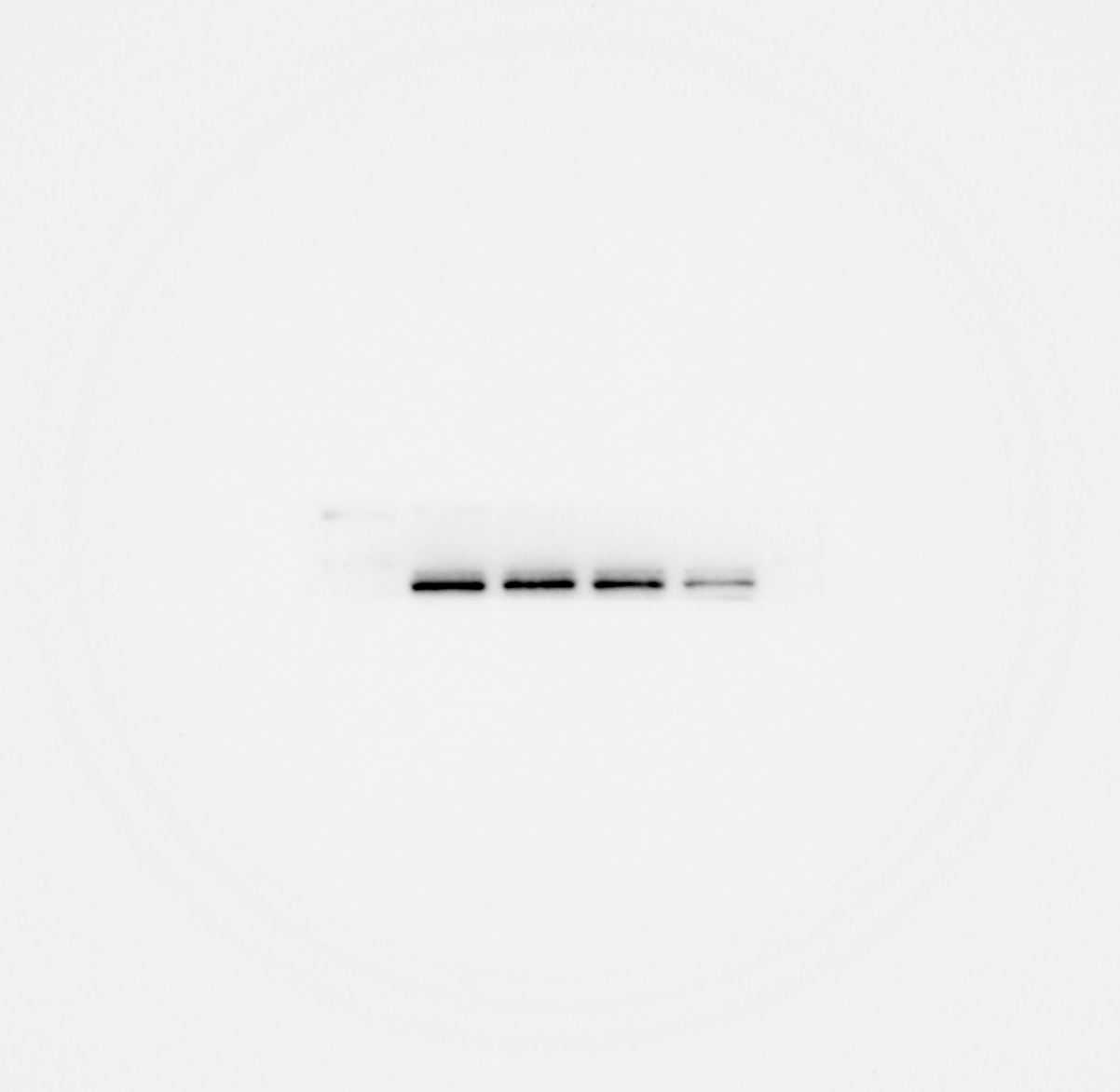

Supplement: Supplementary file 1 [file DataSheet1.ZIP › Oringinal western blots/Cytoplasm-Nrf2/Cytoplasm-Nrf2/Cytoplasm-Nrf2 (4).tif]

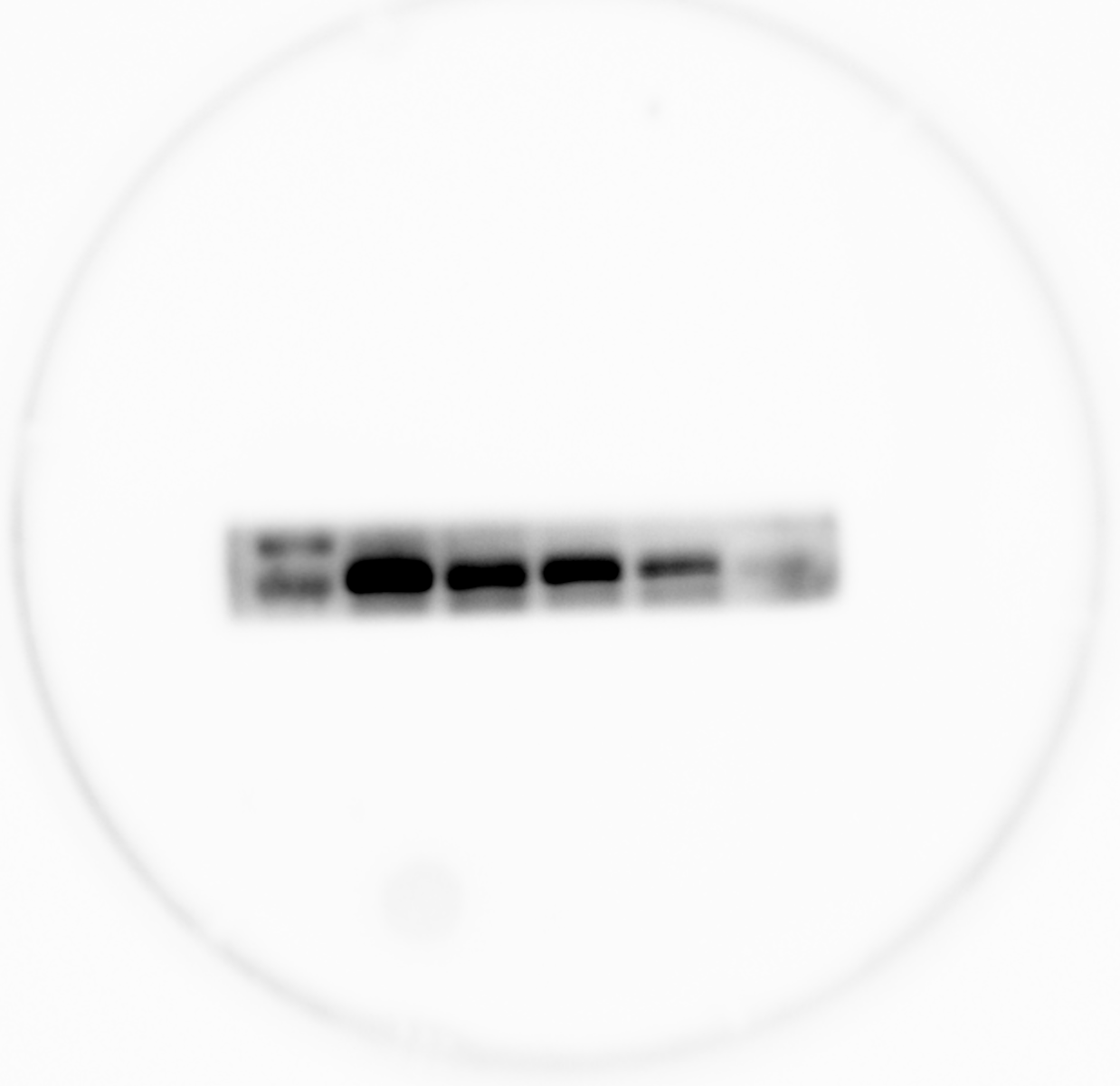

Supplement: Supplementary file 1 [file DataSheet1.ZIP › Oringinal western blots/Cytoplasm-Nrf2/Cytoplasm-Nrf2/Cytoplasm-Nrf2 (5).tif]

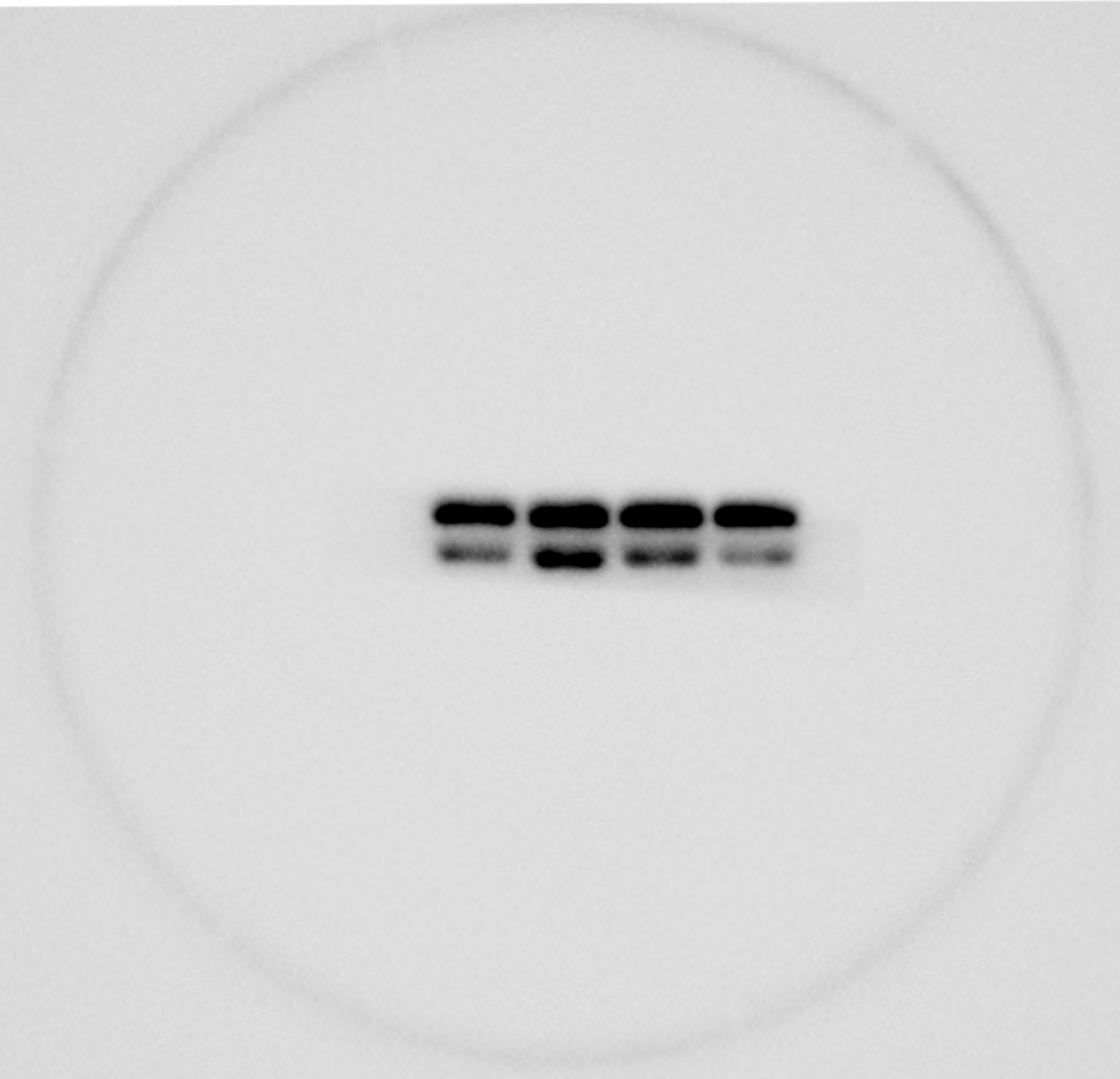

Supplement: Supplementary file 1 [file DataSheet1.ZIP › Oringinal western blots/Cytoplasm-Nrf2/a┬-actin/a┬-actin (1).tif]

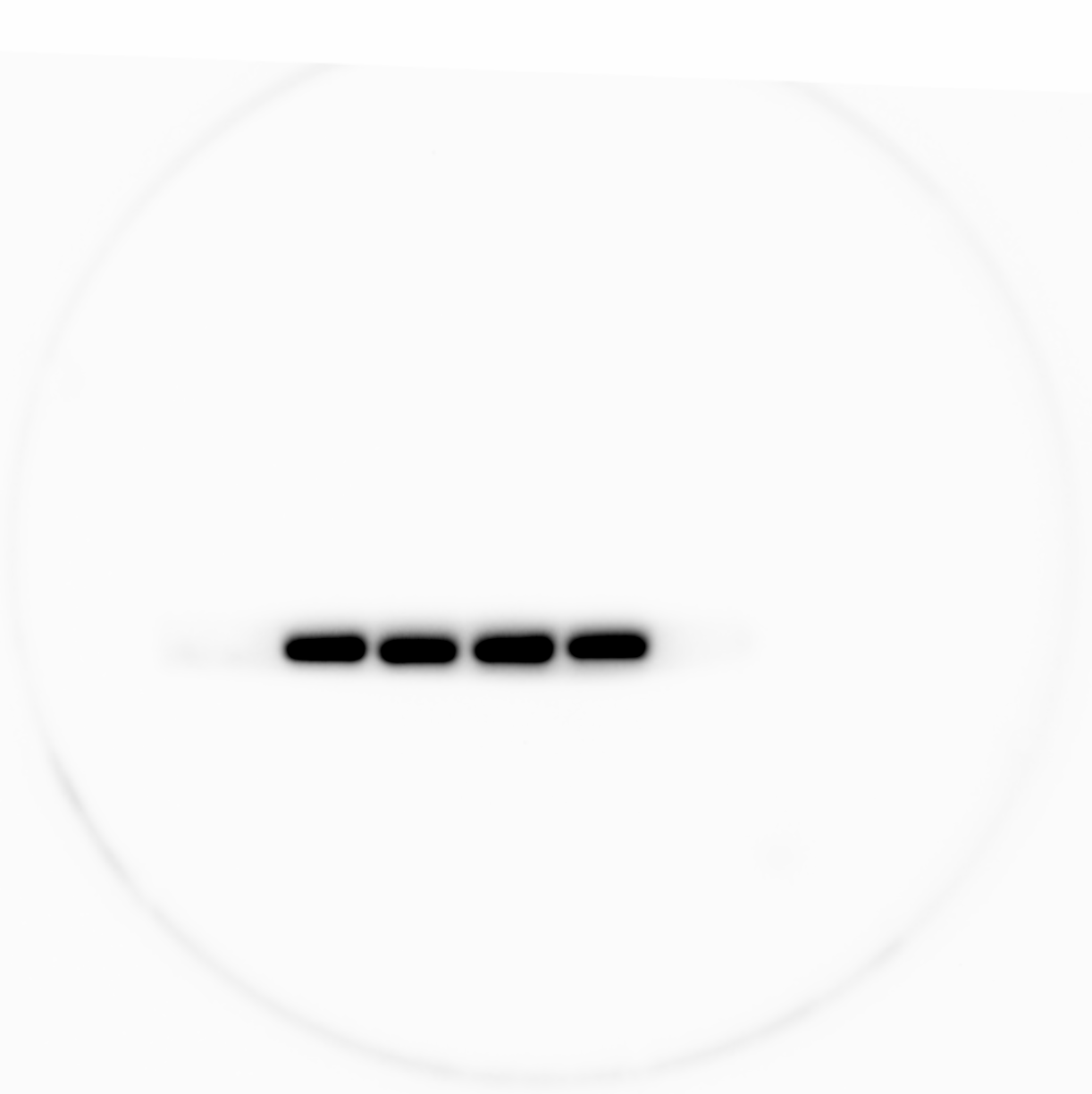

Supplement: Supplementary file 1 [file DataSheet1.ZIP › Oringinal western blots/Cytoplasm-Nrf2/a┬-actin/a┬-actin (2).tif]

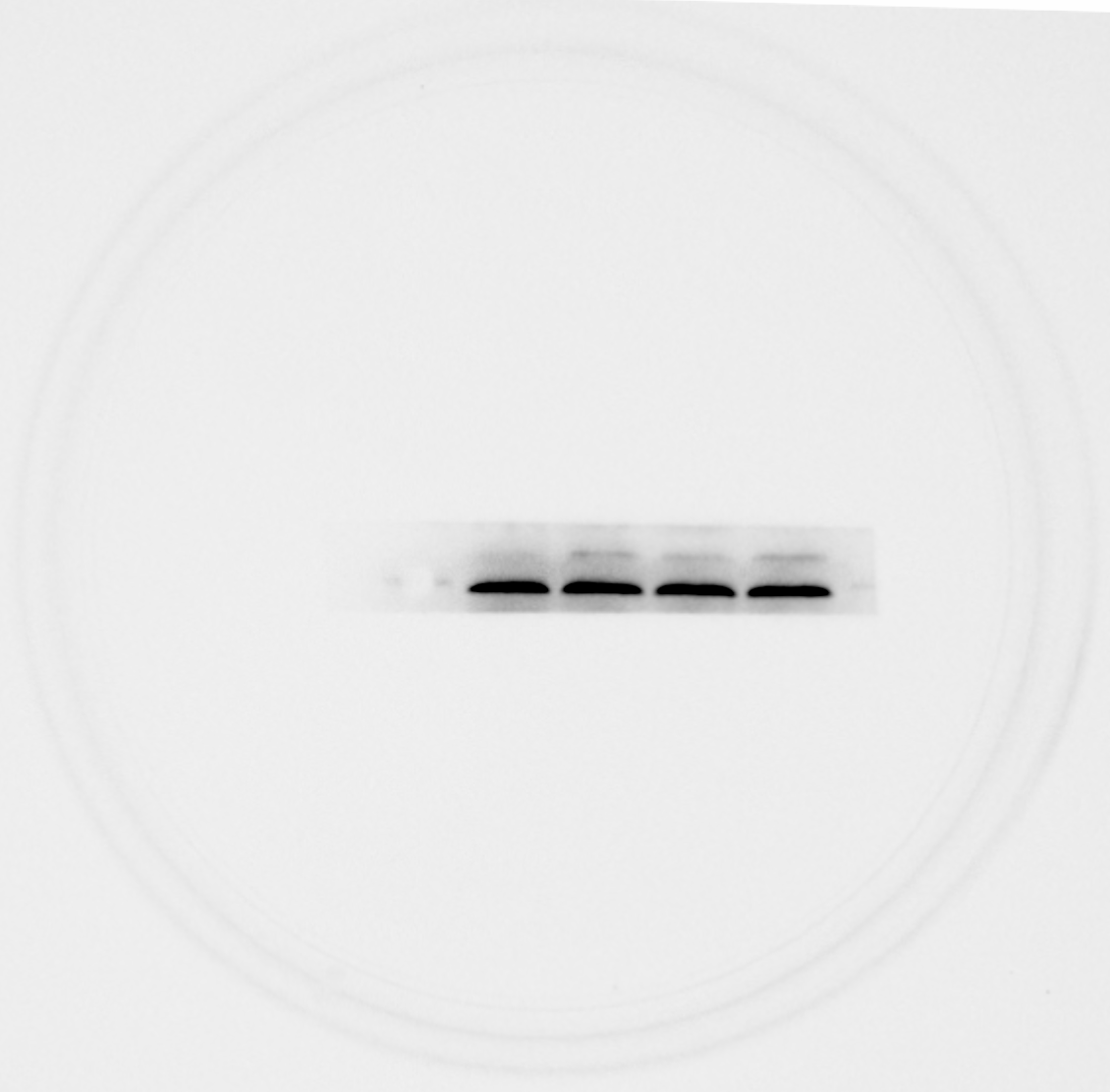

Supplement: Supplementary file 1 [file DataSheet1.ZIP › Oringinal western blots/Cytoplasm-Nrf2/a┬-actin/a┬-actin (3).tif]

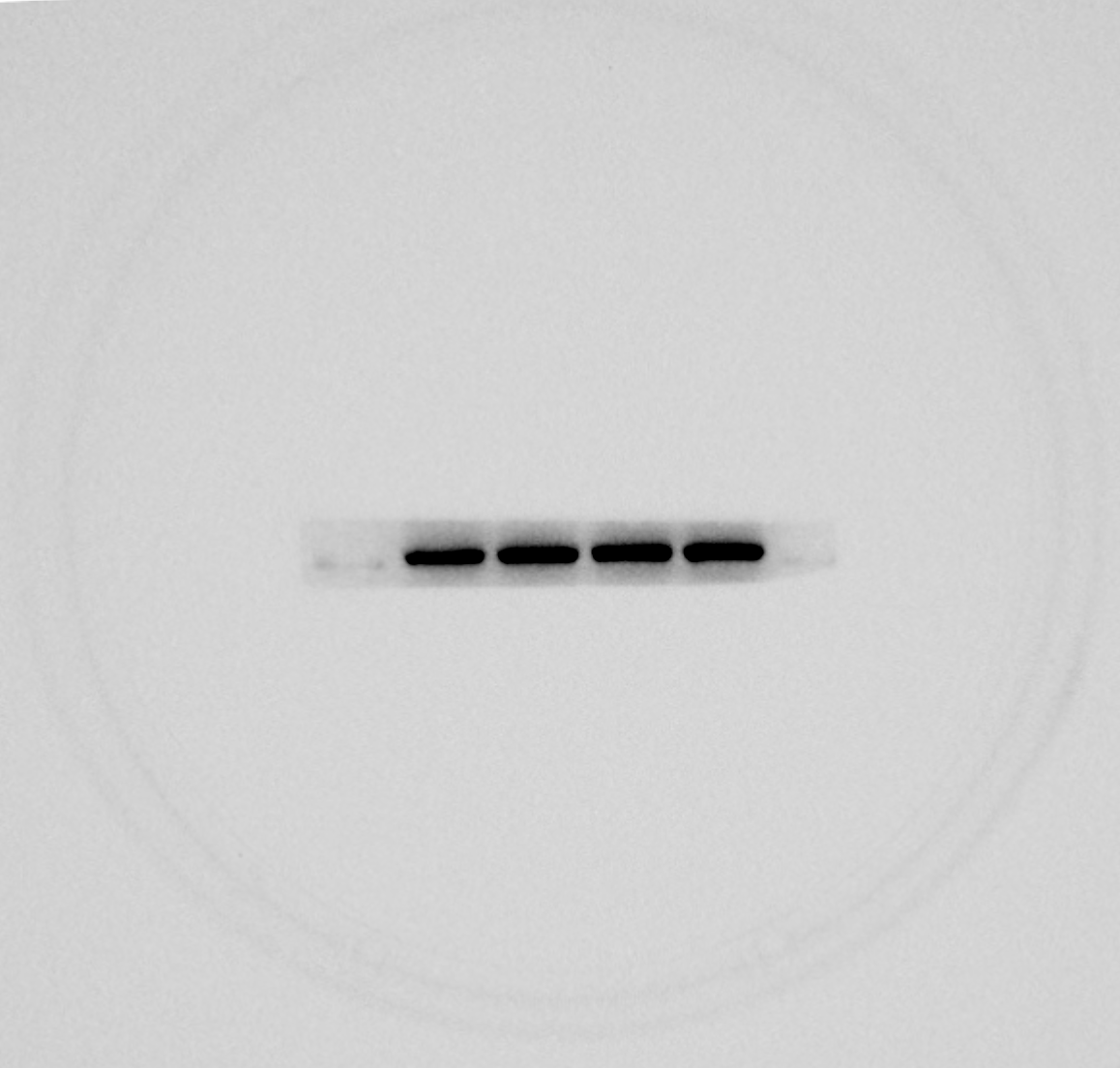

Supplement: Supplementary file 1 [file DataSheet1.ZIP › Oringinal western blots/Cytoplasm-Nrf2/a┬-actin/a┬-actin (4).tif]

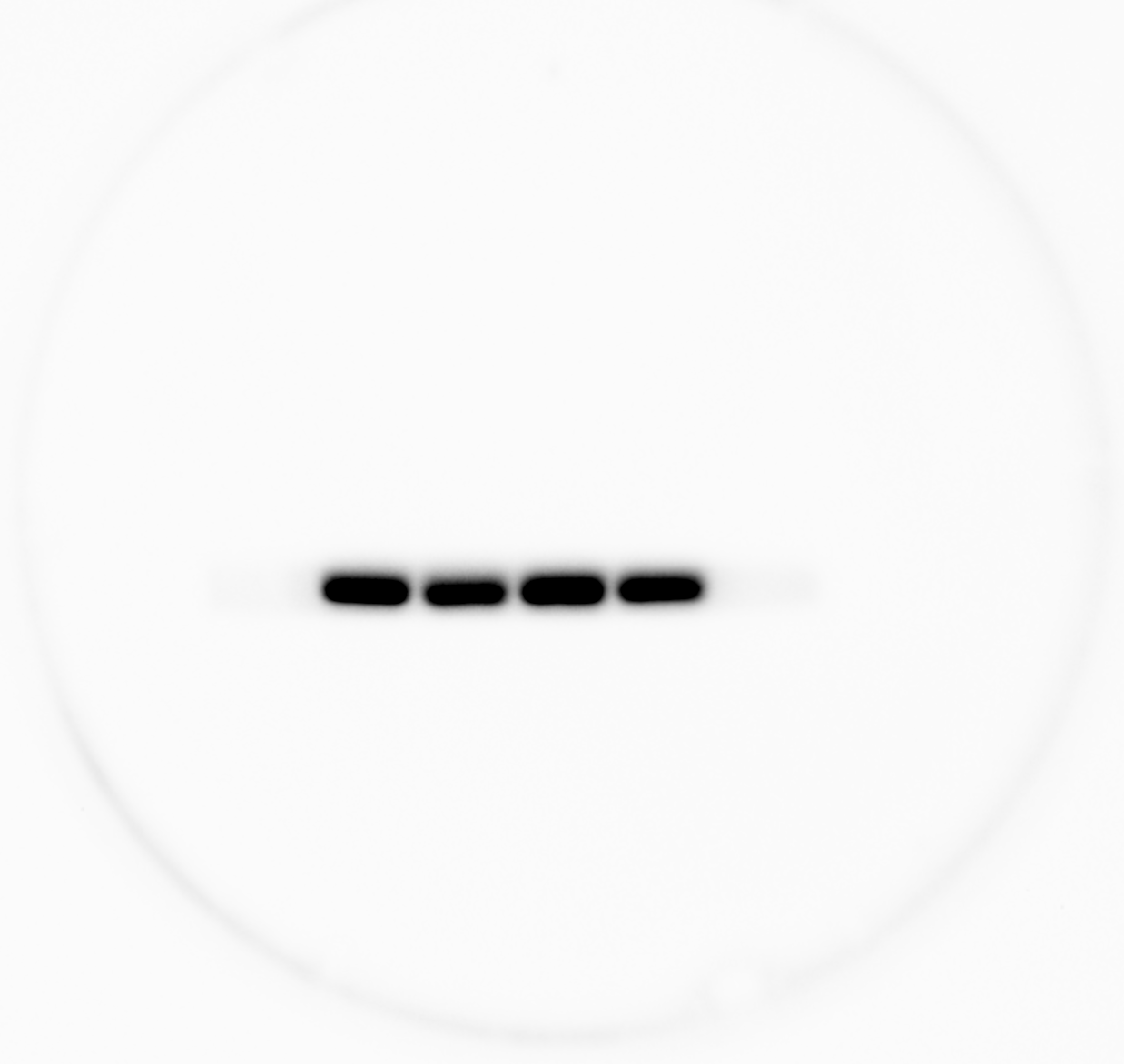

Supplement: Supplementary file 1 [file DataSheet1.ZIP › Oringinal western blots/Cytoplasm-Nrf2/a┬-actin/a┬-actin (5).tif]

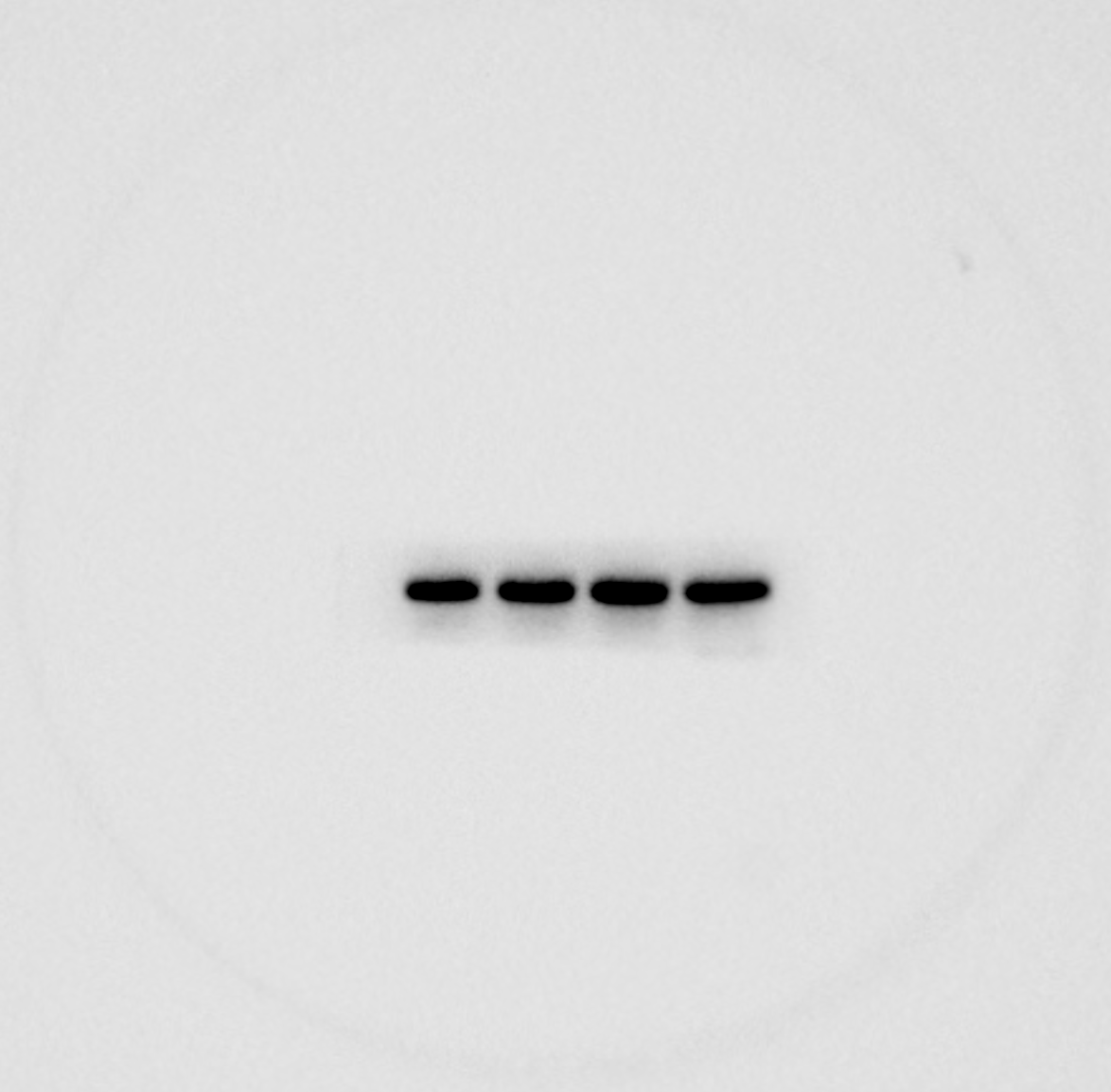

Supplement: Supplementary file 1 [file DataSheet1.ZIP › Oringinal western blots/GPx4/GAPDH/GAPDH (1).tif]

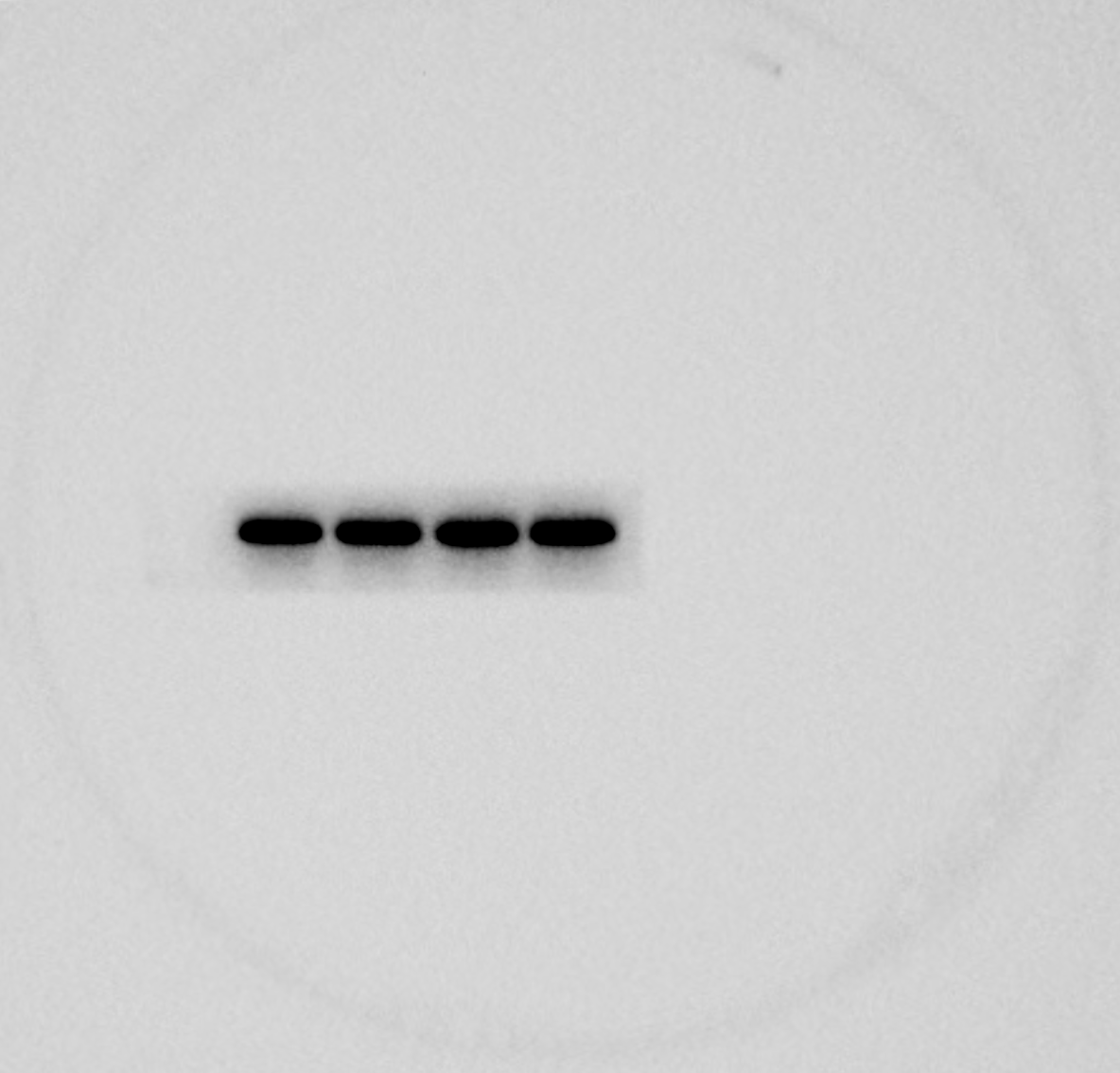

Supplement: Supplementary file 1 [file DataSheet1.ZIP › Oringinal western blots/GPx4/GAPDH/GAPDH (2).tif]

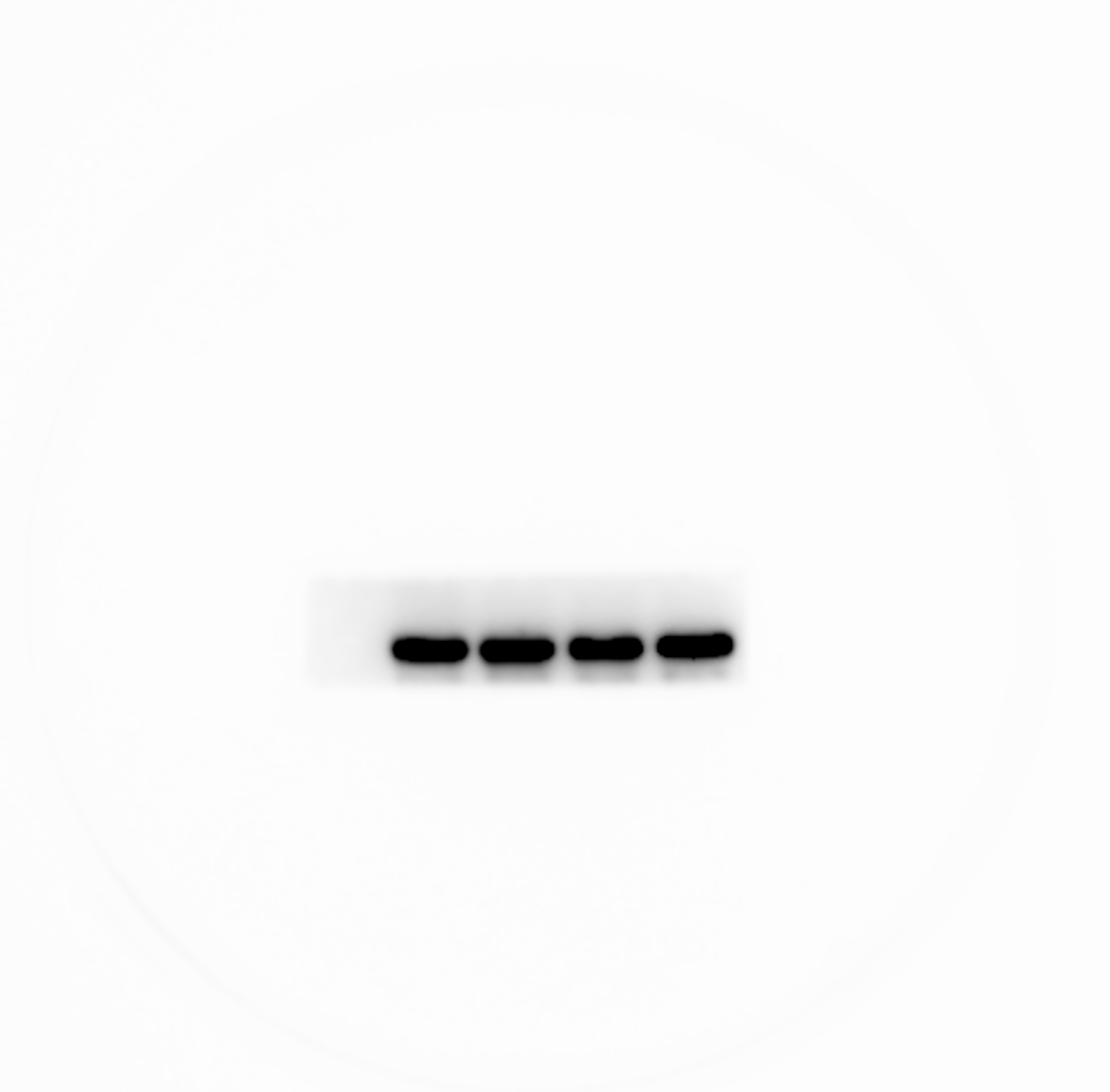

Supplement: Supplementary file 1 [file DataSheet1.ZIP › Oringinal western blots/GPx4/GAPDH/GAPDH (3).tif]

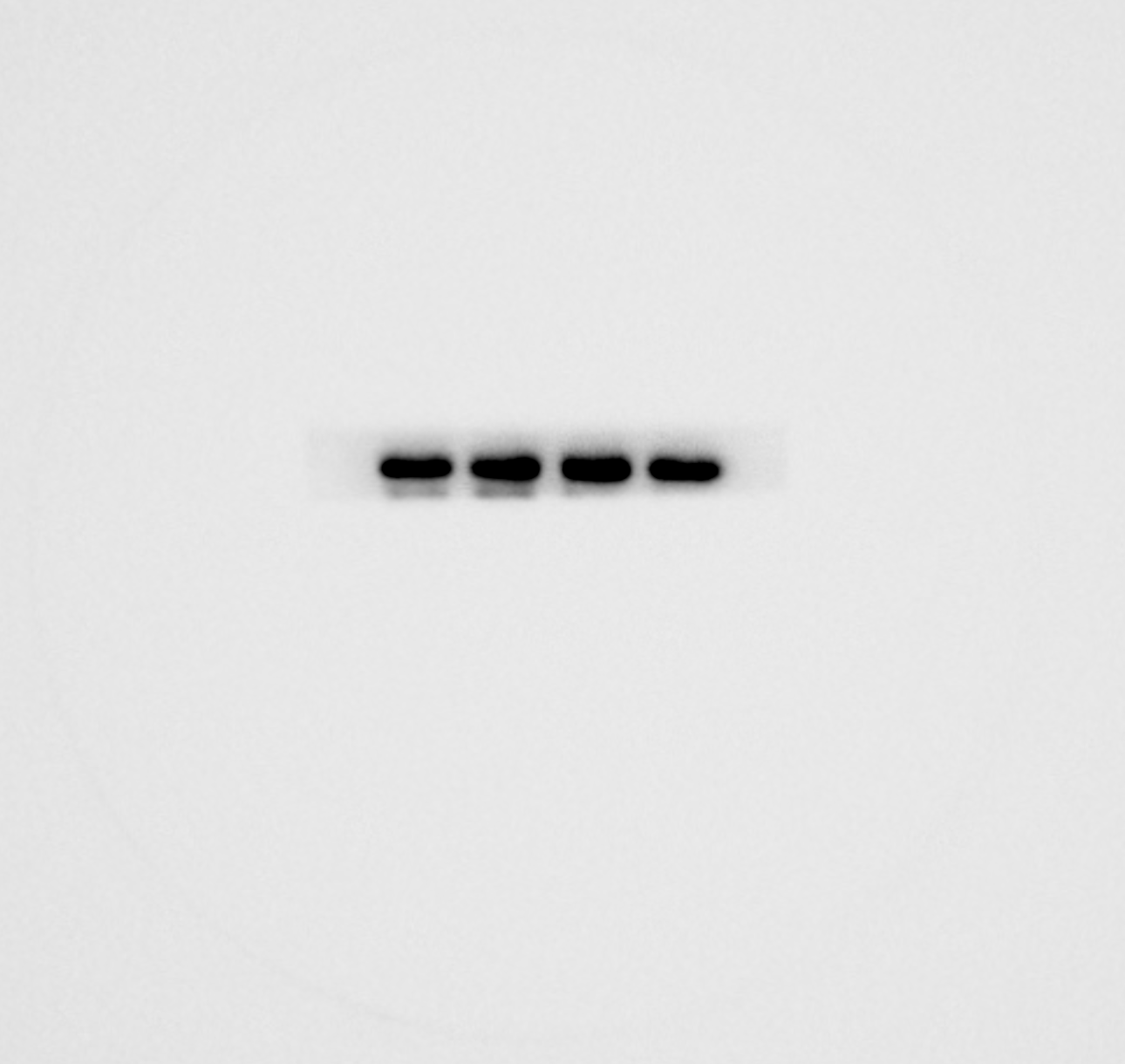

Supplement: Supplementary file 1 [file DataSheet1.ZIP › Oringinal western blots/GPx4/GAPDH/GAPDH (4).tif]

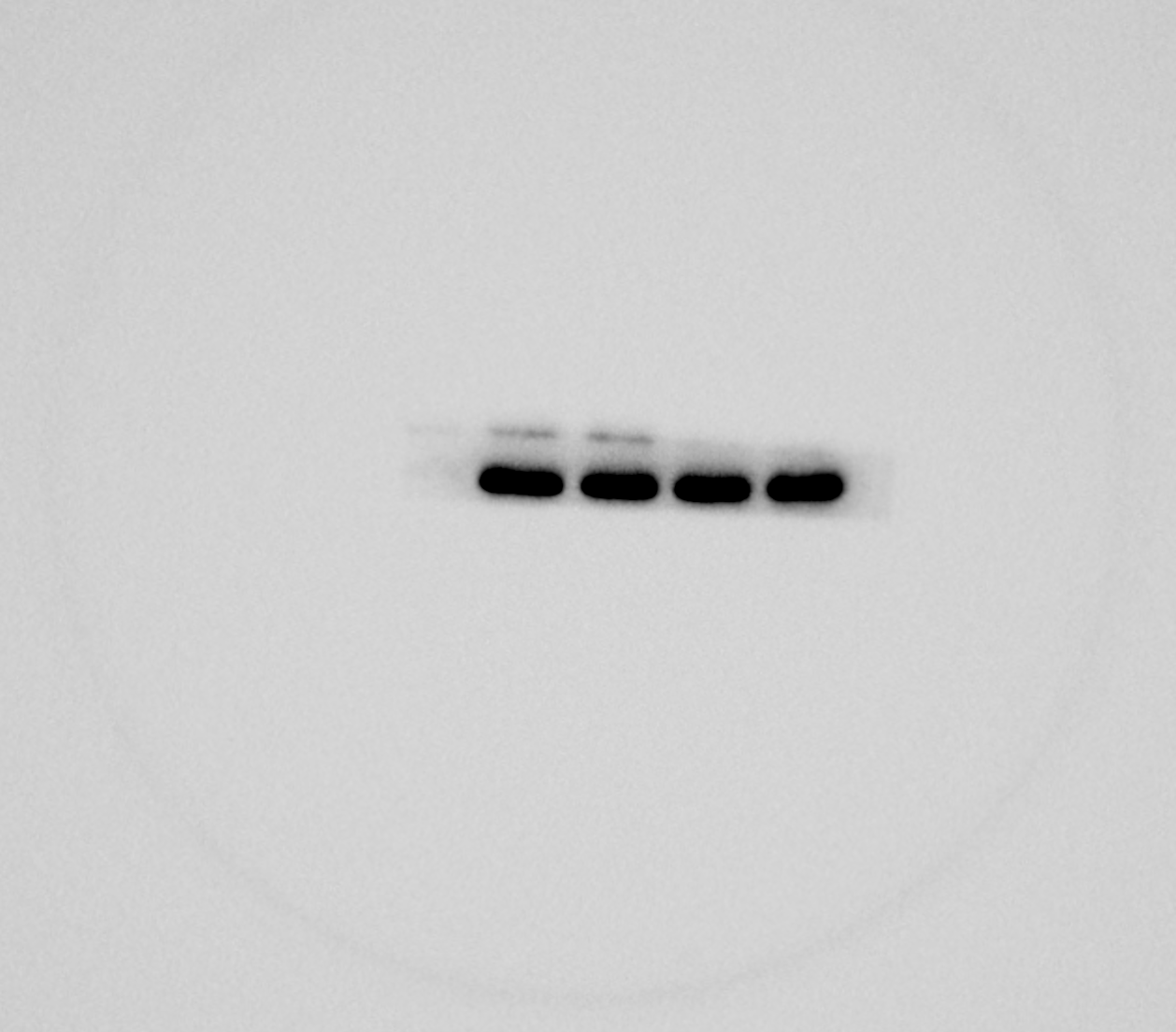

Supplement: Supplementary file 1 [file DataSheet1.ZIP › Oringinal western blots/GPx4/GAPDH/GAPDH (5).tif]

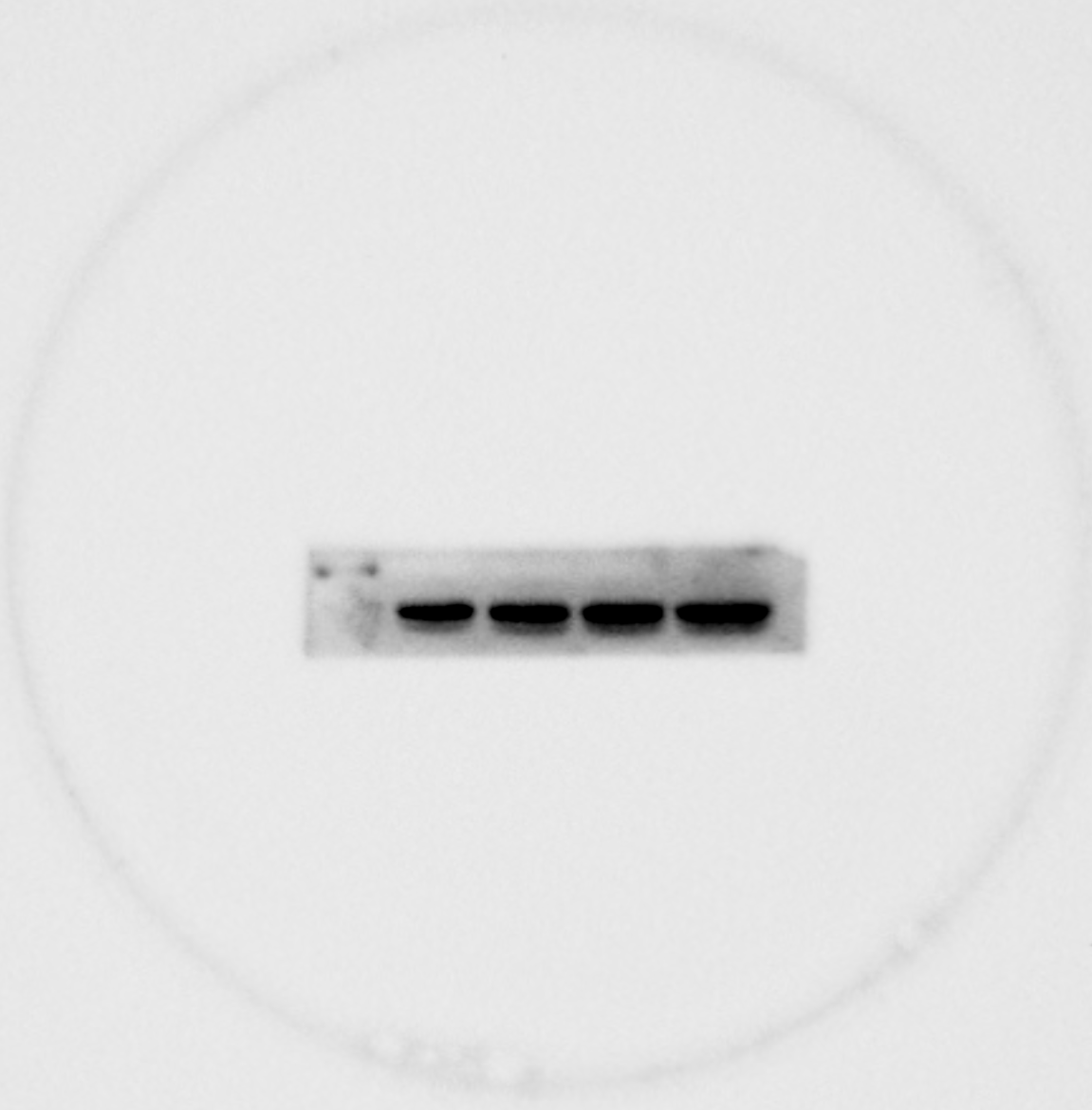

Supplement: Supplementary file 1 [file DataSheet1.ZIP › Oringinal western blots/GPx4/GPx4/GPx4 (1).tif]

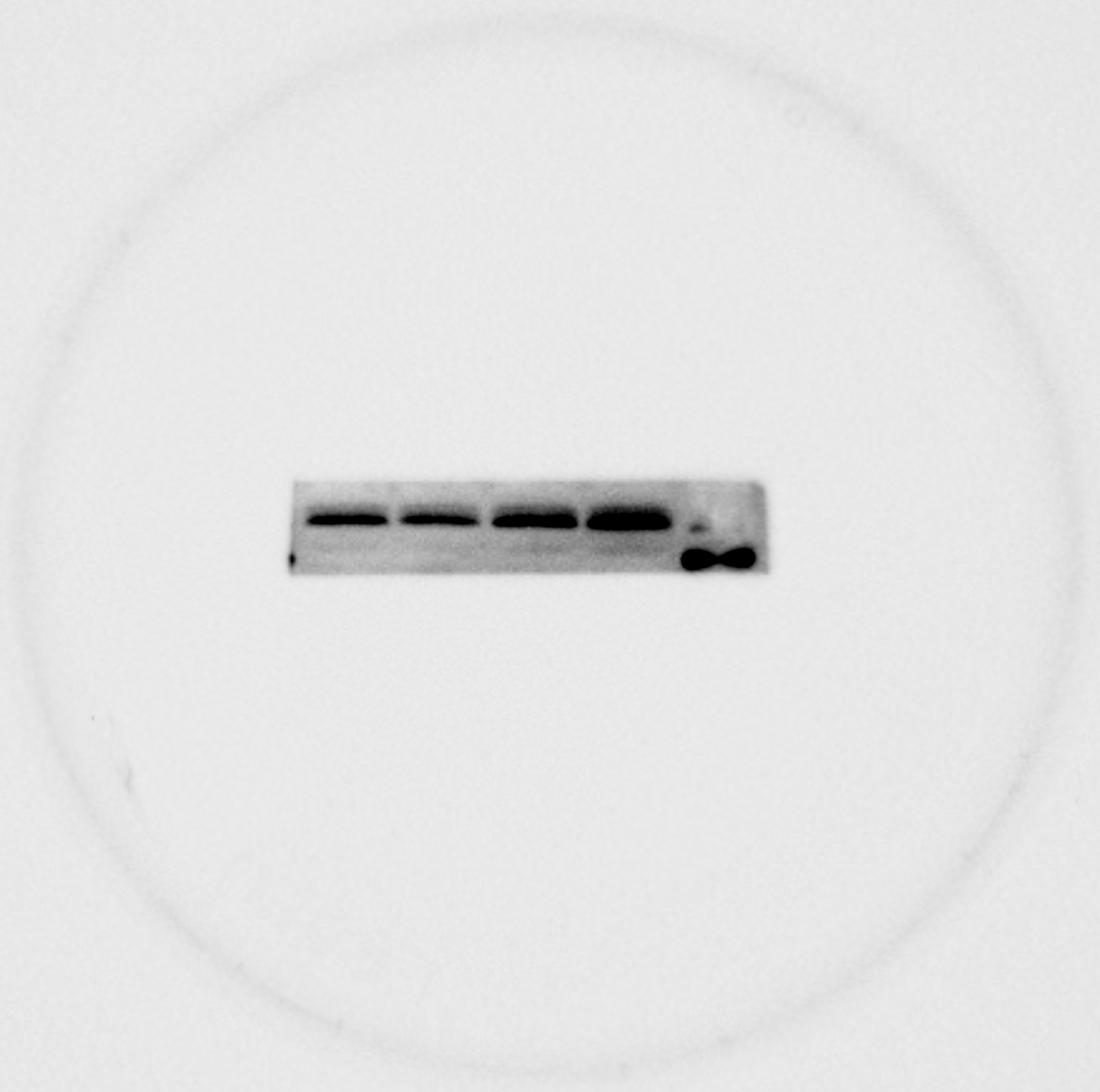

Supplement: Supplementary file 1 [file DataSheet1.ZIP › Oringinal western blots/GPx4/GPx4/GPx4 (2).tif]

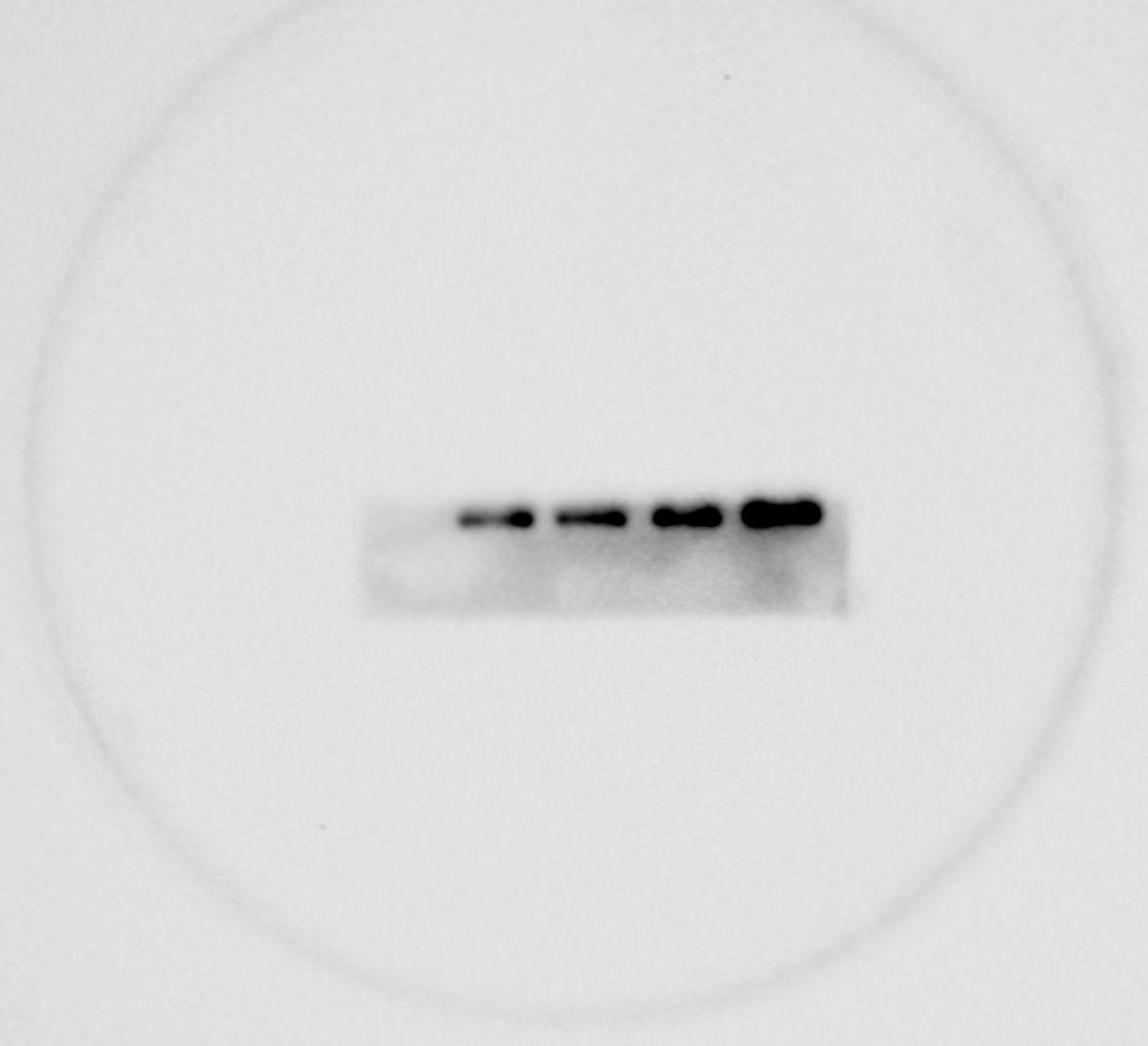

Supplement: Supplementary file 1 [file DataSheet1.ZIP › Oringinal western blots/GPx4/GPx4/GPx4 (3).tif]

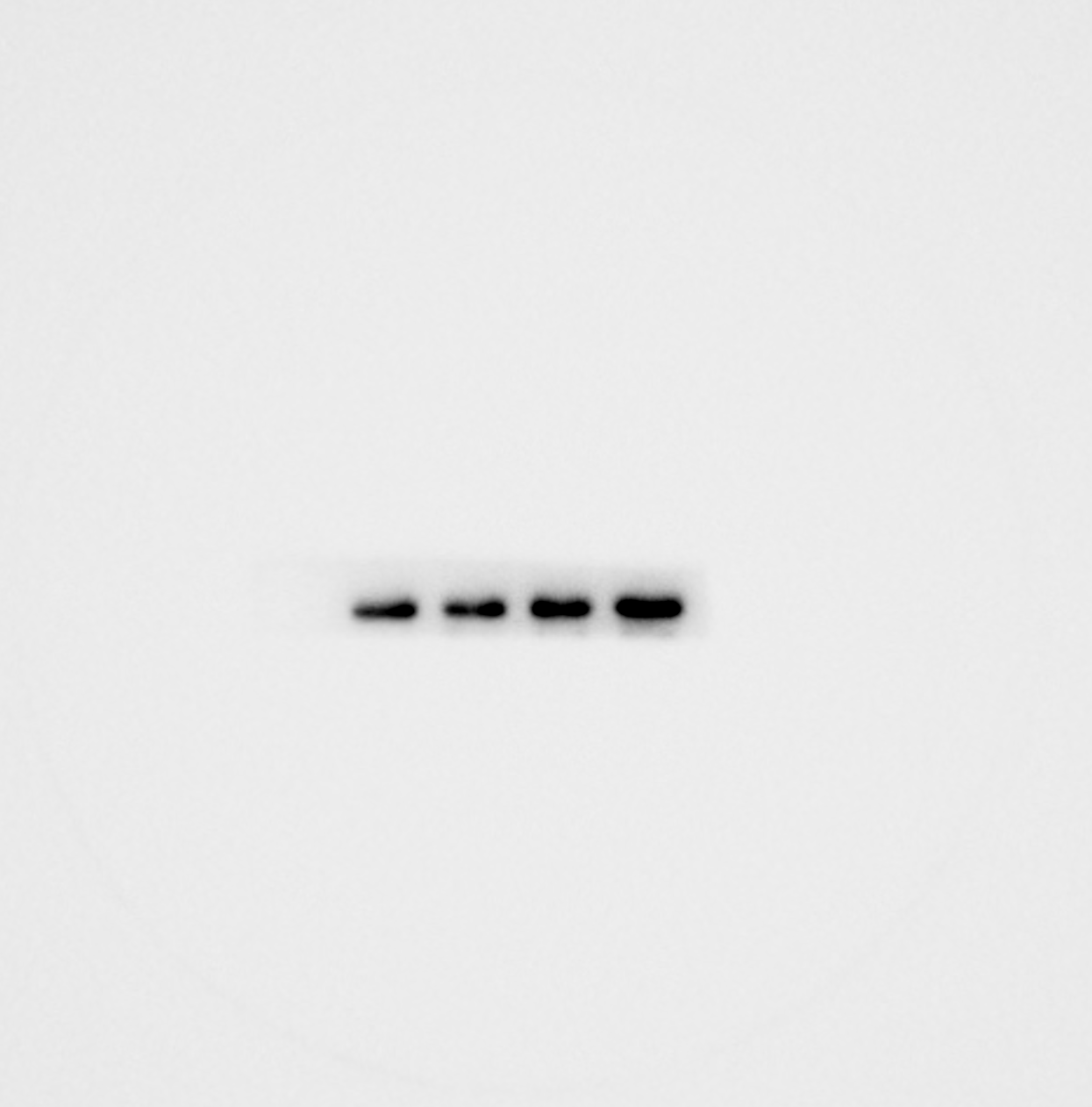

Supplement: Supplementary file 1 [file DataSheet1.ZIP › Oringinal western blots/GPx4/GPx4/GPx4 (4).tif]

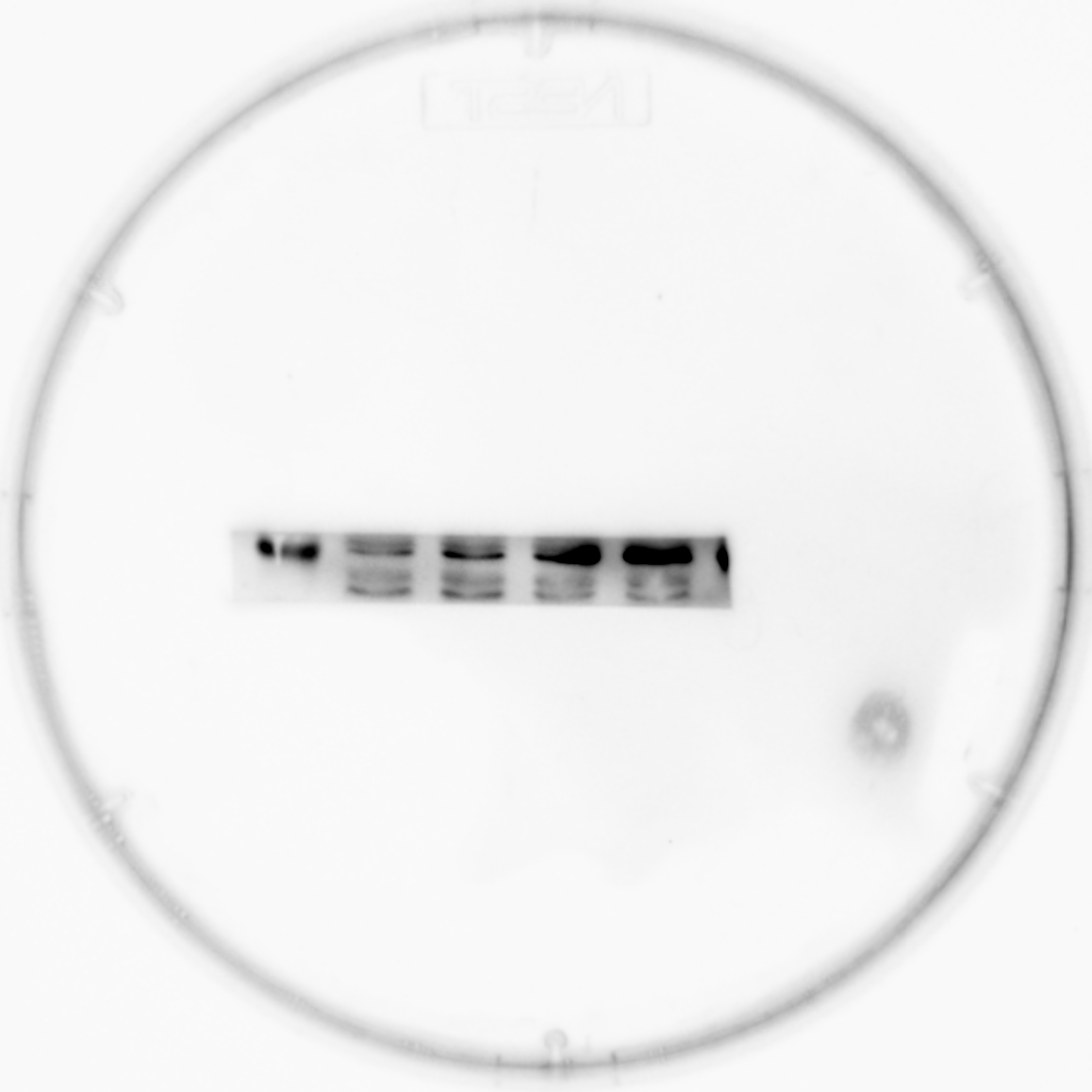

Supplement: Supplementary file 1 [file DataSheet1.ZIP › Oringinal western blots/GPx4/GPx4/Gpx (5).tif]

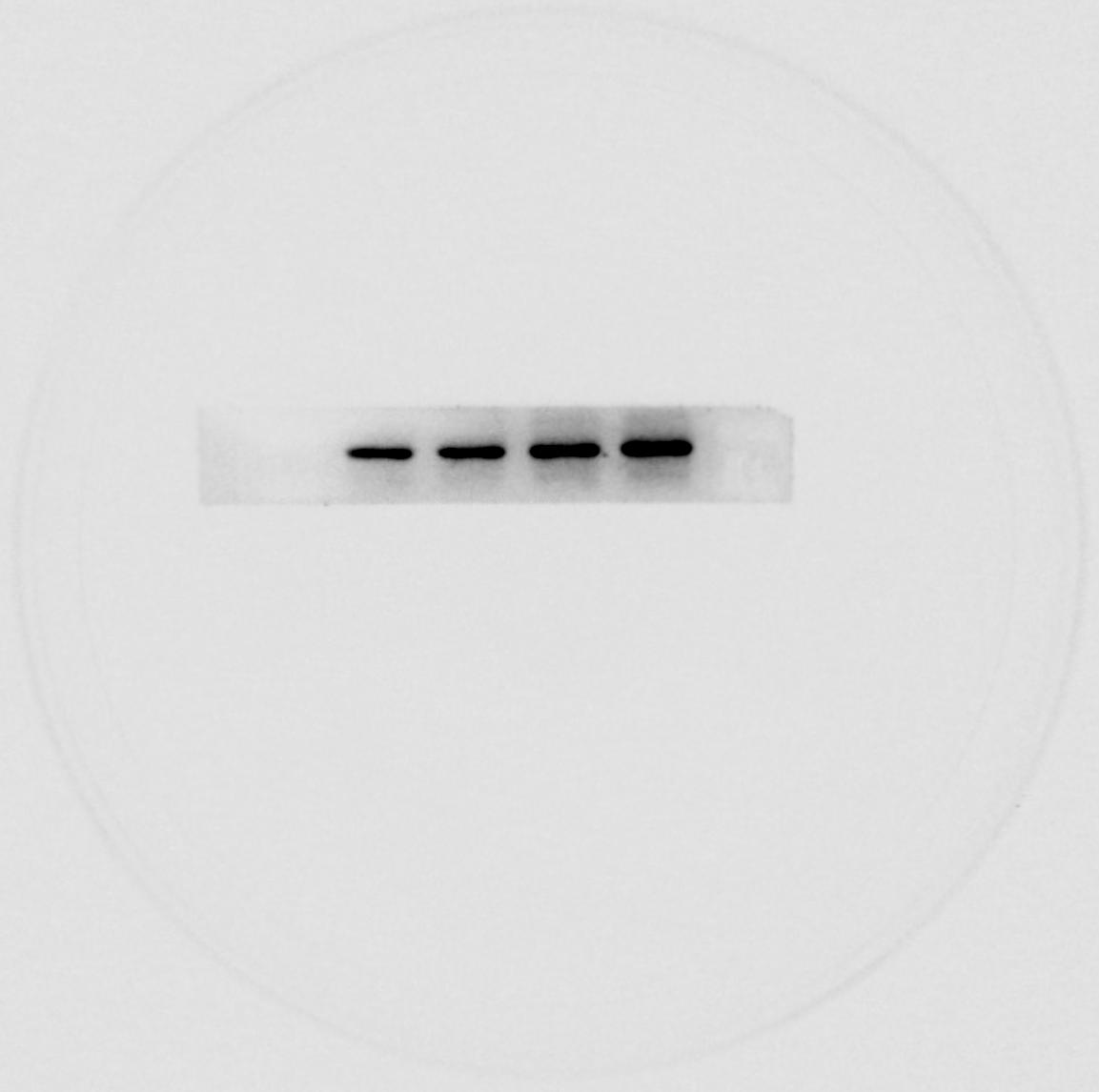

Supplement: Supplementary file 1 [file DataSheet1.ZIP › Oringinal western blots/HO-1/HO-1/HO-1 (1).tif]

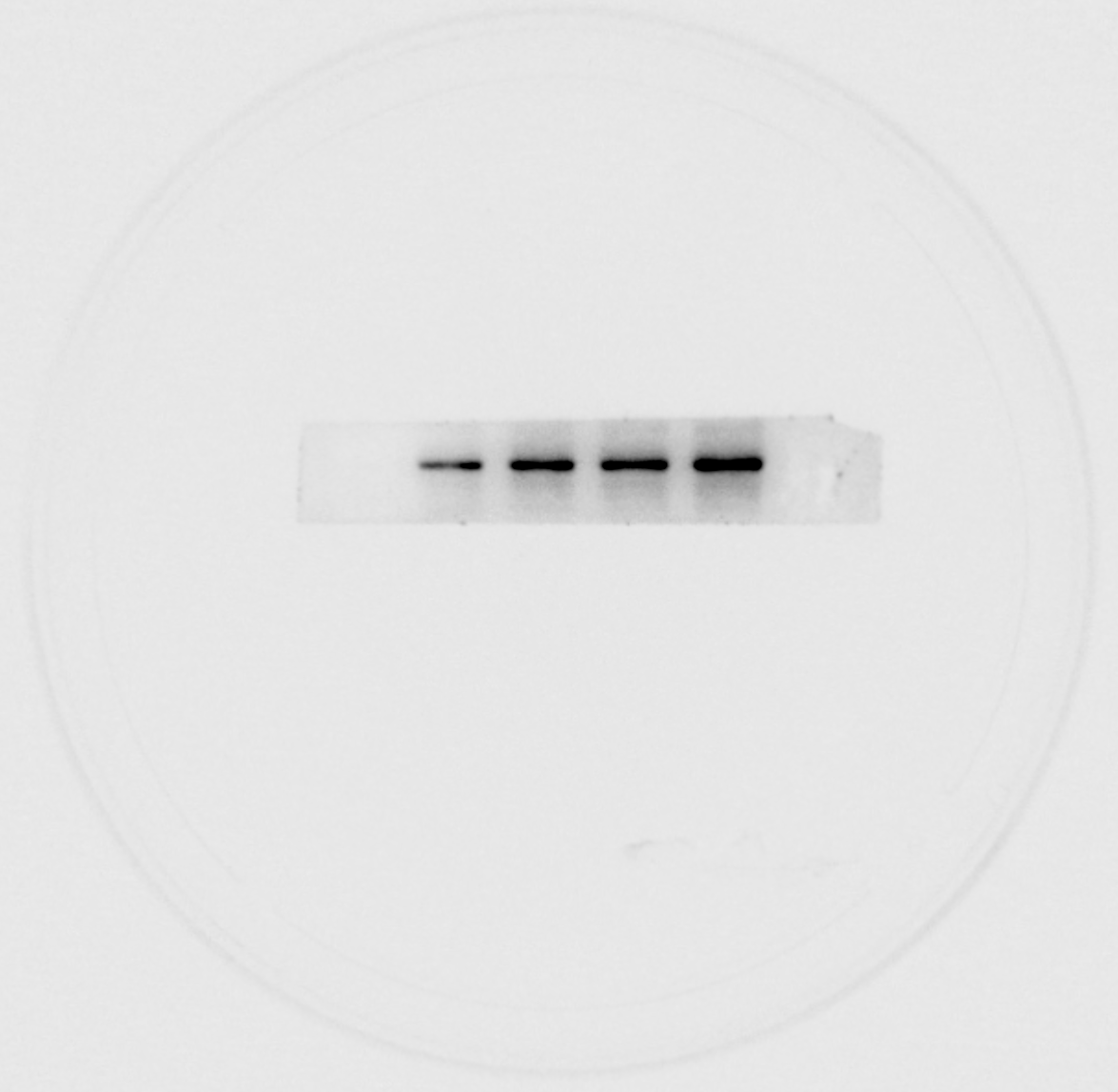

Supplement: Supplementary file 1 [file DataSheet1.ZIP › Oringinal western blots/HO-1/HO-1/HO-1 (2).tif]

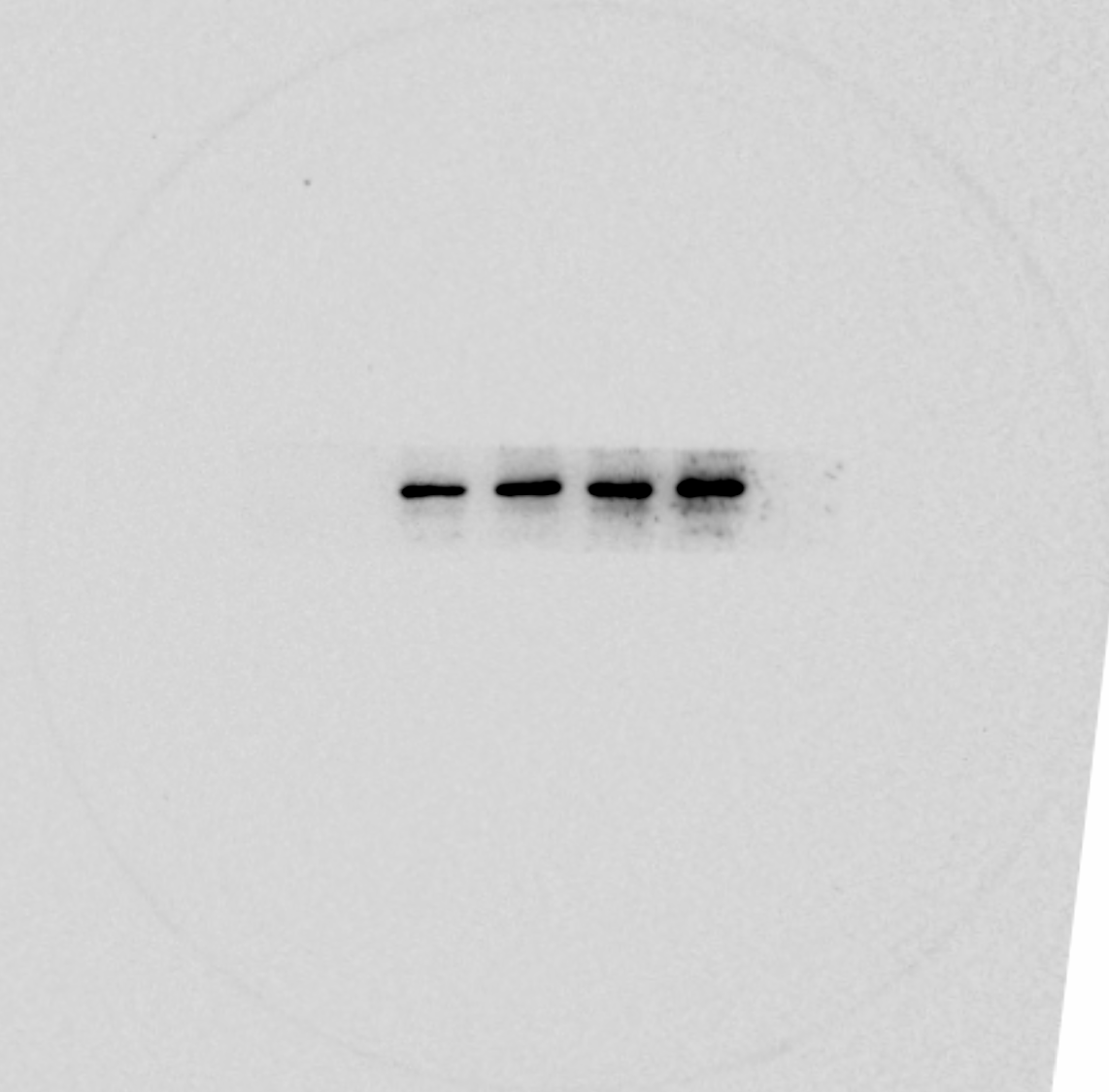

Supplement: Supplementary file 1 [file DataSheet1.ZIP › Oringinal western blots/HO-1/HO-1/HO-1 (3).tif]

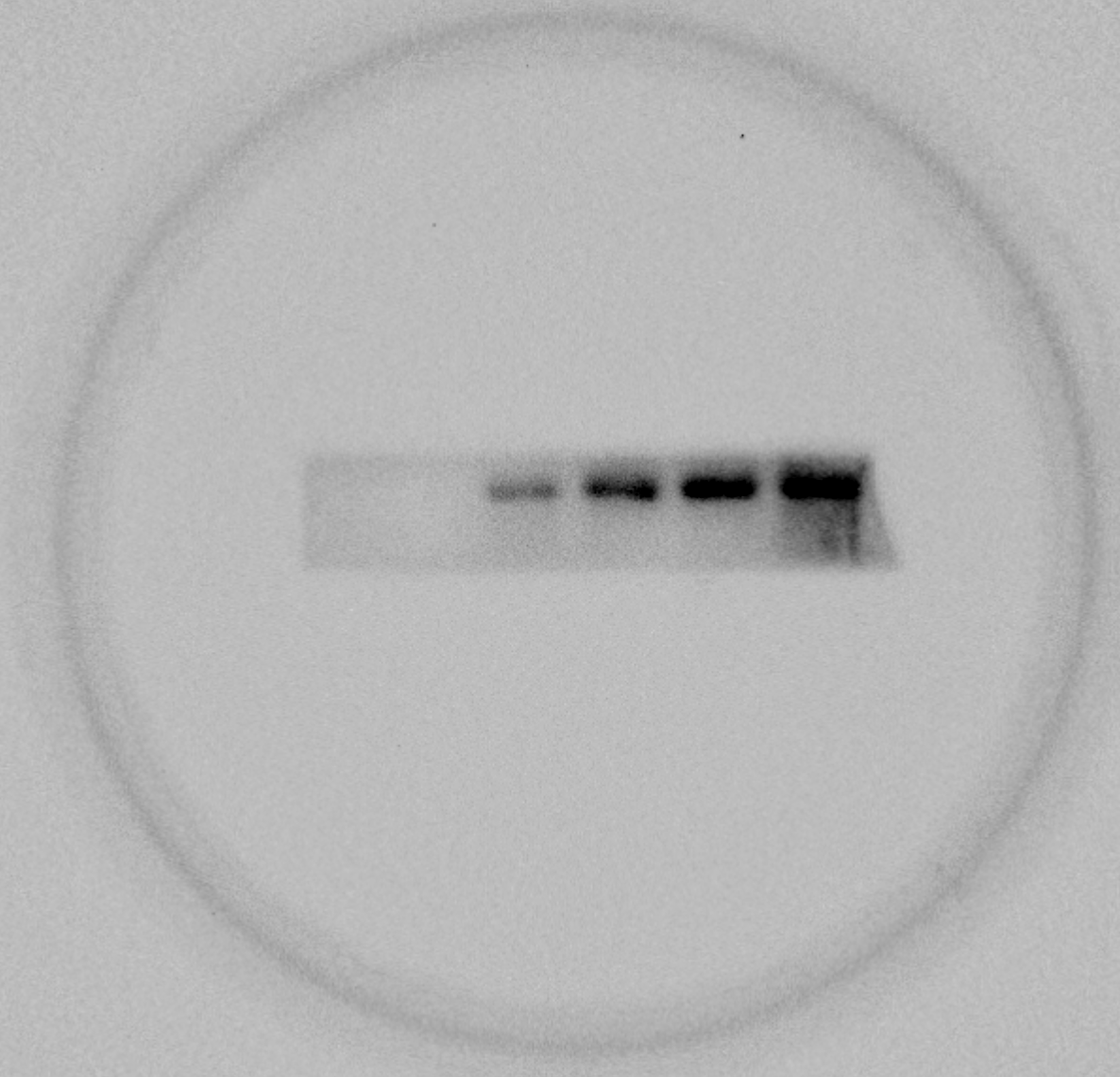

Supplement: Supplementary file 1 [file DataSheet1.ZIP › Oringinal western blots/HO-1/HO-1/HO-1 (4).tif]

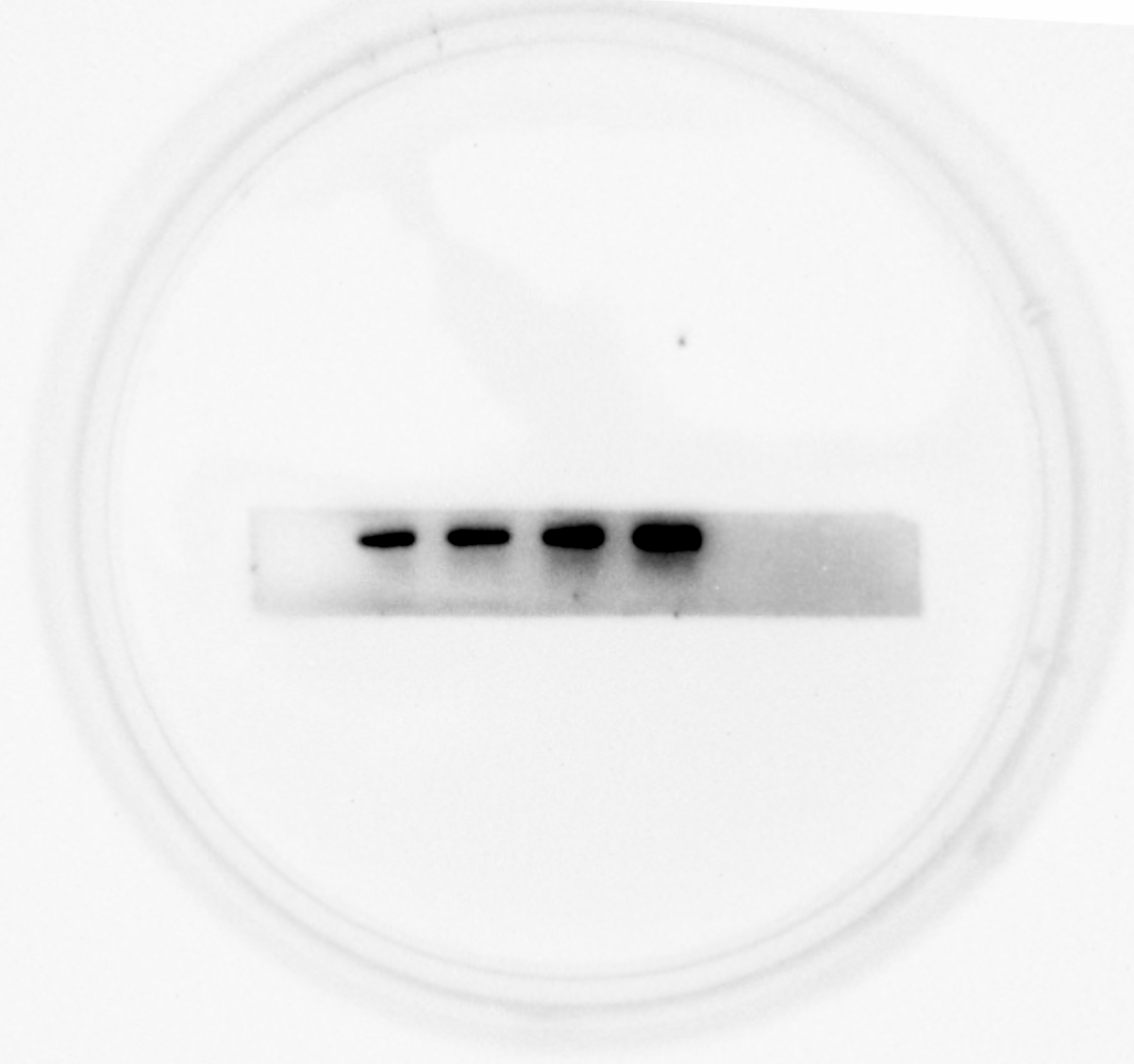

Supplement: Supplementary file 1 [file DataSheet1.ZIP › Oringinal western blots/HO-1/HO-1/HO-1 (5).tif]

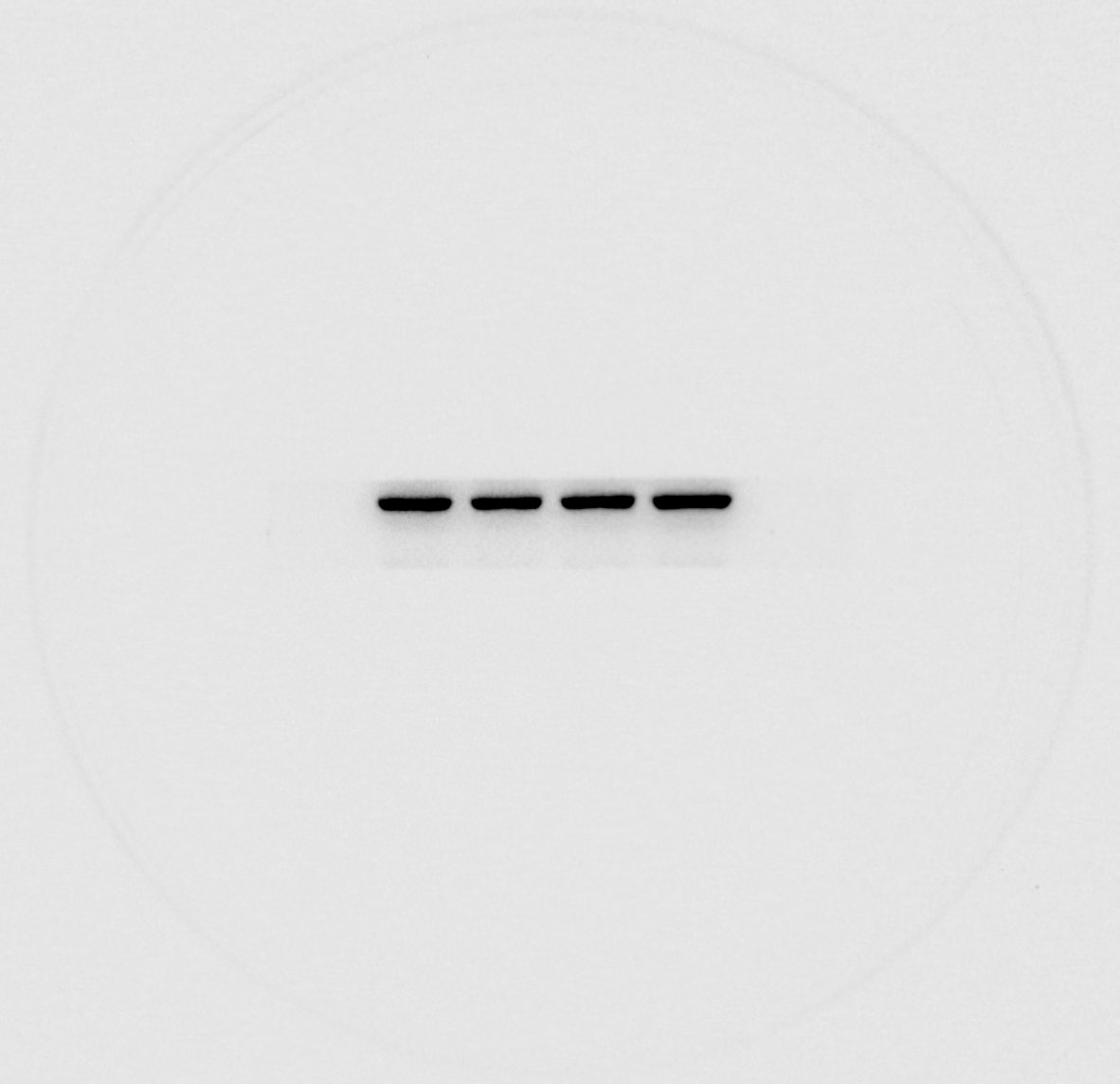

Supplement: Supplementary file 1 [file DataSheet1.ZIP › Oringinal western blots/HO-1/a┬-actin/a┬-actin (1).tif]

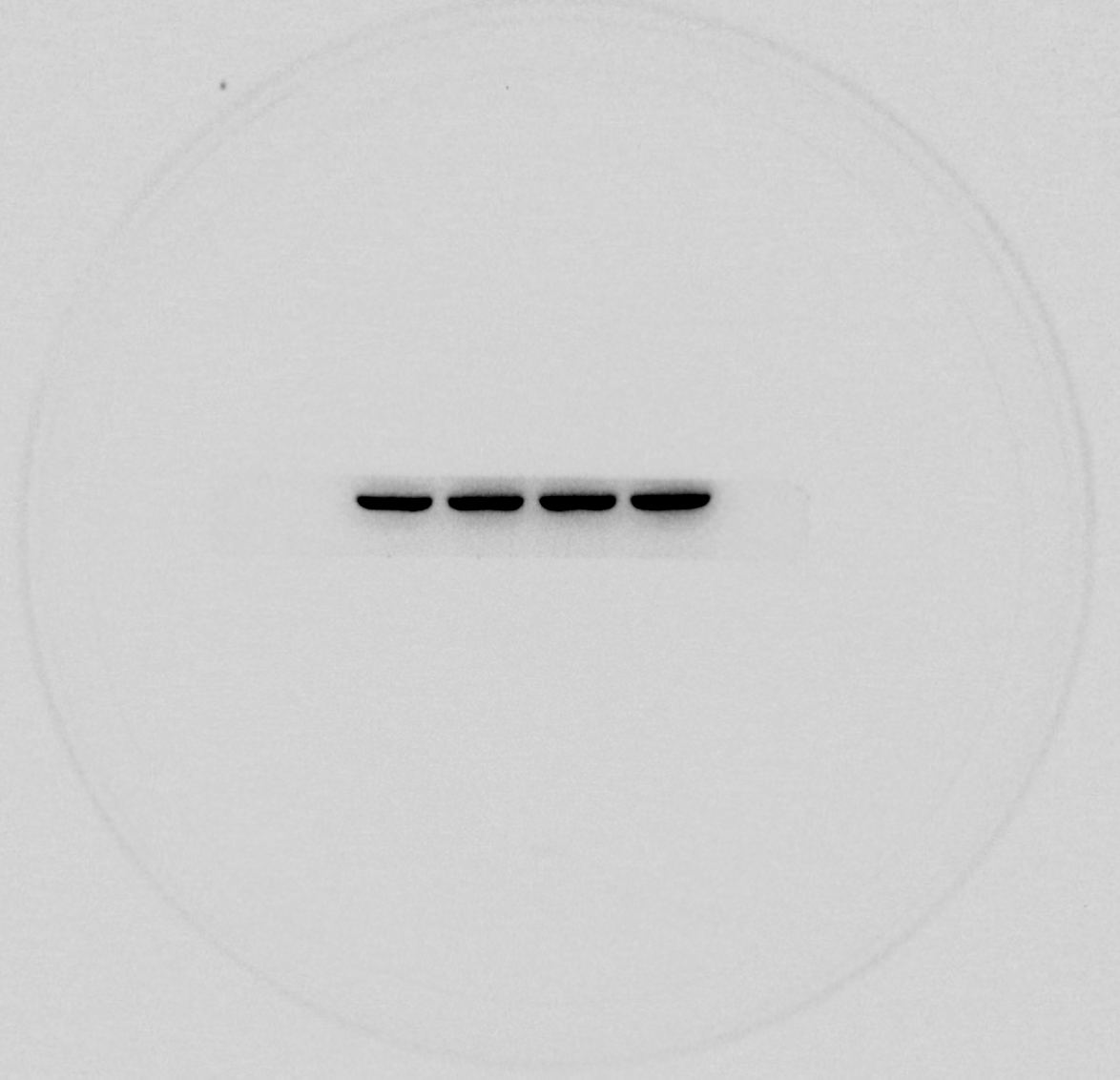

Supplement: Supplementary file 1 [file DataSheet1.ZIP › Oringinal western blots/HO-1/a┬-actin/a┬-actin (2).tif]

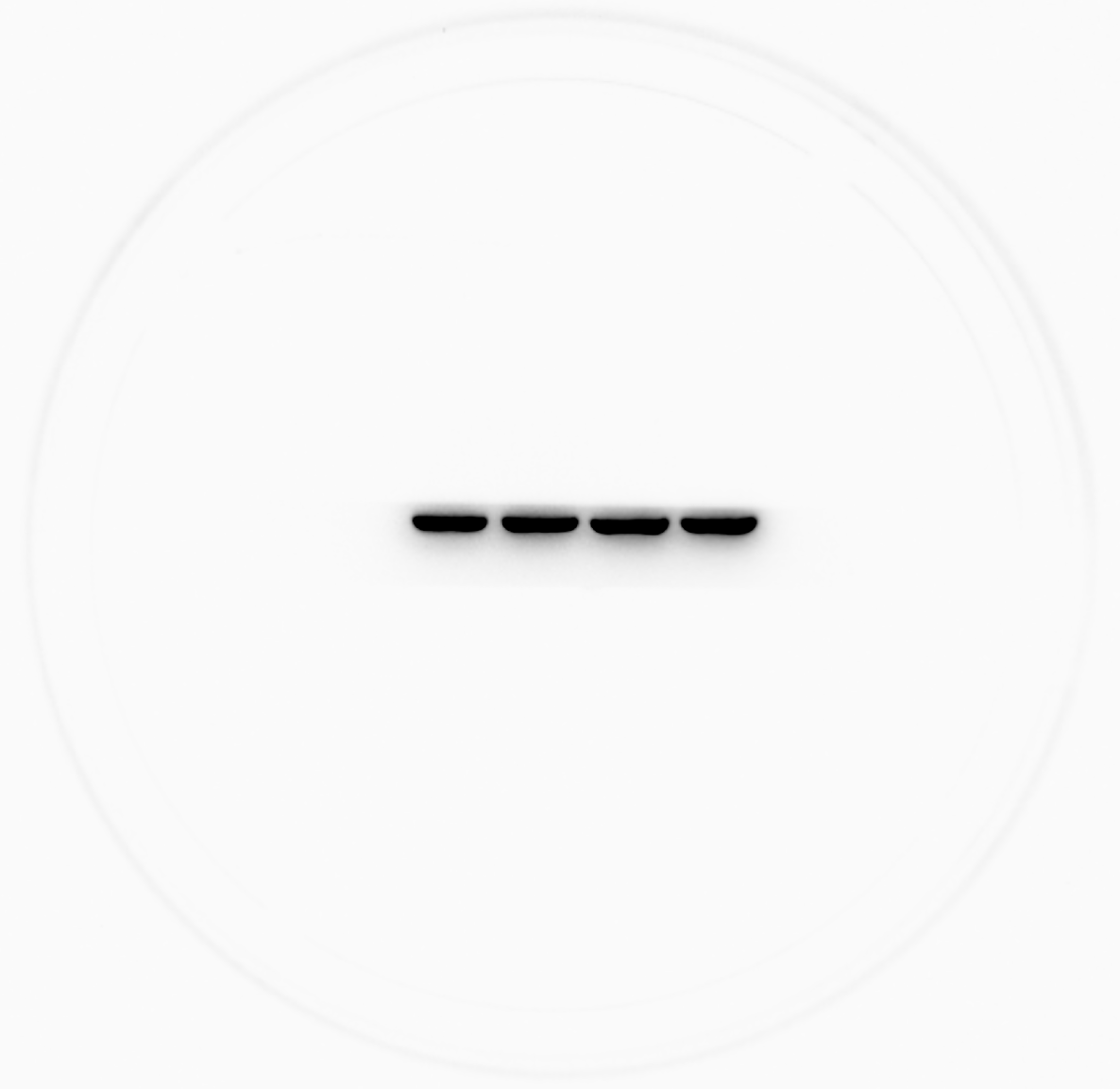

Supplement: Supplementary file 1 [file DataSheet1.ZIP › Oringinal western blots/HO-1/a┬-actin/a┬-actin (3).tif]

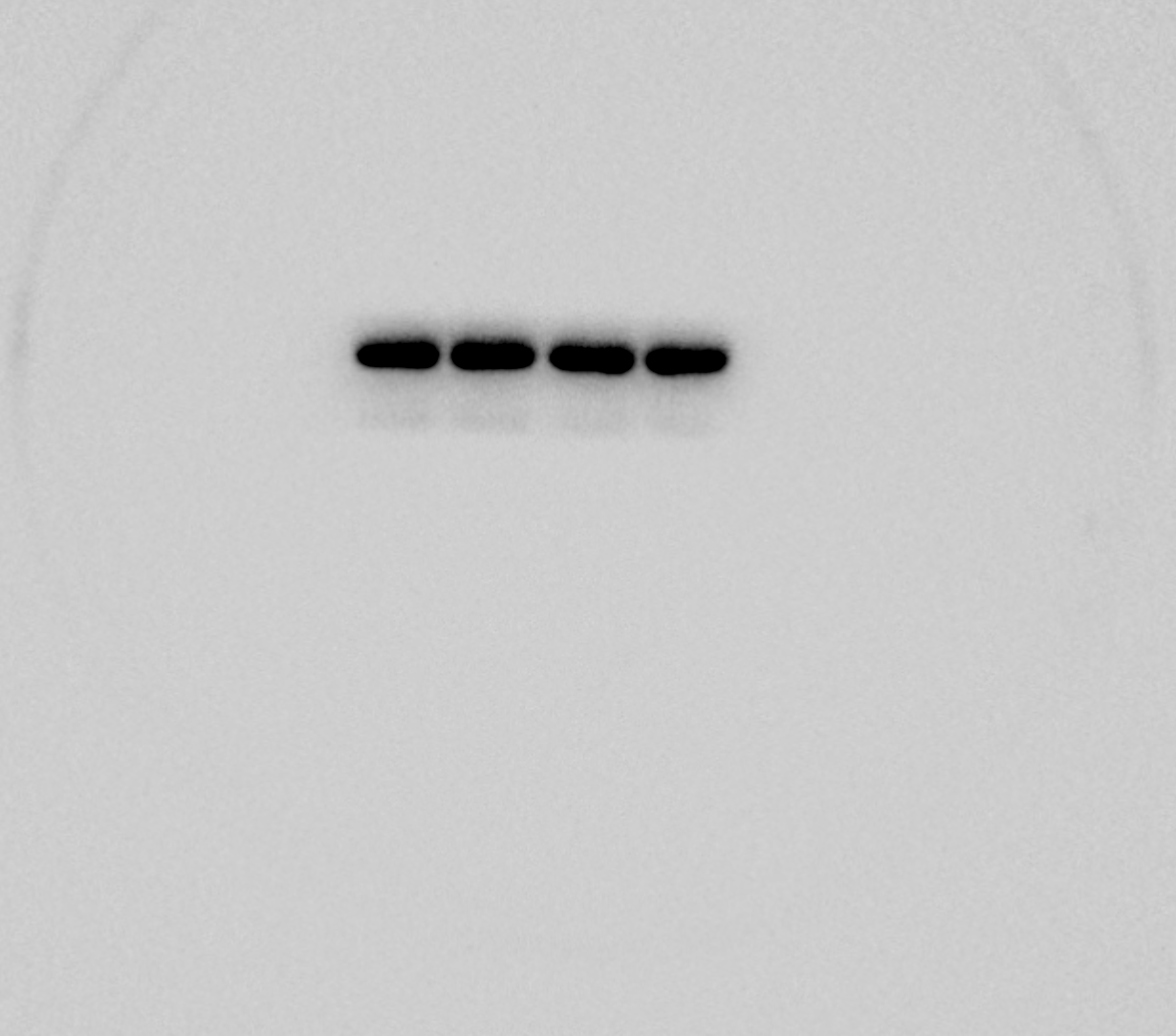

Supplement: Supplementary file 1 [file DataSheet1.ZIP › Oringinal western blots/HO-1/a┬-actin/a┬-actin (4).tif]

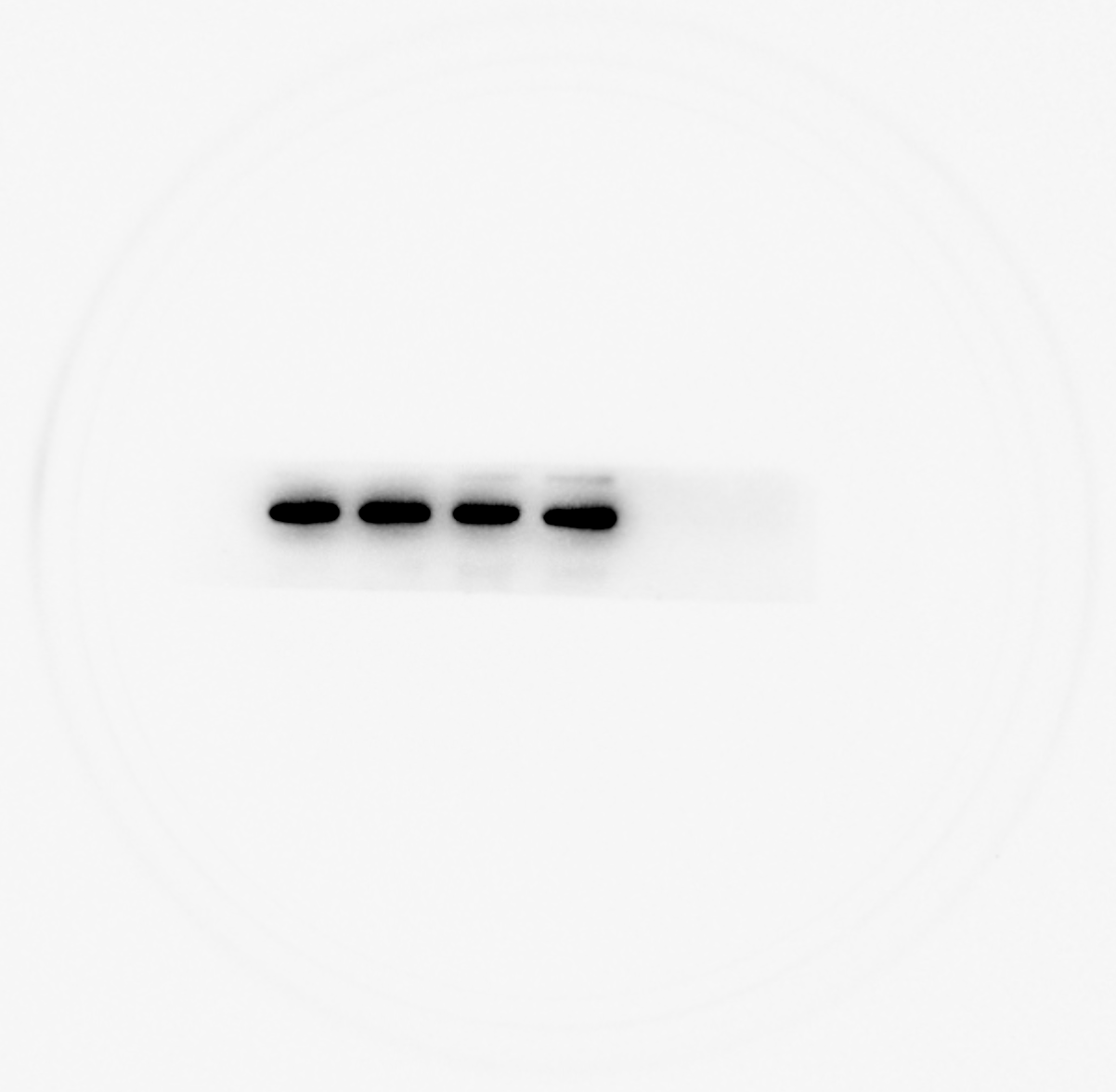

Supplement: Supplementary file 1 [file DataSheet1.ZIP › Oringinal western blots/HO-1/a┬-actin/a┬-actin (5).tif]

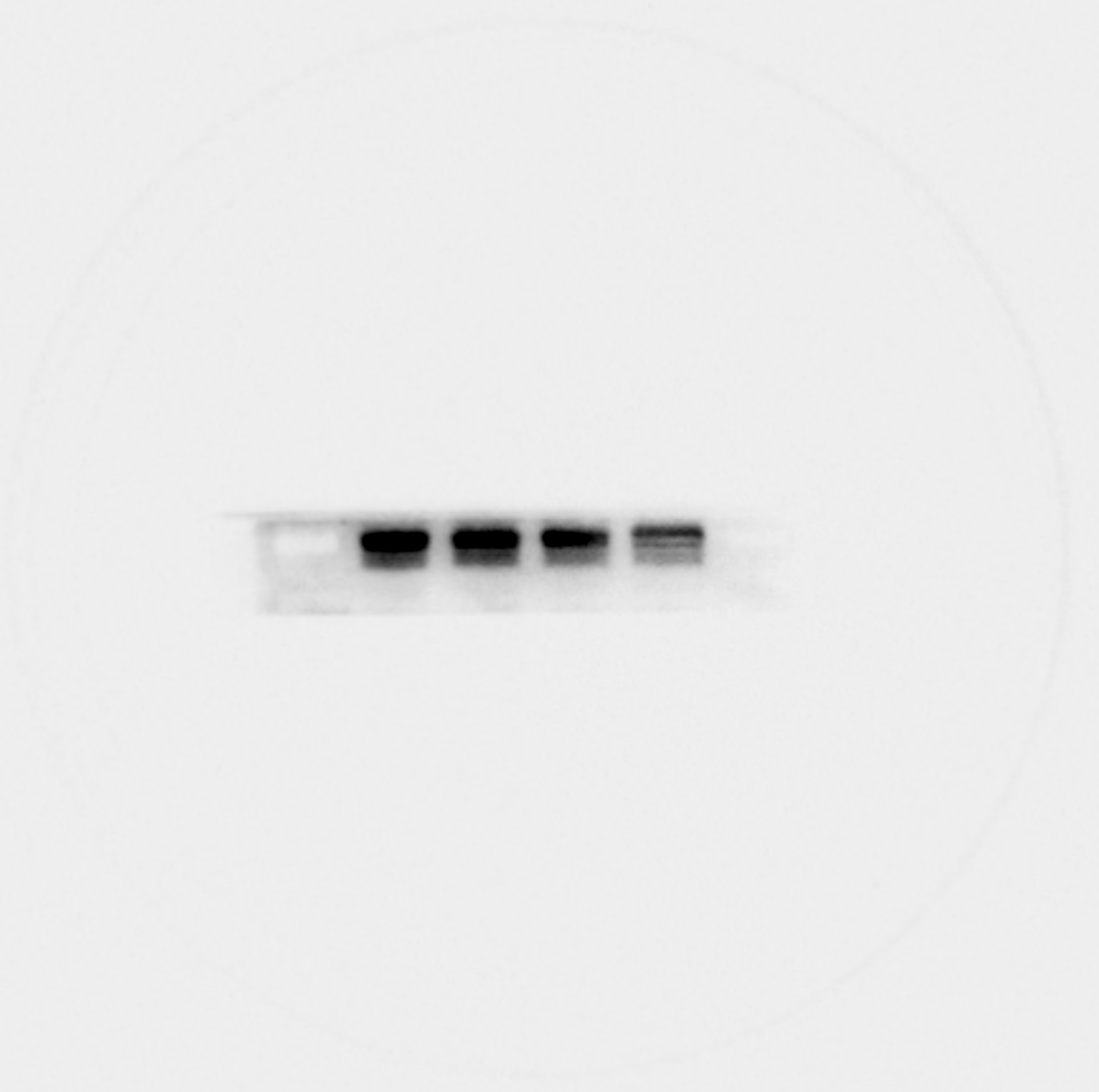

Supplement: Supplementary file 1 [file DataSheet1.ZIP › Oringinal western blots/Keap1/Keap1/keap1 (1).tif]

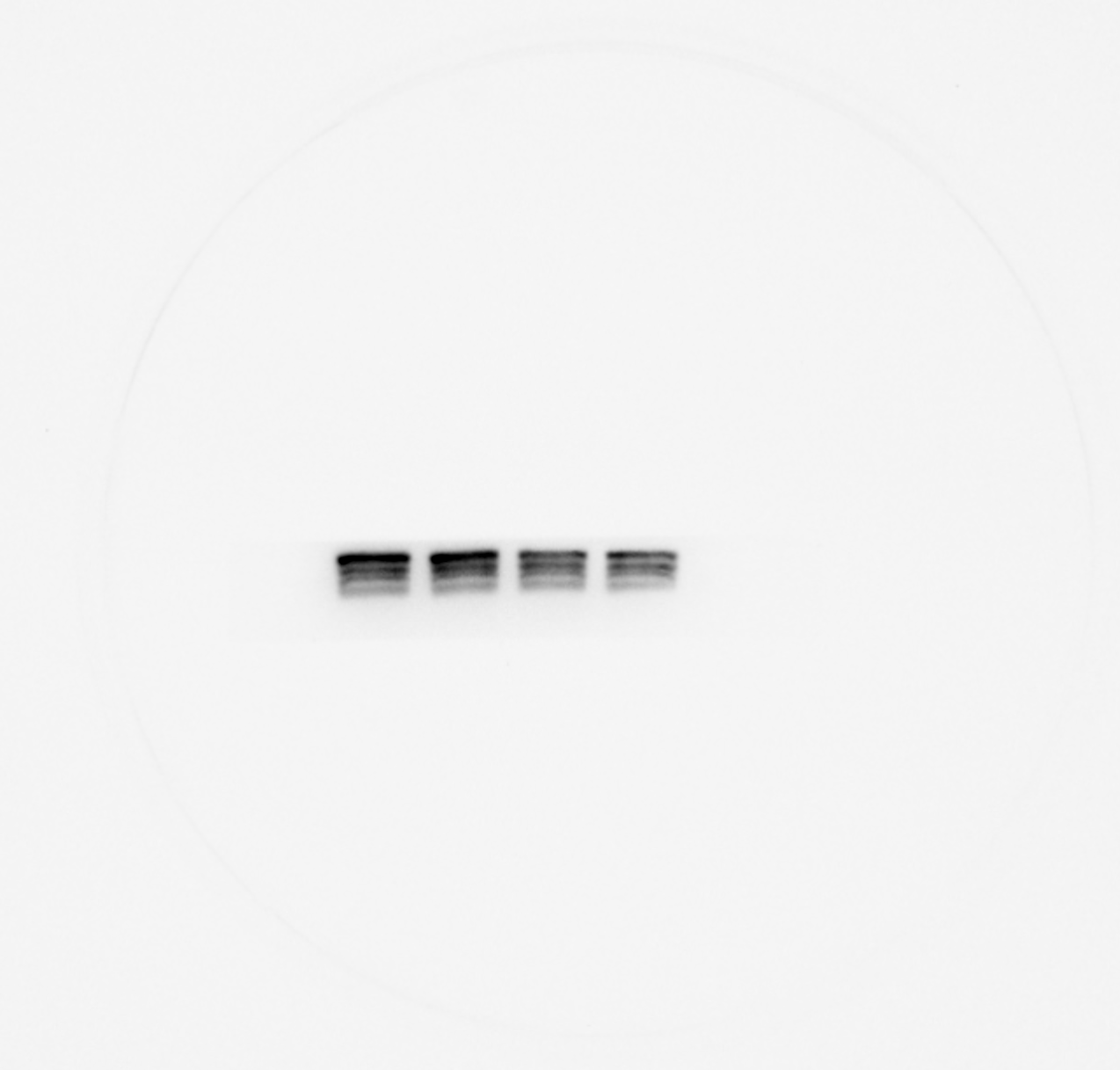

Supplement: Supplementary file 1 [file DataSheet1.ZIP › Oringinal western blots/Keap1/Keap1/keap1 (2).tif]

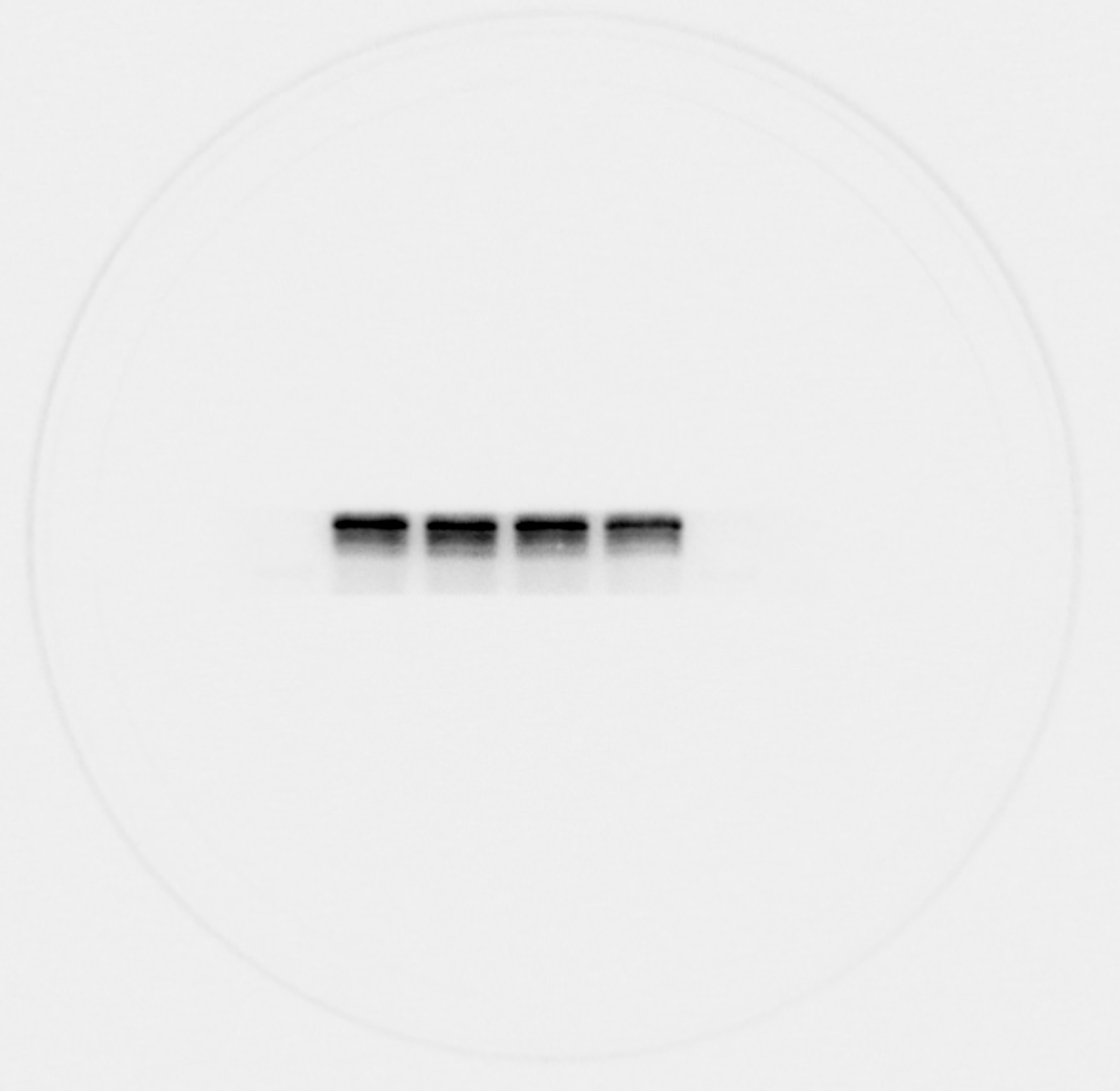

Supplement: Supplementary file 1 [file DataSheet1.ZIP › Oringinal western blots/Keap1/Keap1/keap1(3).tif]

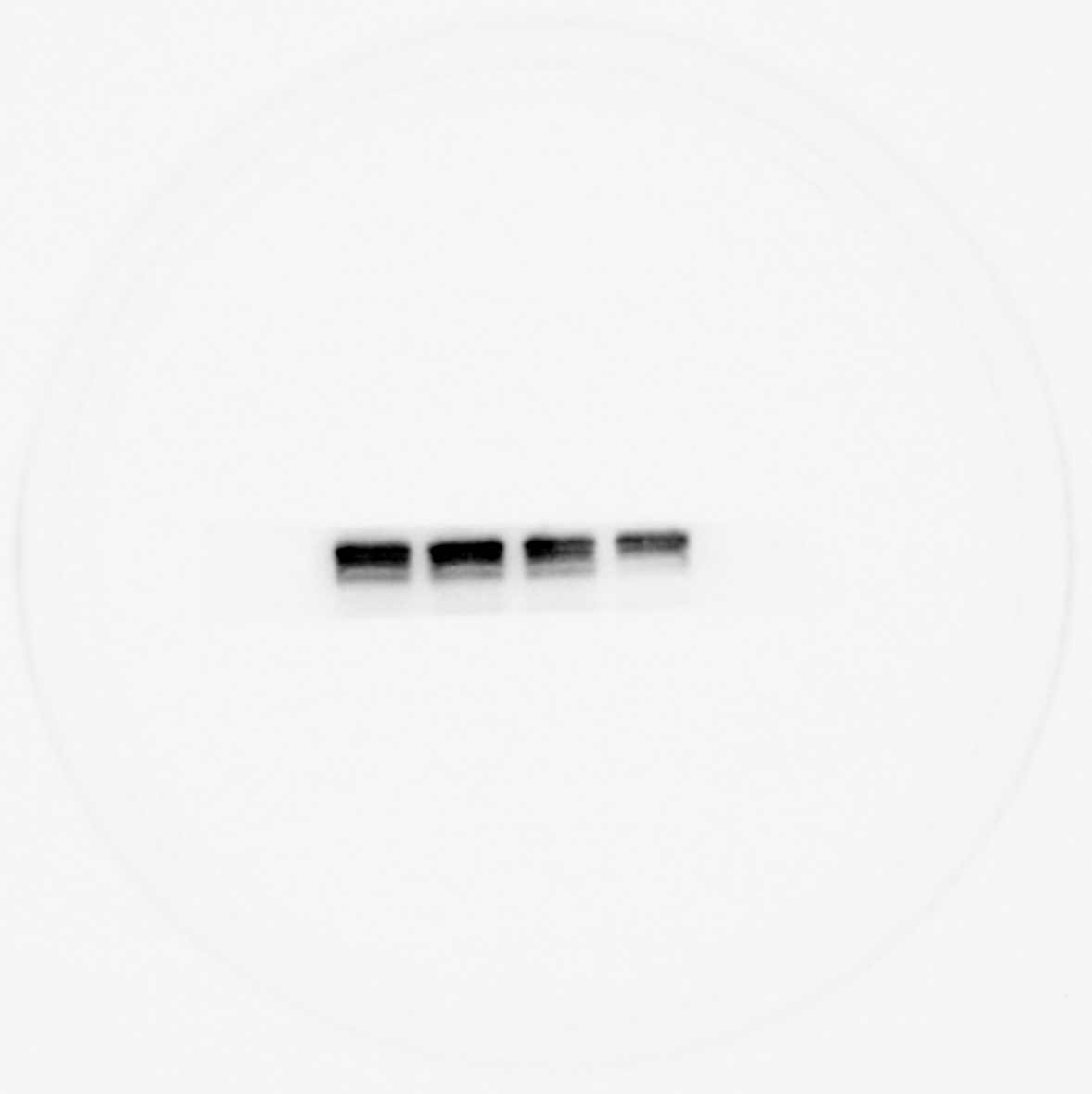

Supplement: Supplementary file 1 [file DataSheet1.ZIP › Oringinal western blots/Keap1/Keap1/keap1(4).tif]

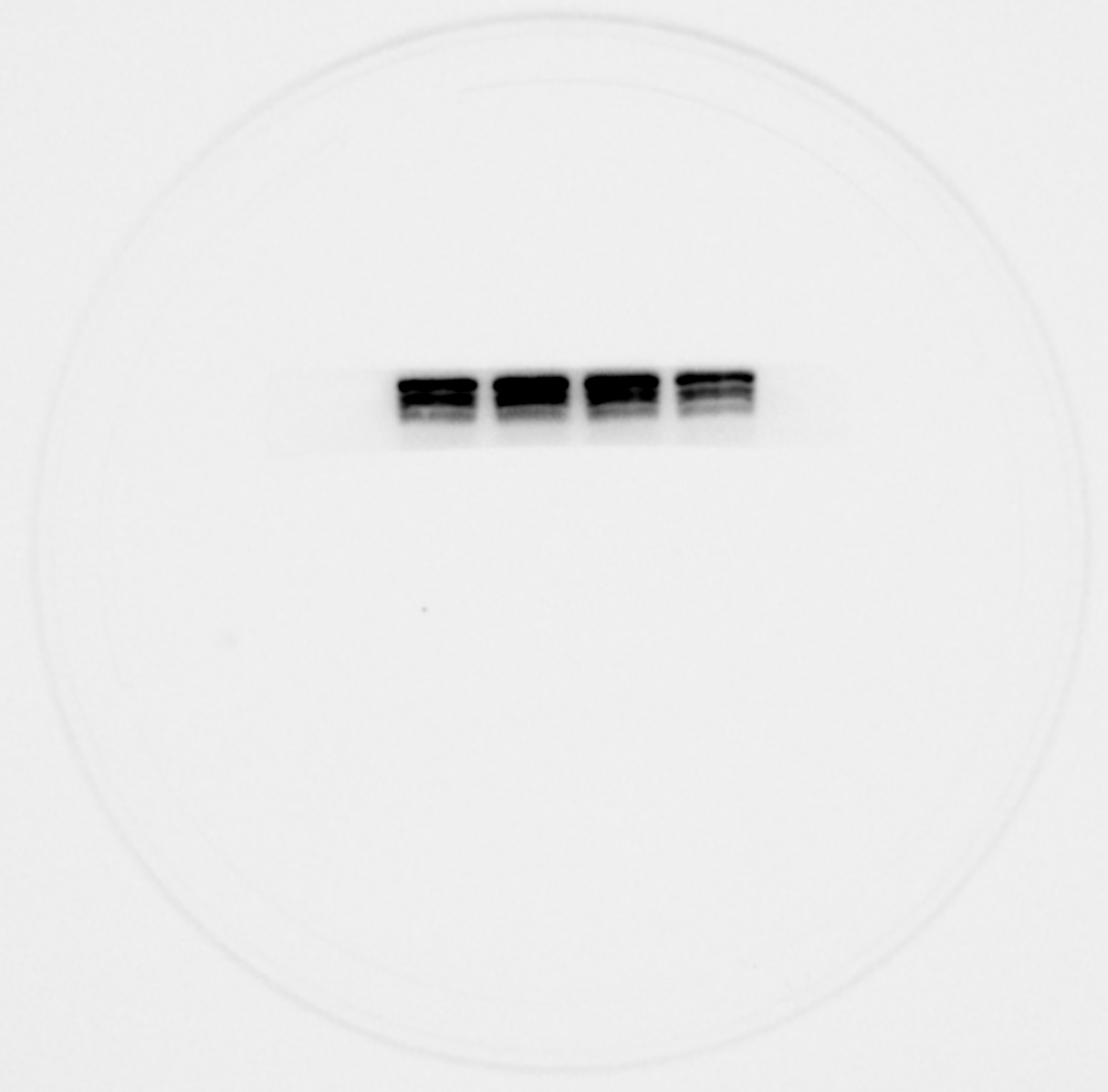

Supplement: Supplementary file 1 [file DataSheet1.ZIP › Oringinal western blots/Keap1/Keap1/keap1(5).tif]

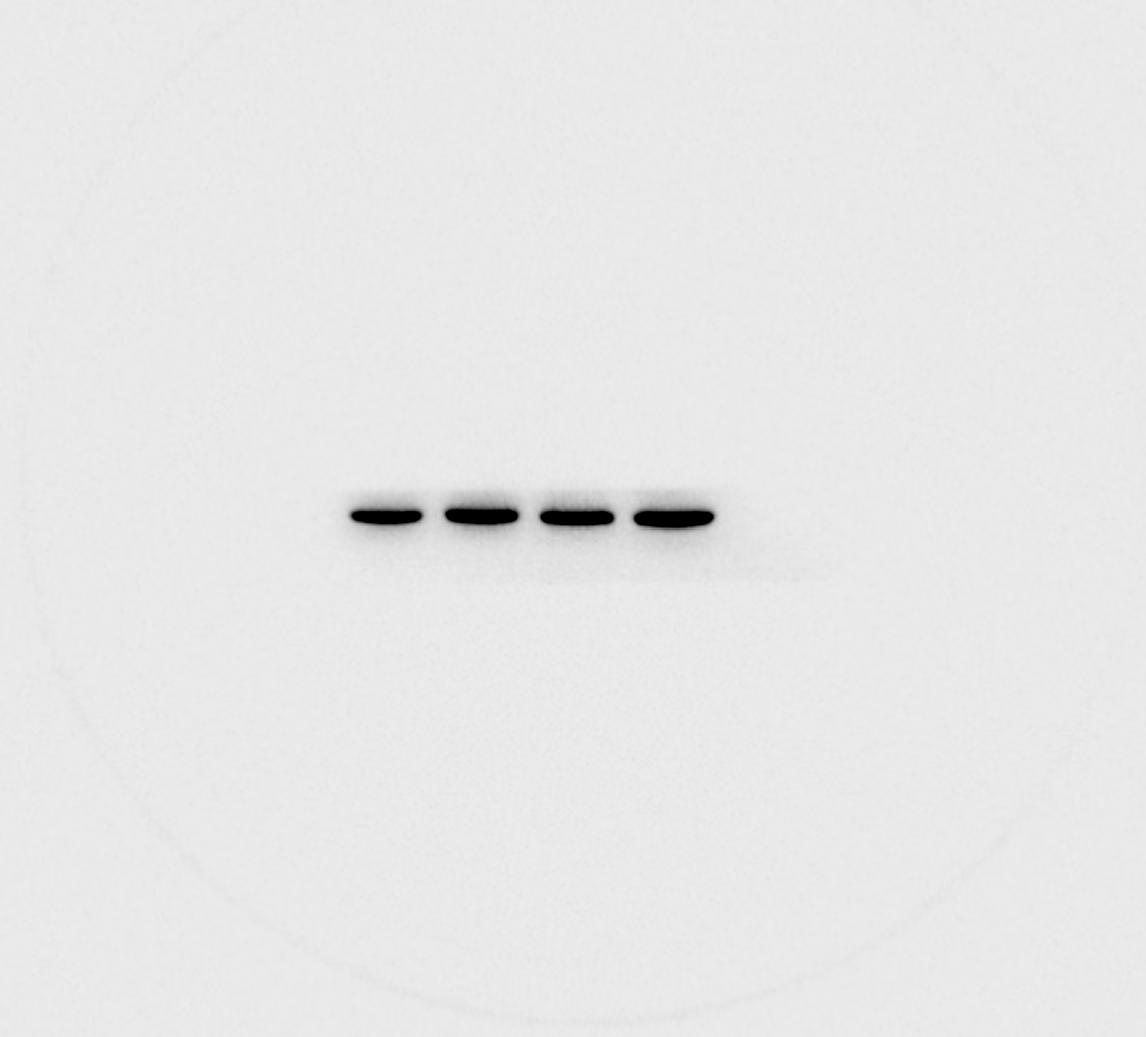

Supplement: Supplementary file 1 [file DataSheet1.ZIP › Oringinal western blots/Keap1/a┬-actin/a┬-actin (1).tif]

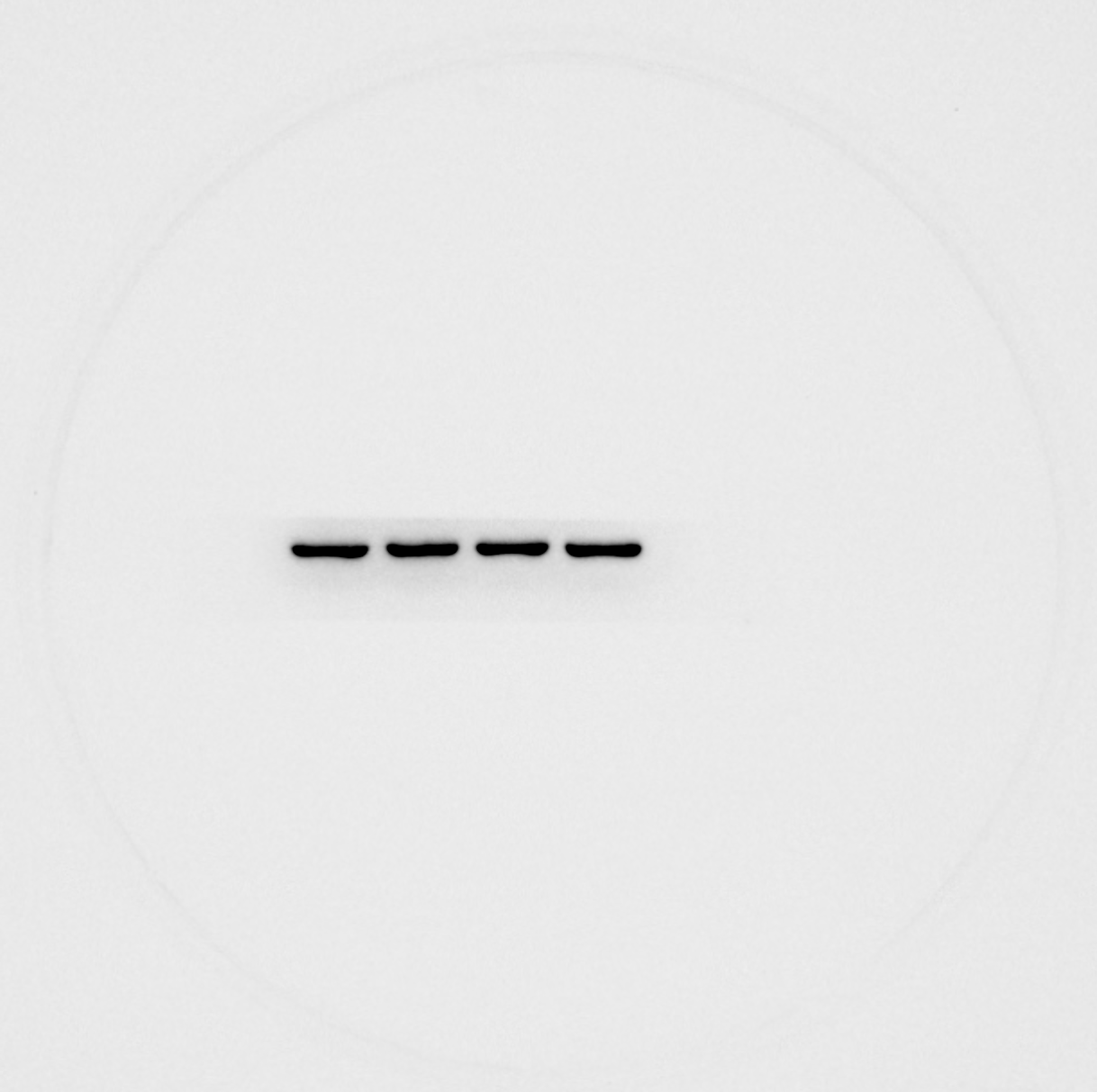

Supplement: Supplementary file 1 [file DataSheet1.ZIP › Oringinal western blots/Keap1/a┬-actin/a┬-actin (2).tif]

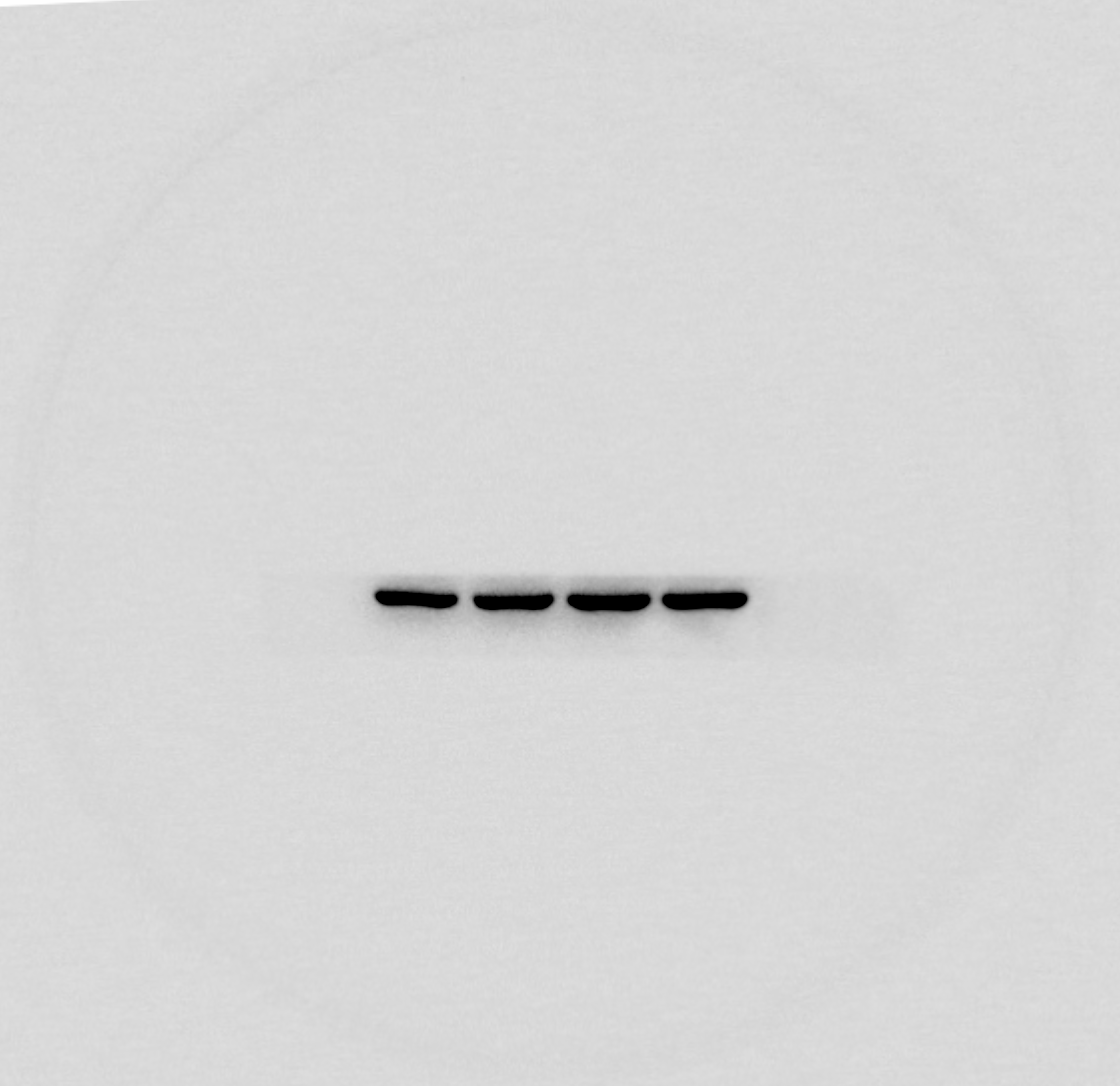

Supplement: Supplementary file 1 [file DataSheet1.ZIP › Oringinal western blots/Keap1/a┬-actin/a┬-actin (3).tif]

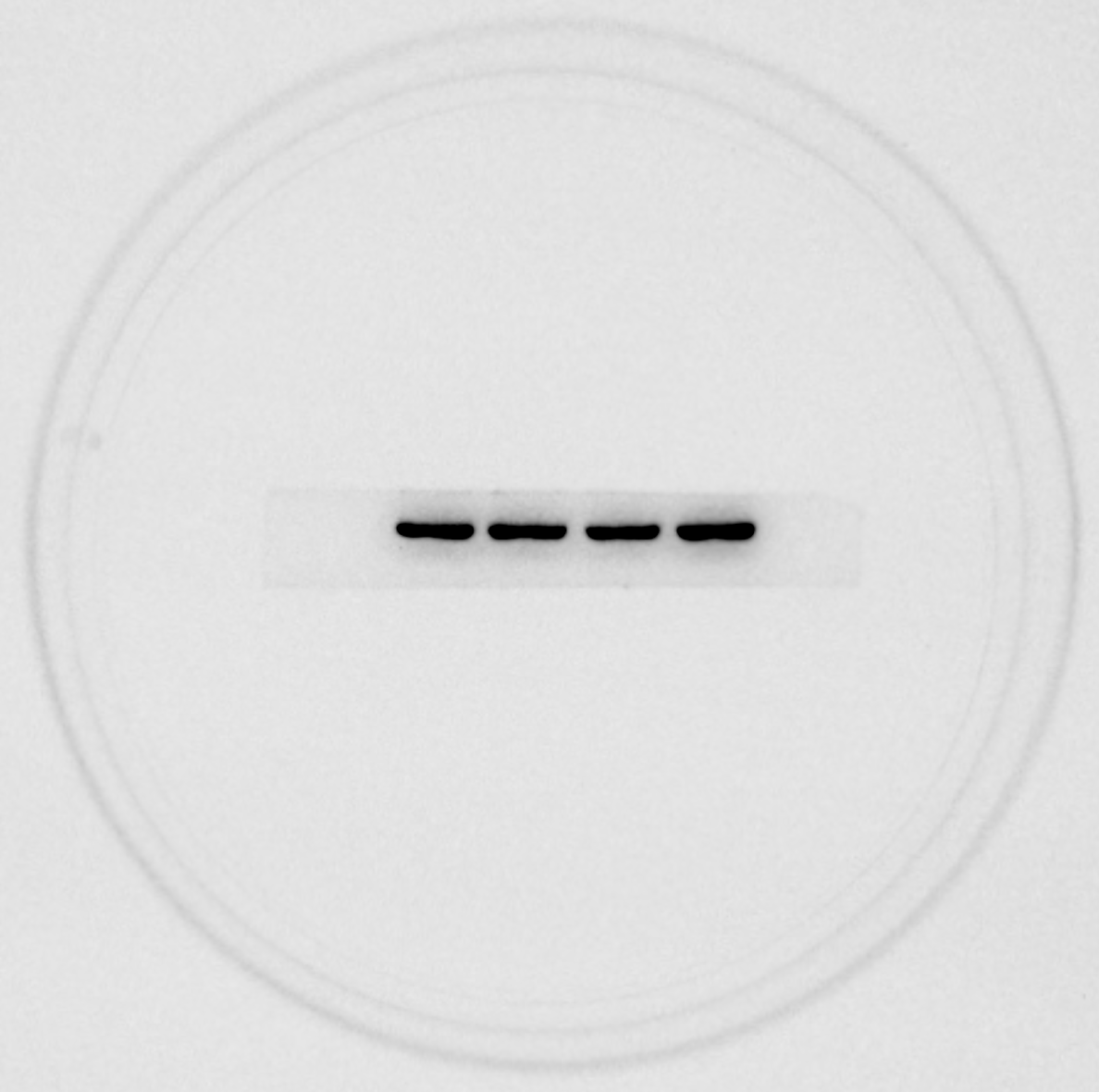

Supplement: Supplementary file 1 [file DataSheet1.ZIP › Oringinal western blots/Keap1/a┬-actin/a┬-actin (4).tif]

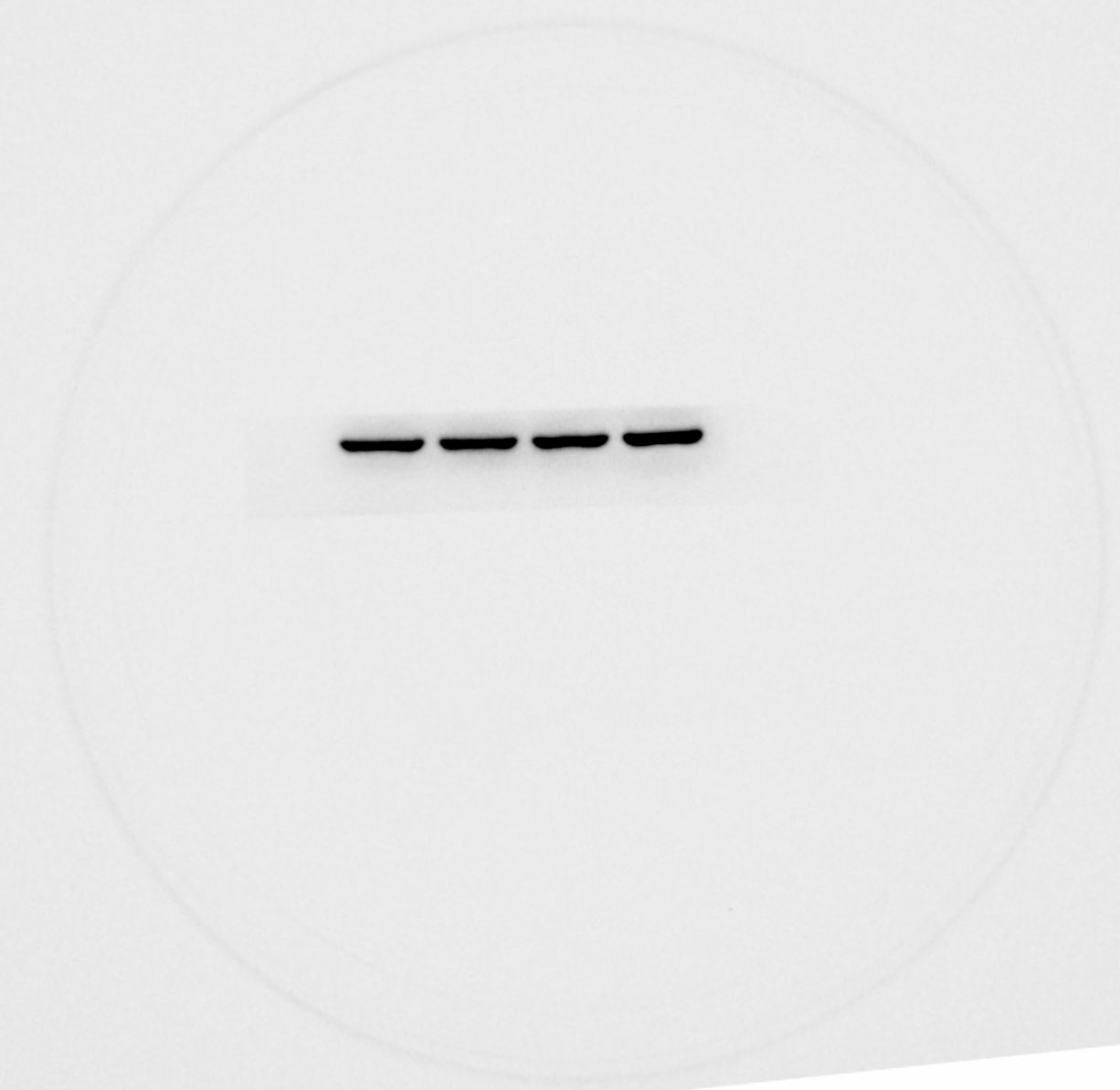

Supplement: Supplementary file 1 [file DataSheet1.ZIP › Oringinal western blots/Keap1/a┬-actin/a┬-actin (5).tif]

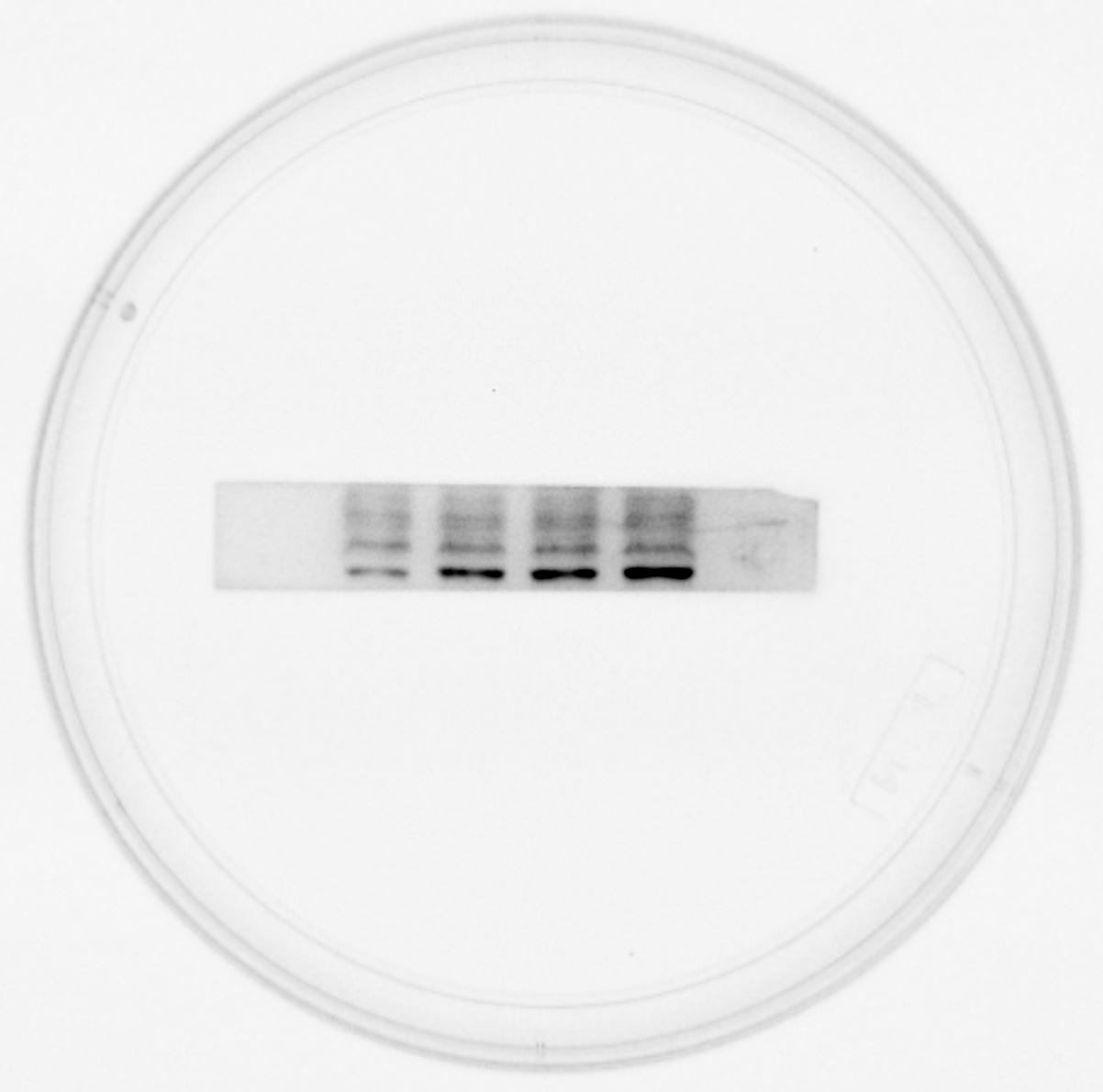

Supplement: Supplementary file 1 [file DataSheet1.ZIP › Oringinal western blots/NQO-1/NQO1/NQO1 (1).tif]

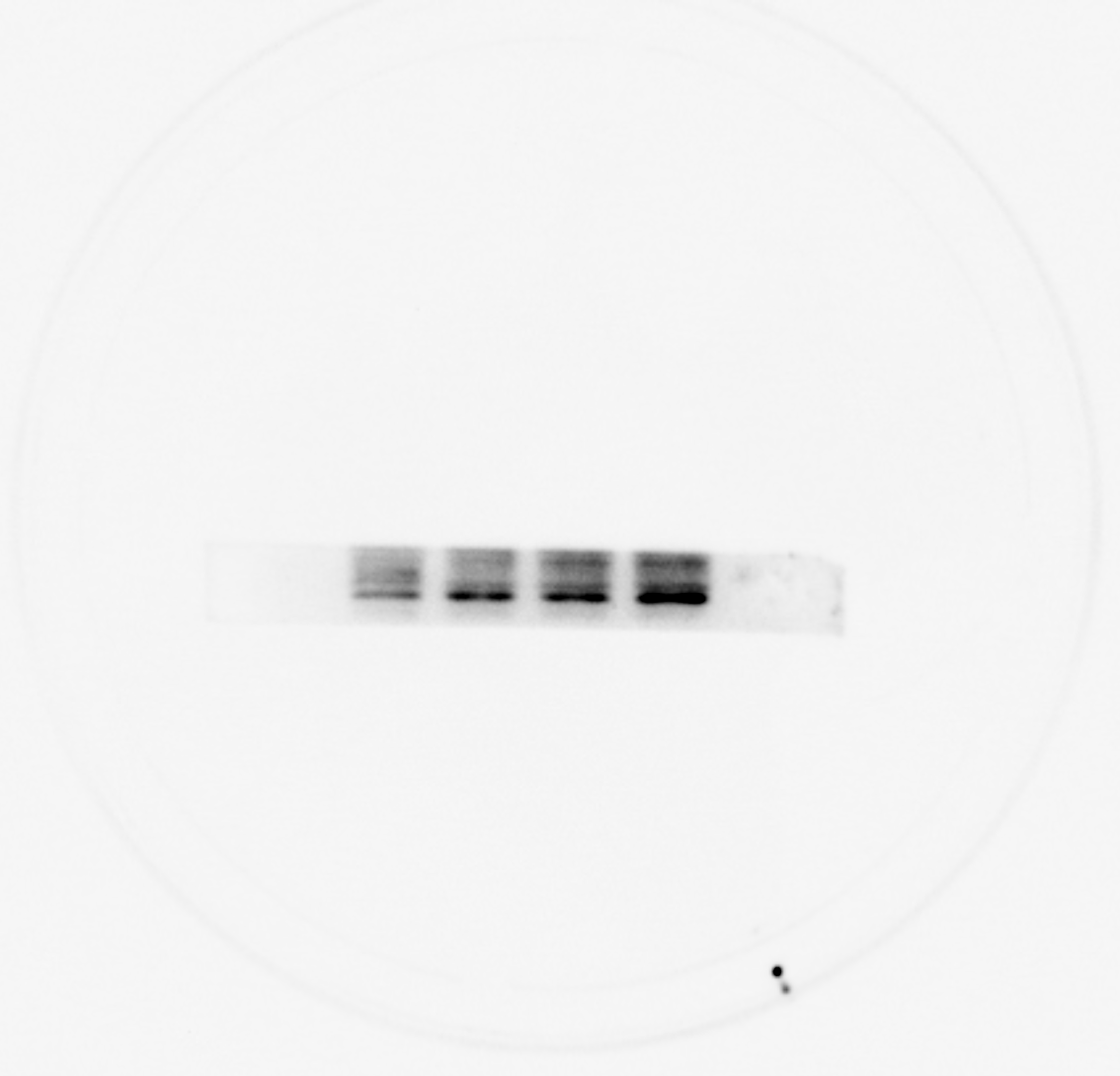

Supplement: Supplementary file 1 [file DataSheet1.ZIP › Oringinal western blots/NQO-1/NQO1/NQO1 (2).tif]

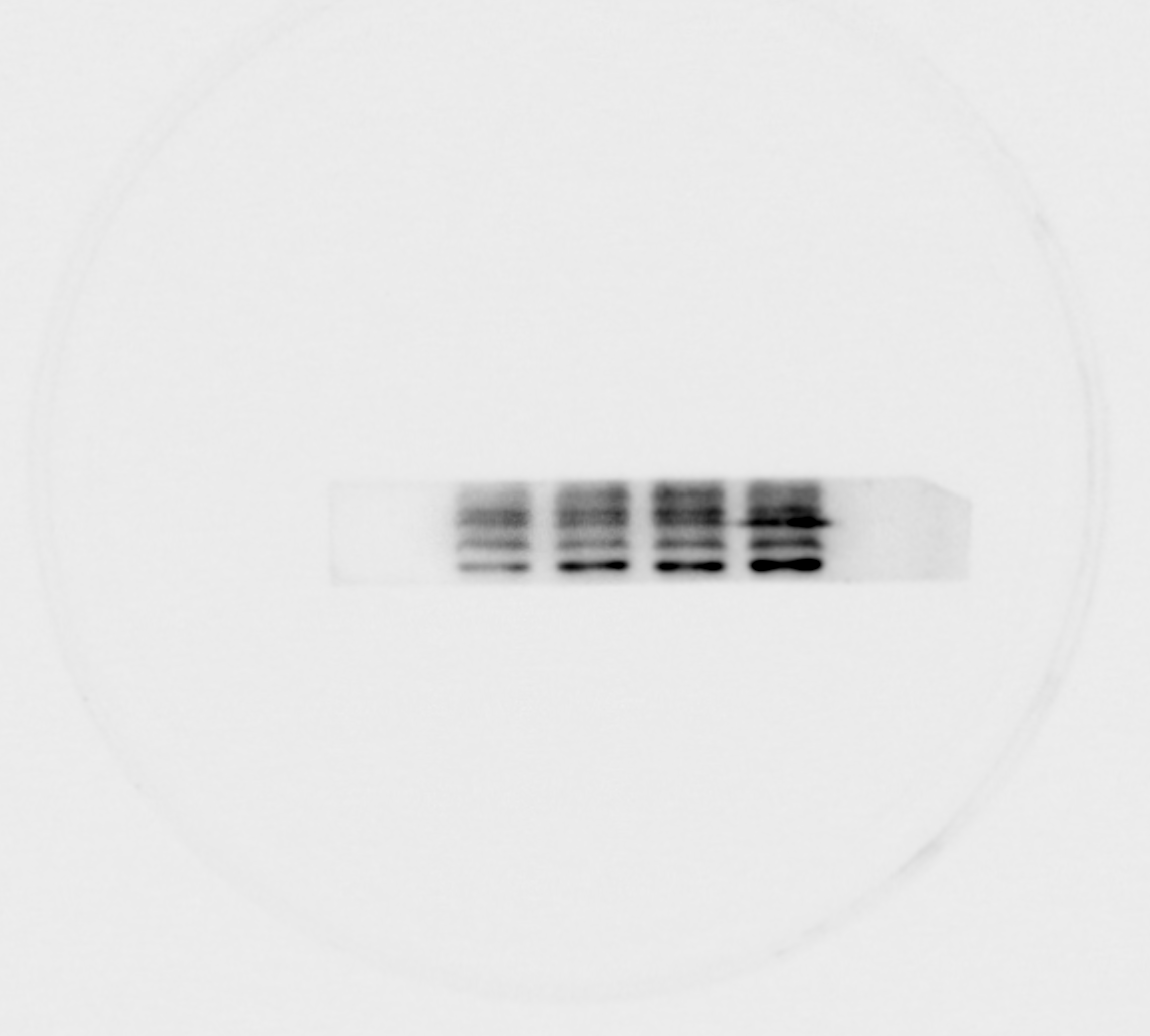

Supplement: Supplementary file 1 [file DataSheet1.ZIP › Oringinal western blots/NQO-1/NQO1/NQO1 (3).tif]

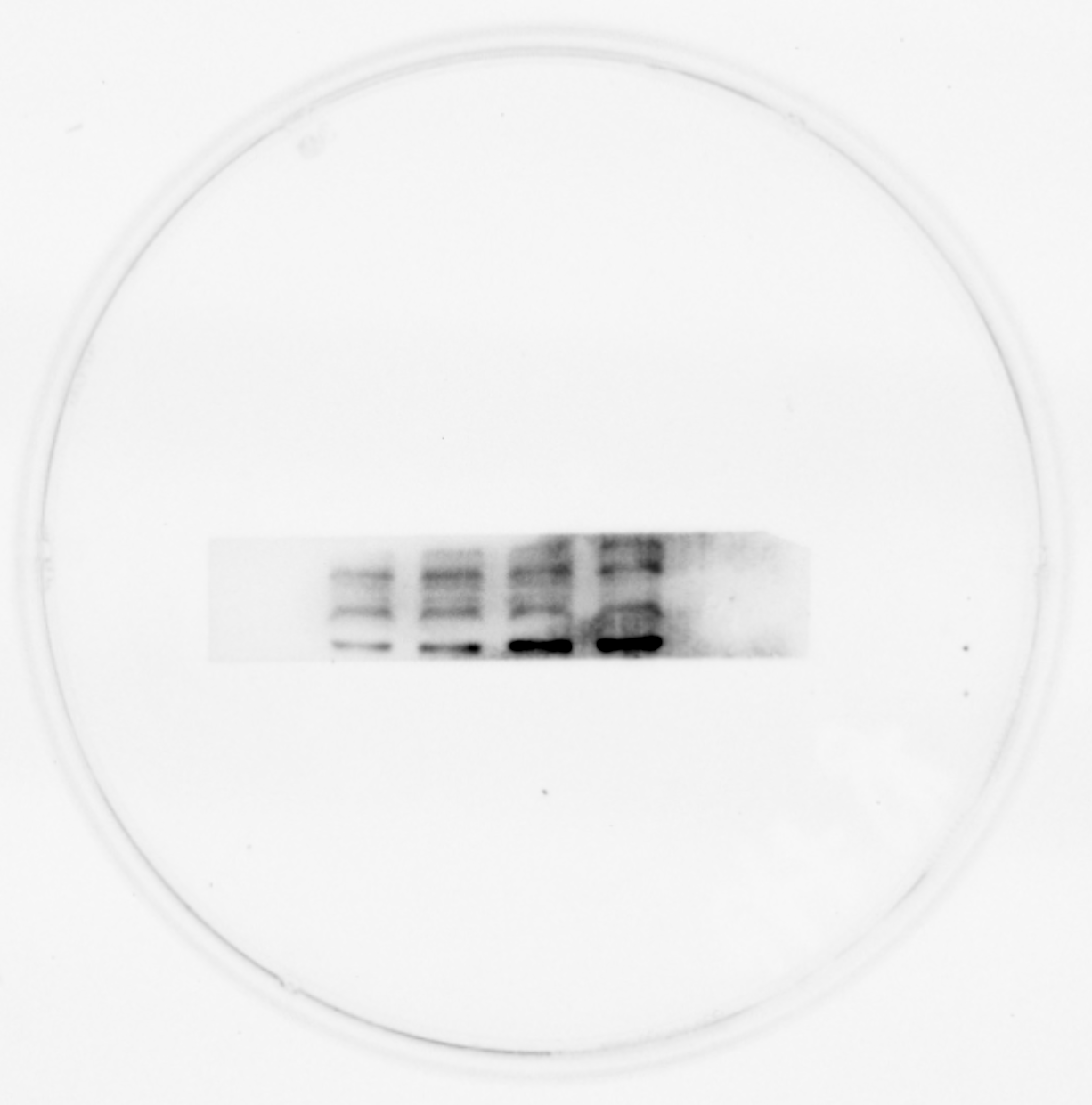

Supplement: Supplementary file 1 [file DataSheet1.ZIP › Oringinal western blots/NQO-1/NQO1/NQO1 (4).tif]

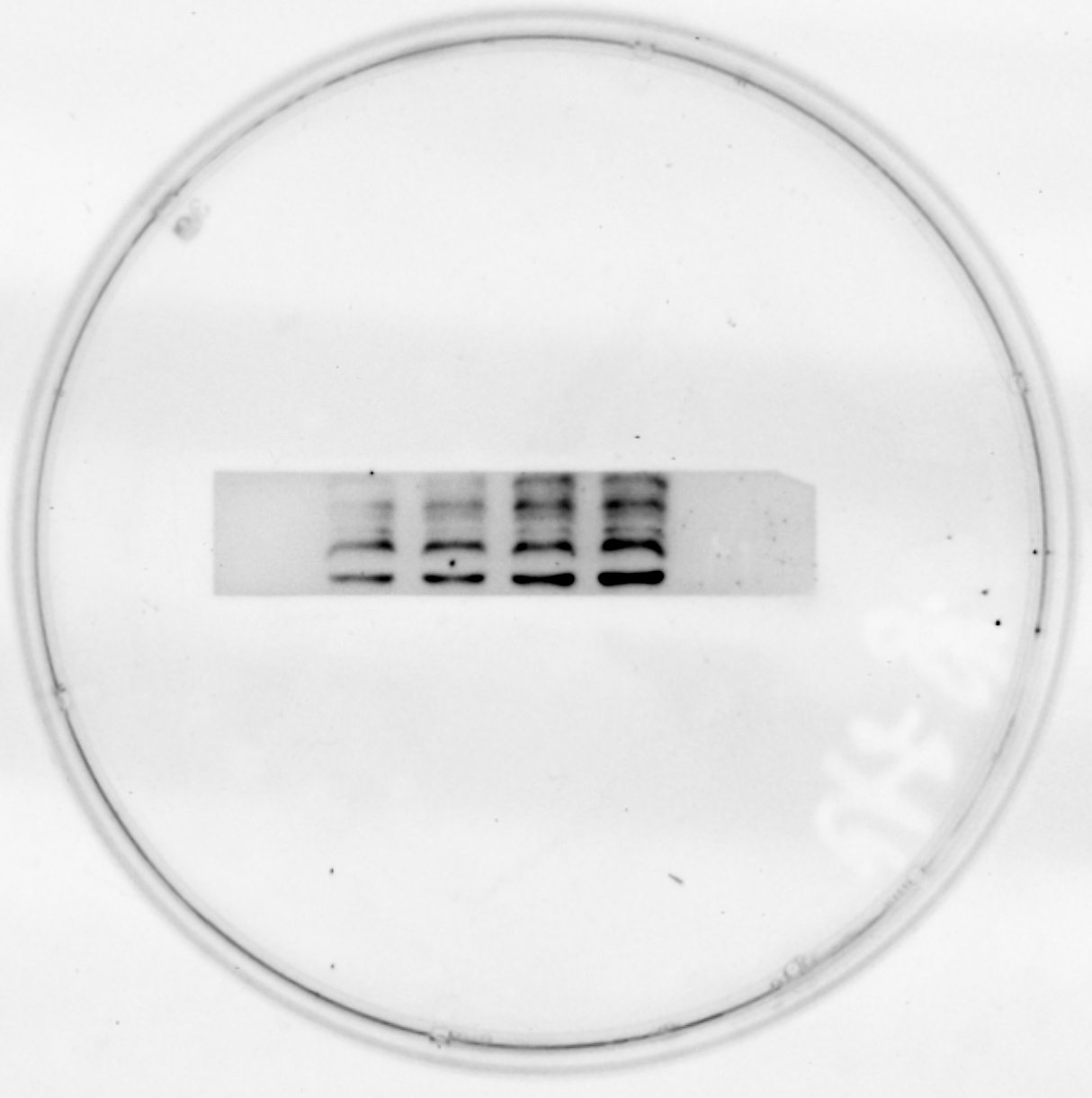

Supplement: Supplementary file 1 [file DataSheet1.ZIP › Oringinal western blots/NQO-1/NQO1/NQO1 (5).tif]

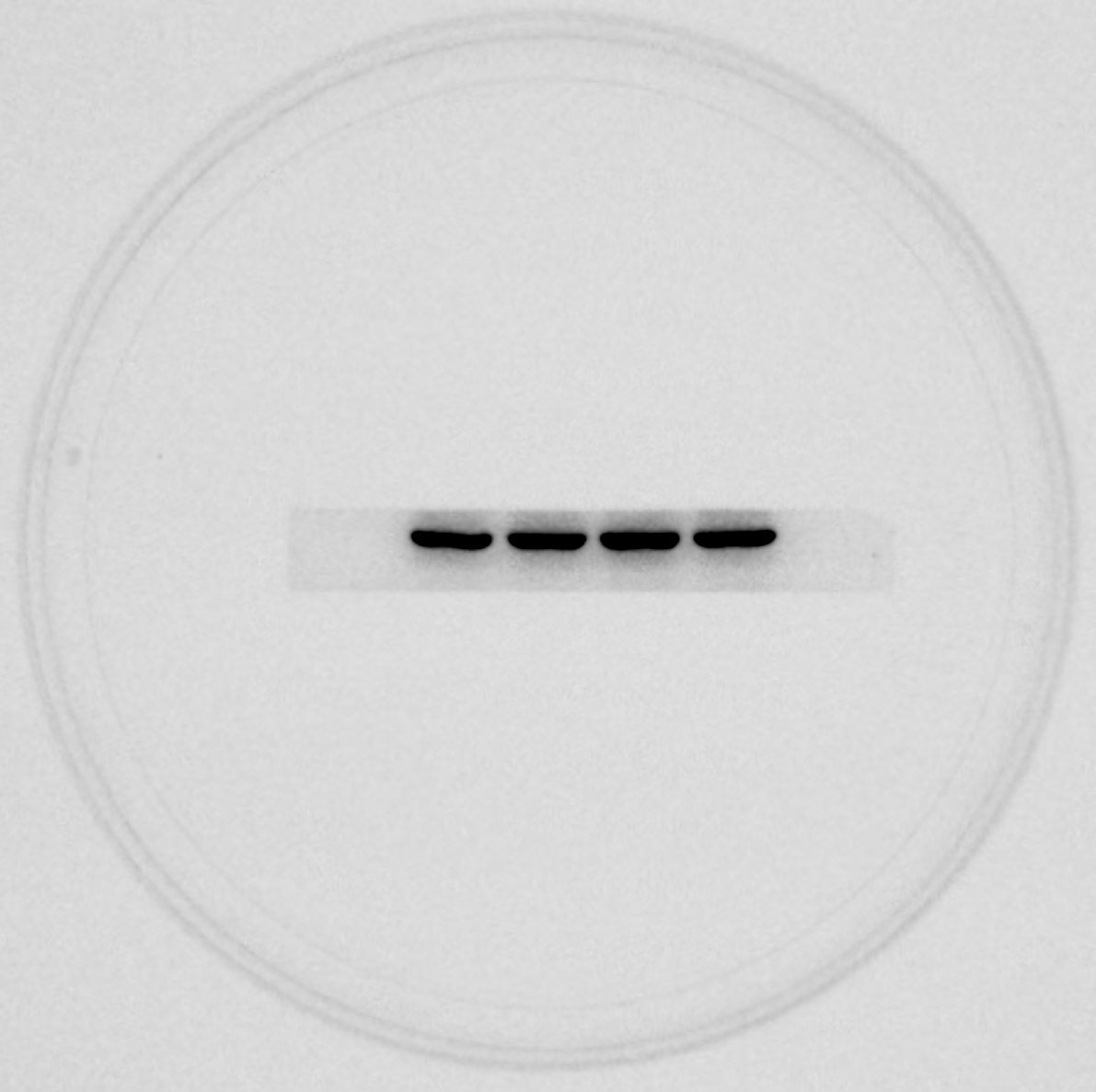

Supplement: Supplementary file 1 [file DataSheet1.ZIP › Oringinal western blots/NQO-1/a┬-actin/a┬-actin (1).tif]

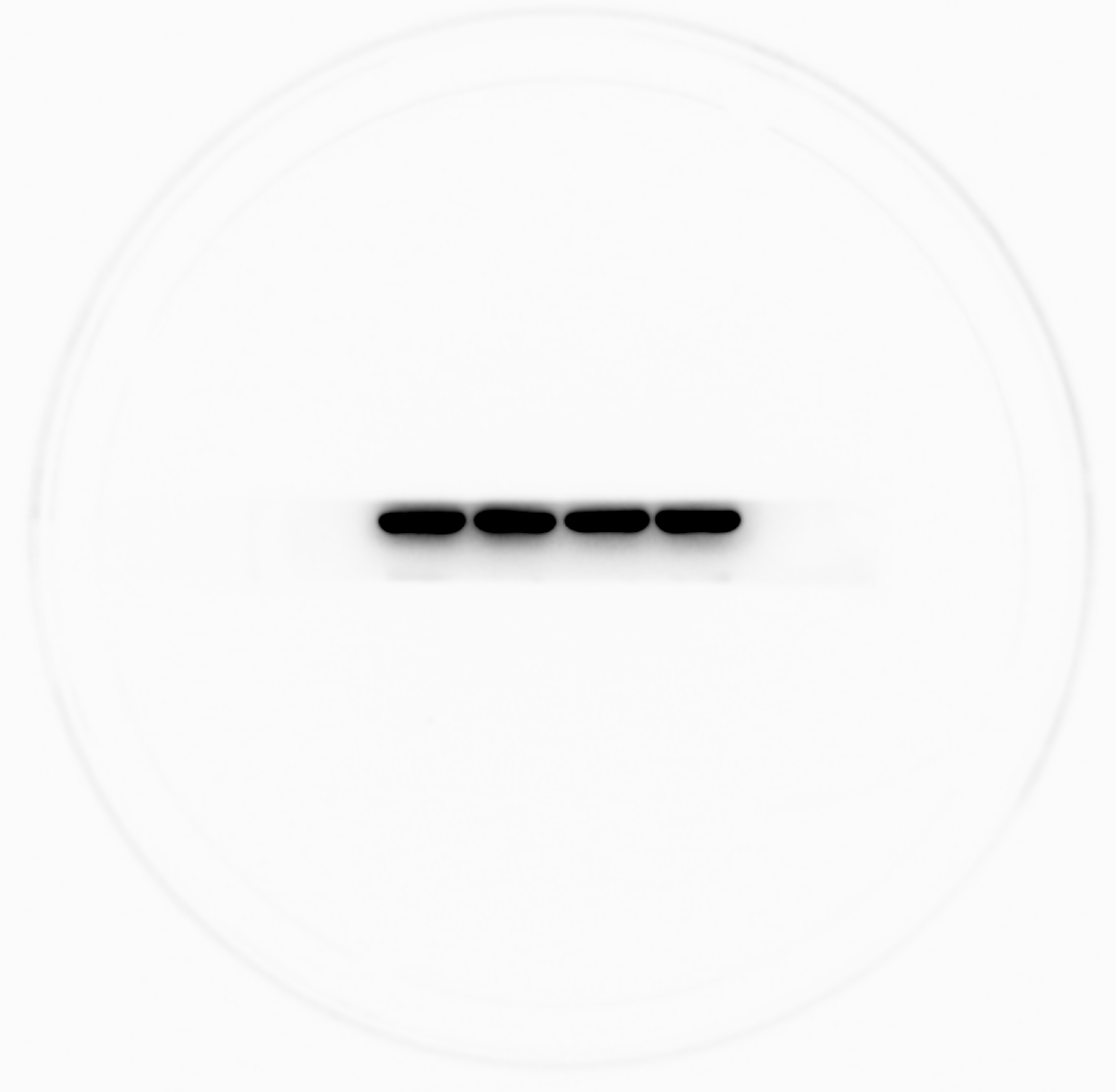

Supplement: Supplementary file 1 [file DataSheet1.ZIP › Oringinal western blots/NQO-1/a┬-actin/a┬-actin (2).tif]

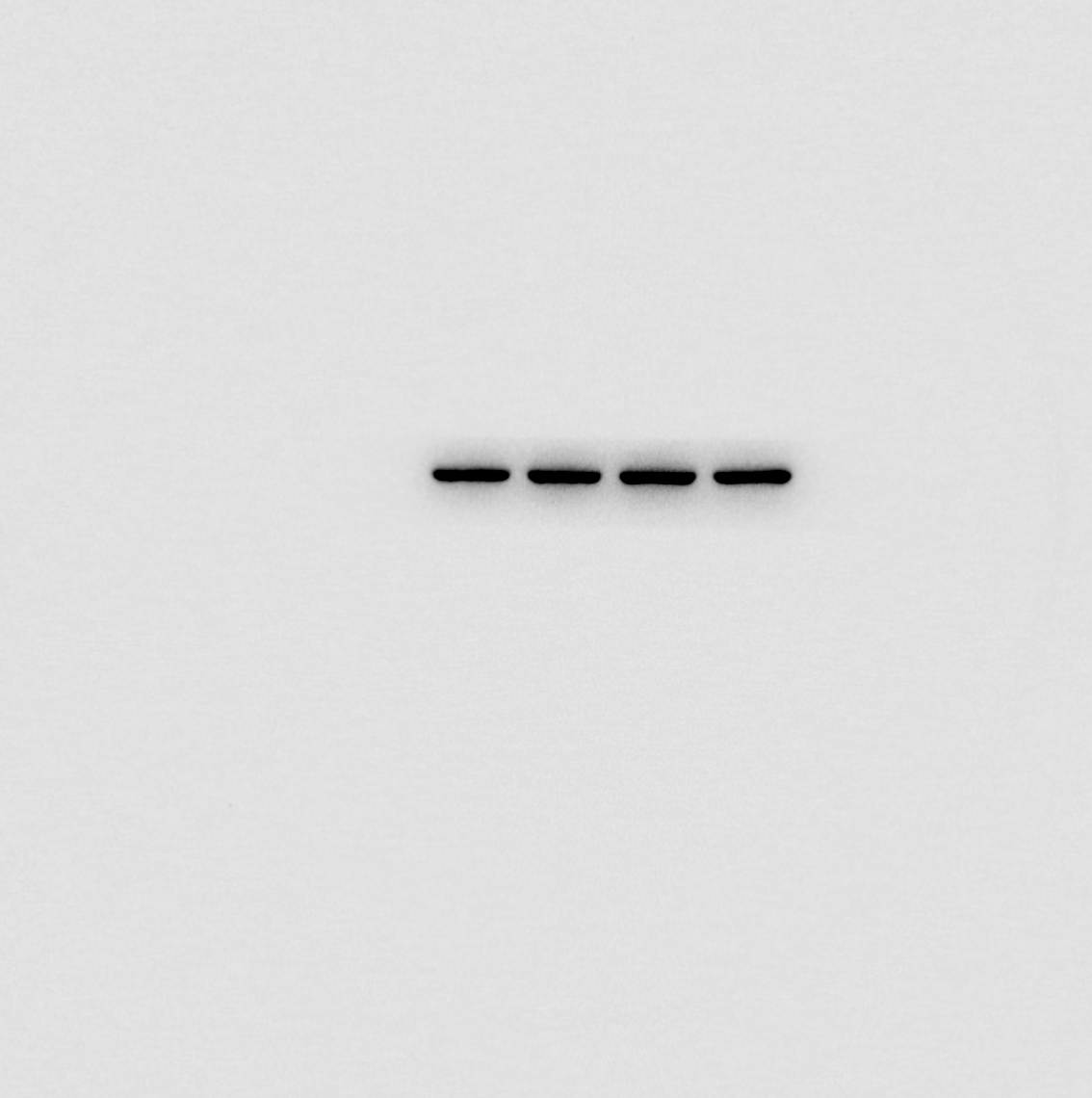

Supplement: Supplementary file 1 [file DataSheet1.ZIP › Oringinal western blots/NQO-1/a┬-actin/a┬-actin (3).tif]

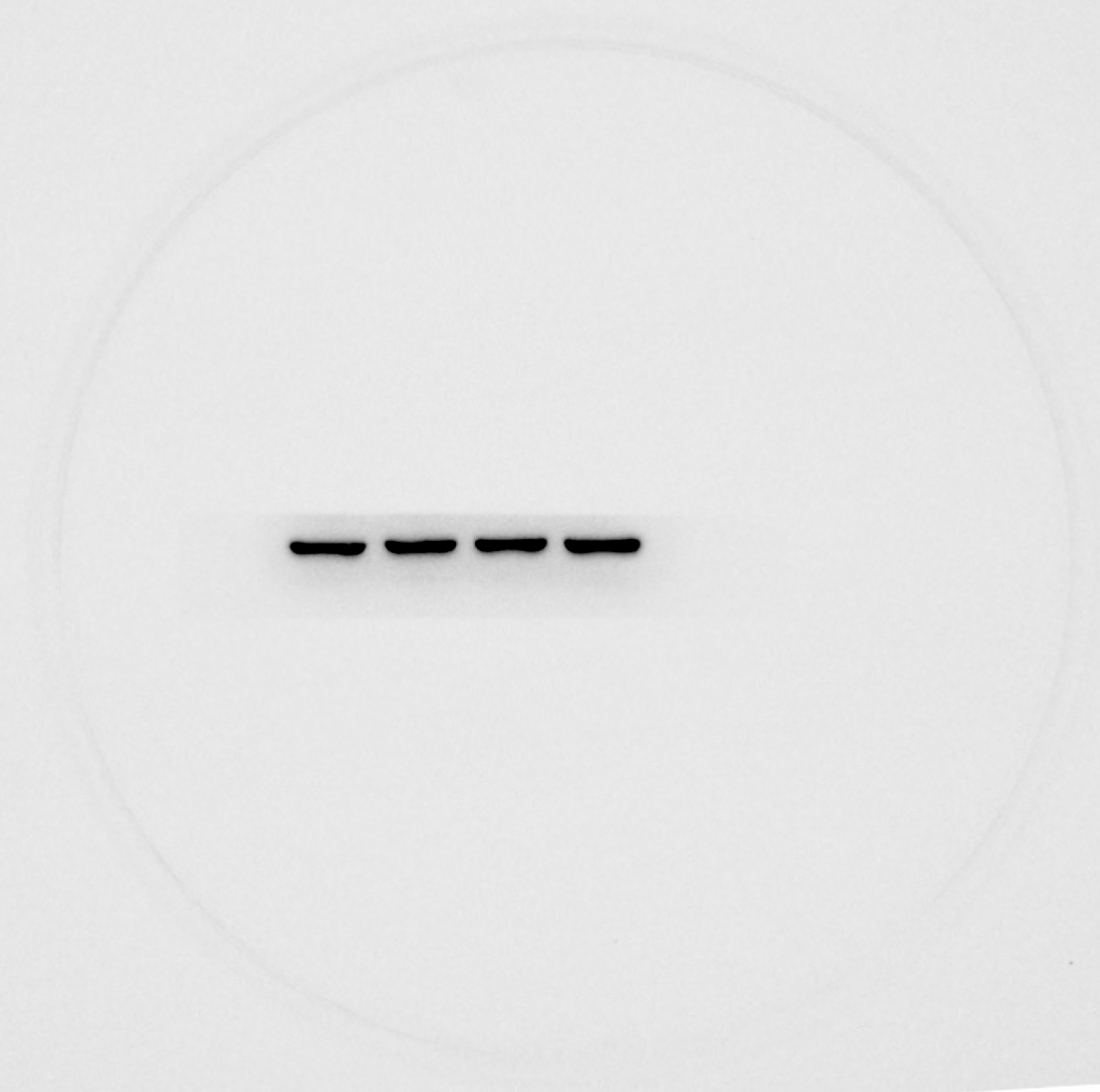

Supplement: Supplementary file 1 [file DataSheet1.ZIP › Oringinal western blots/NQO-1/a┬-actin/a┬-actin (4).tif]

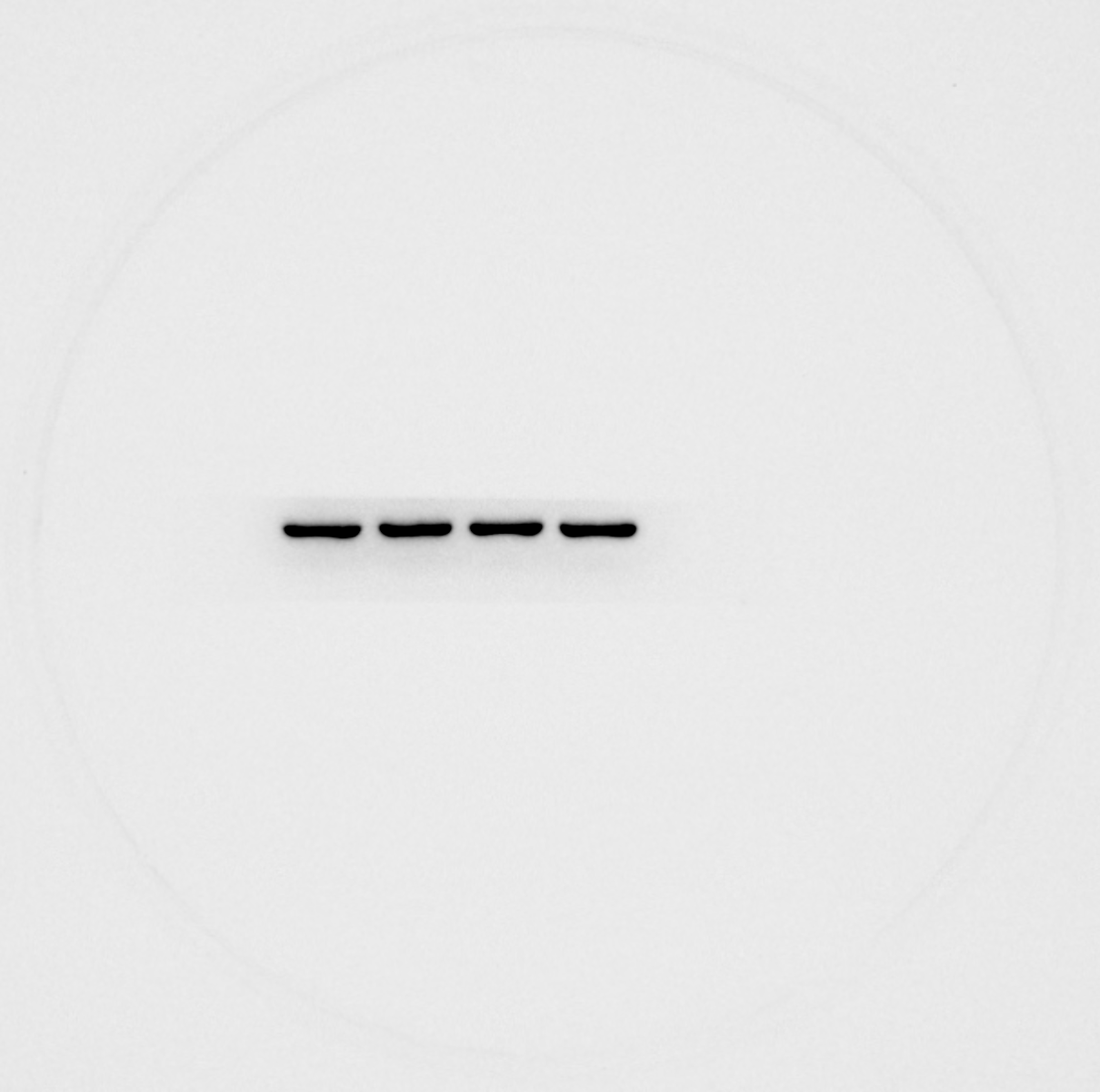

Supplement: Supplementary file 1 [file DataSheet1.ZIP › Oringinal western blots/NQO-1/a┬-actin/a┬-actin (5).tif]

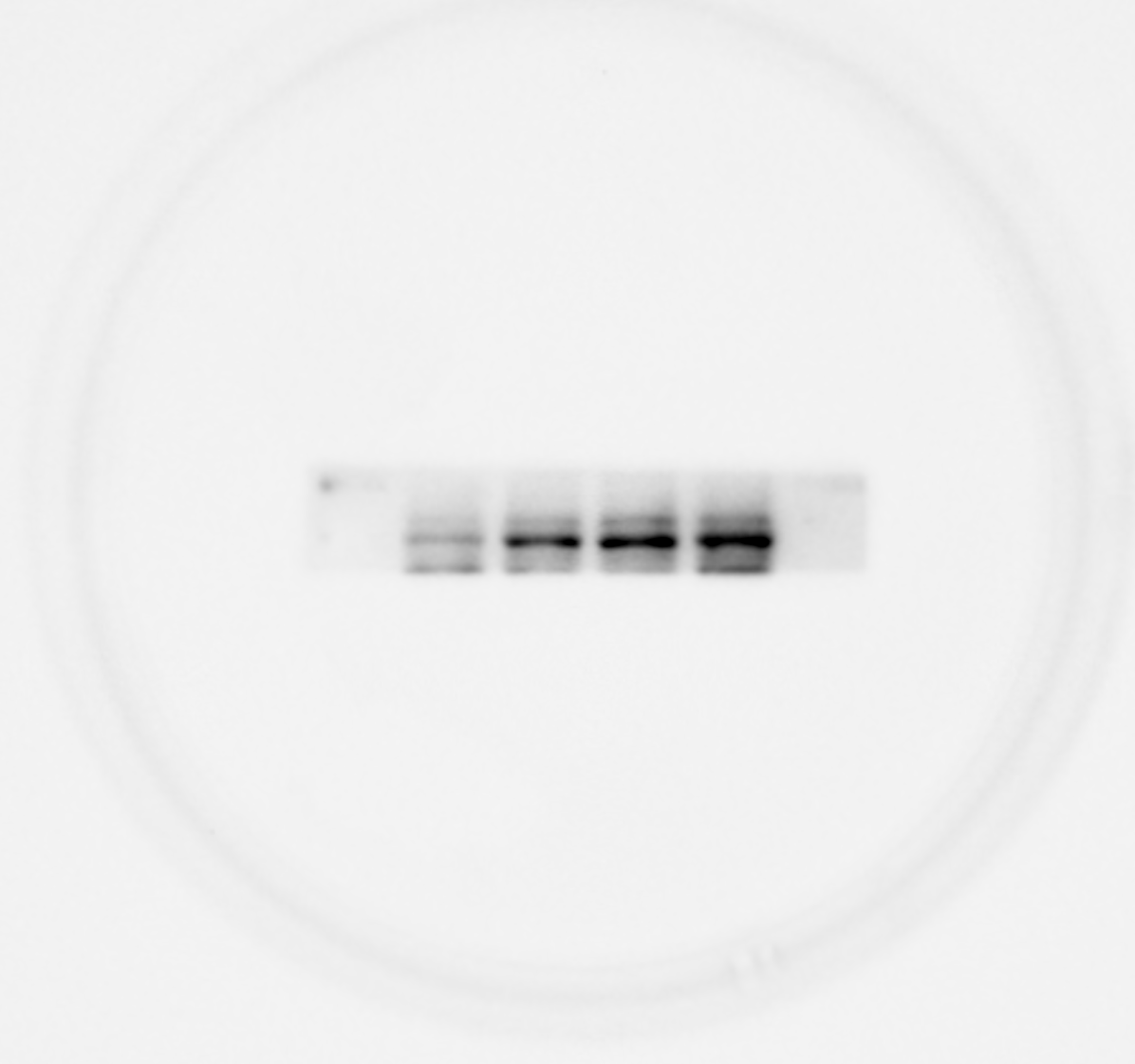

Supplement: Supplementary file 1 [file DataSheet1.ZIP › Oringinal western blots/Nucleus-Nrf2/Nucleus-Nrf2/Nucleus-Nrf2 (1).tif]

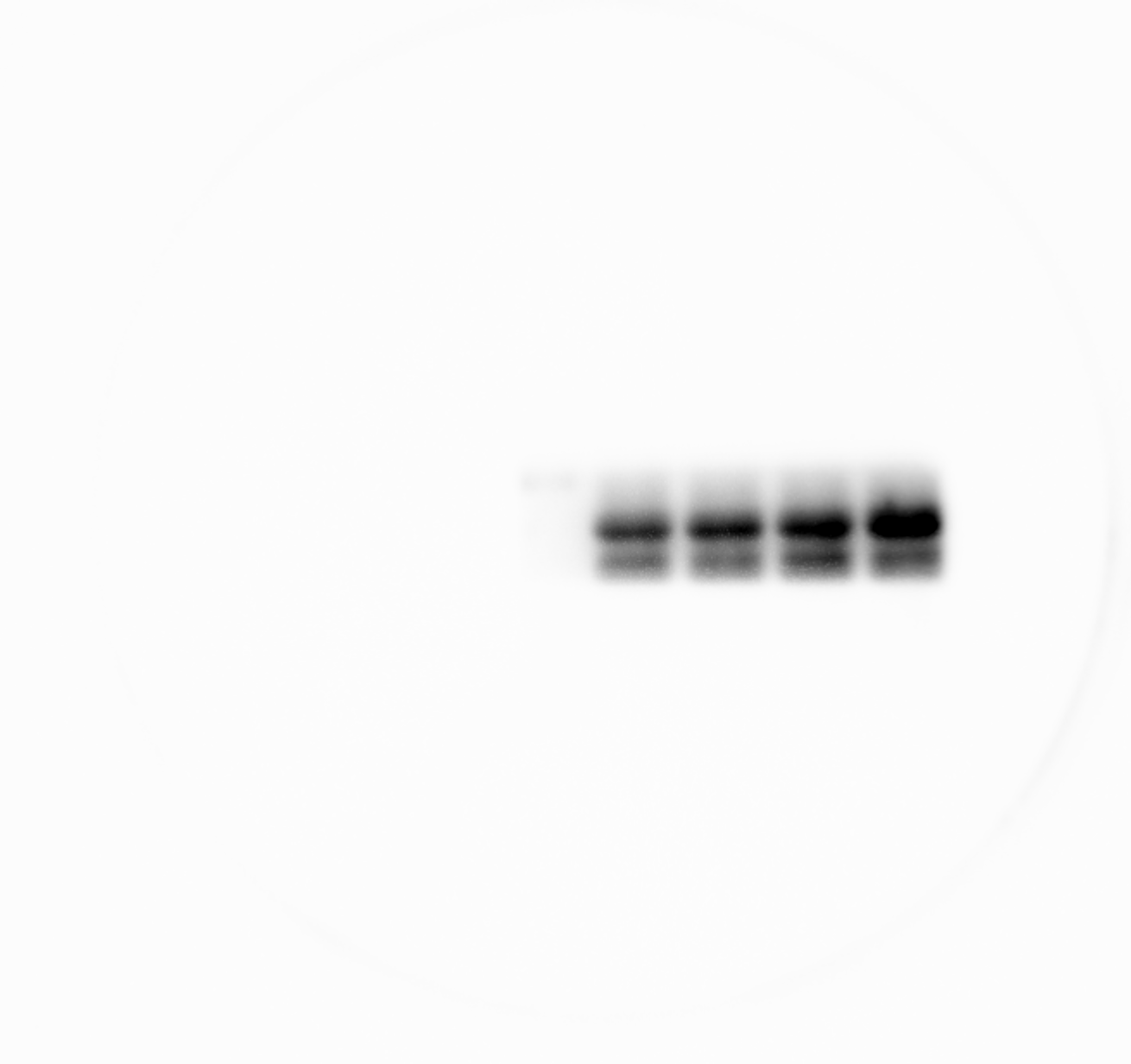

Supplement: Supplementary file 1 [file DataSheet1.ZIP › Oringinal western blots/Nucleus-Nrf2/Nucleus-Nrf2/Nucleus-Nrf2 (2).tif]

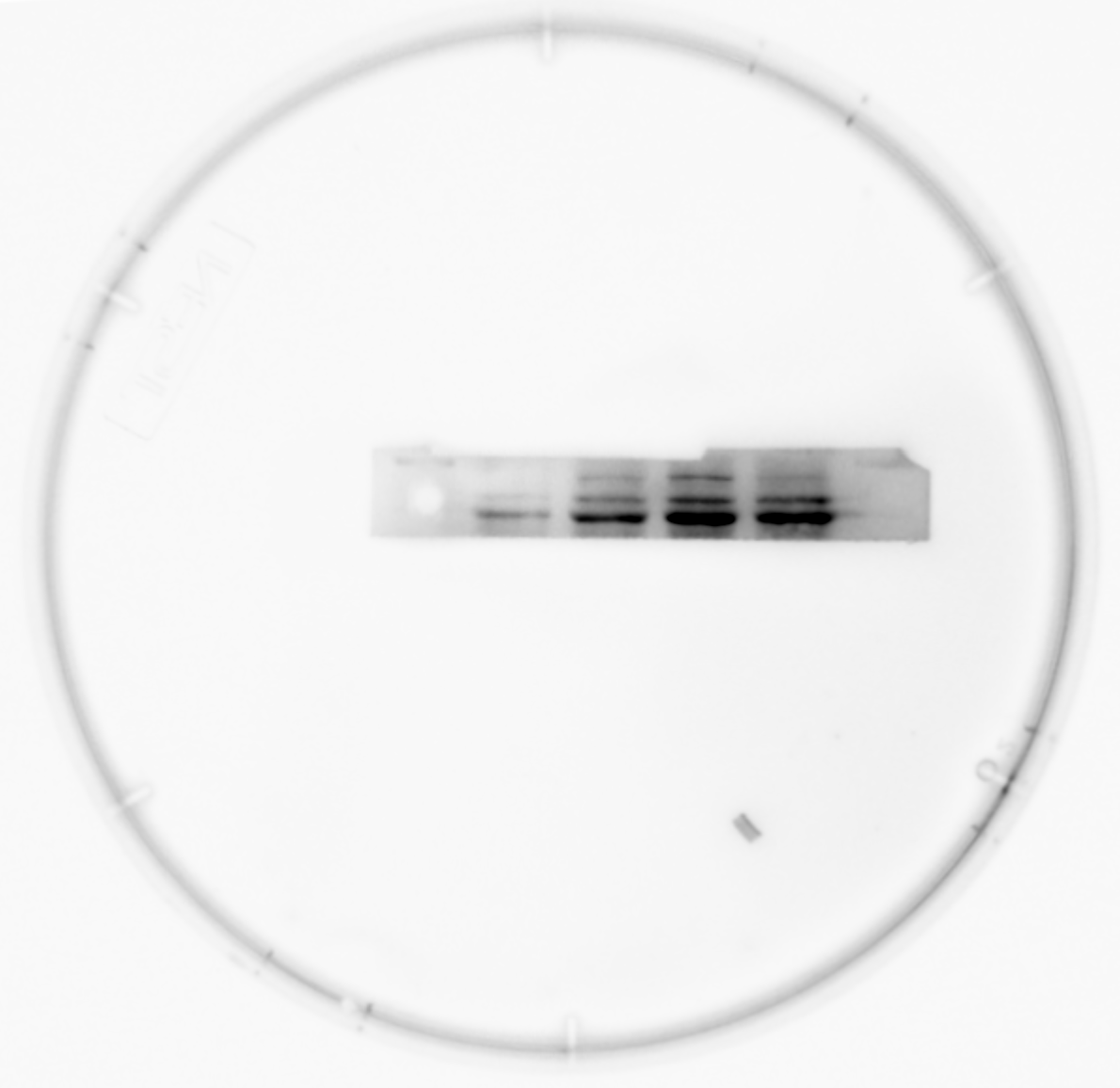

Supplement: Supplementary file 1 [file DataSheet1.ZIP › Oringinal western blots/Nucleus-Nrf2/Nucleus-Nrf2/Nucleus-Nrf2 (3).tif]

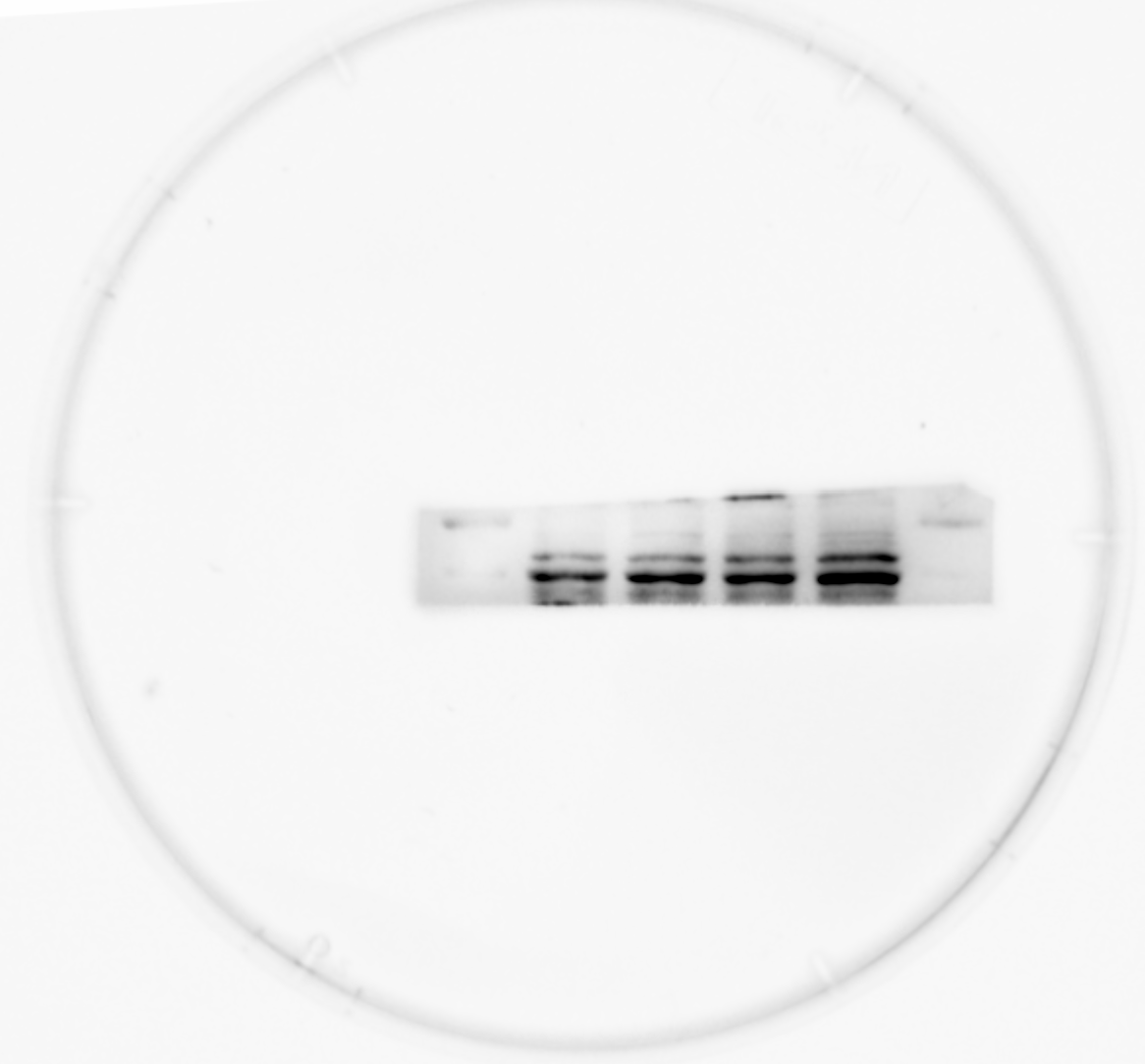

Supplement: Supplementary file 1 [file DataSheet1.ZIP › Oringinal western blots/Nucleus-Nrf2/Nucleus-Nrf2/Nucleus-Nrf2 (5).tif]

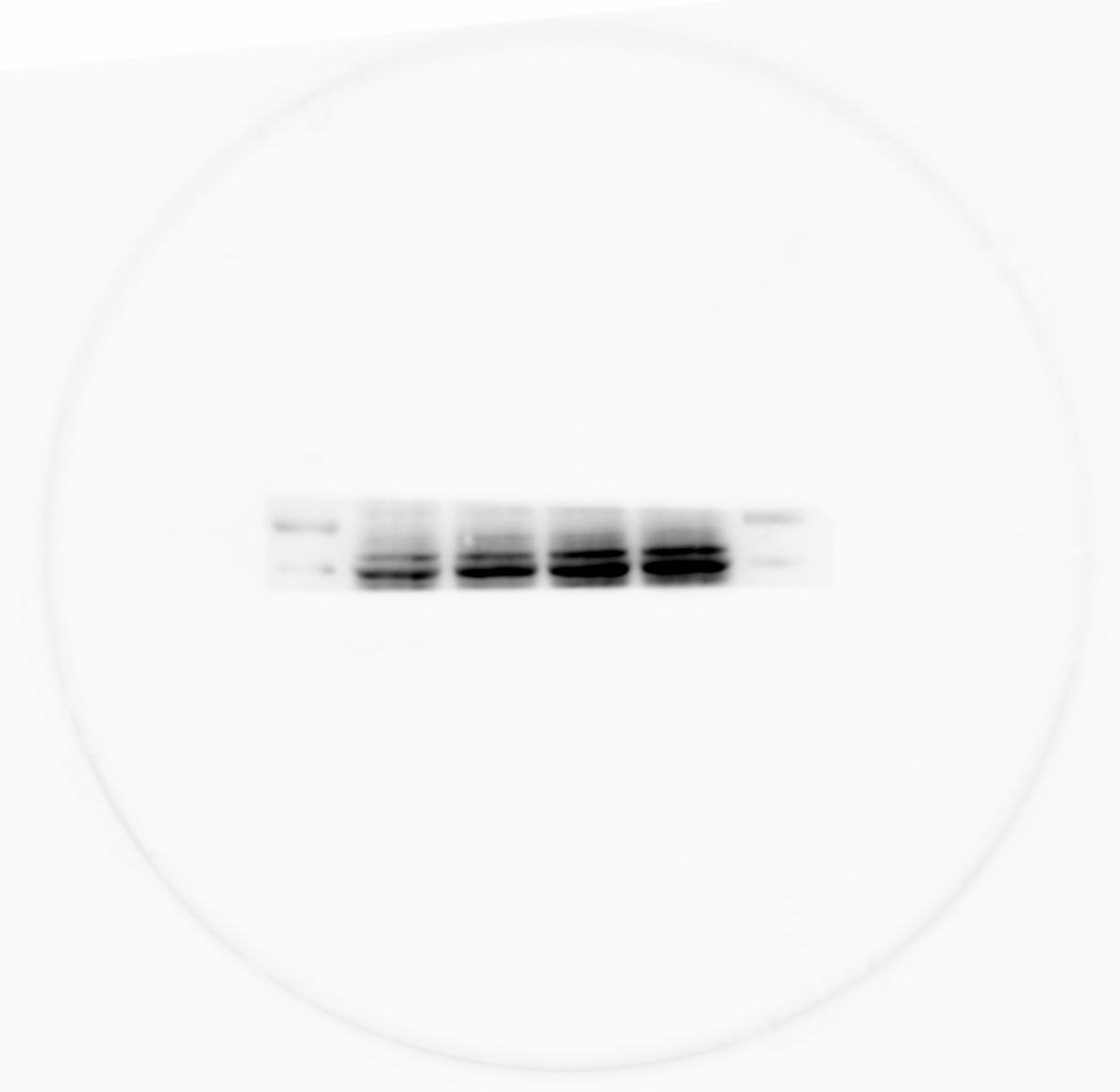

Supplement: Supplementary file 1 [file DataSheet1.ZIP › Oringinal western blots/Nucleus-Nrf2/Nucleus-Nrf2/Nuclues-Nrf2 (4).tif]

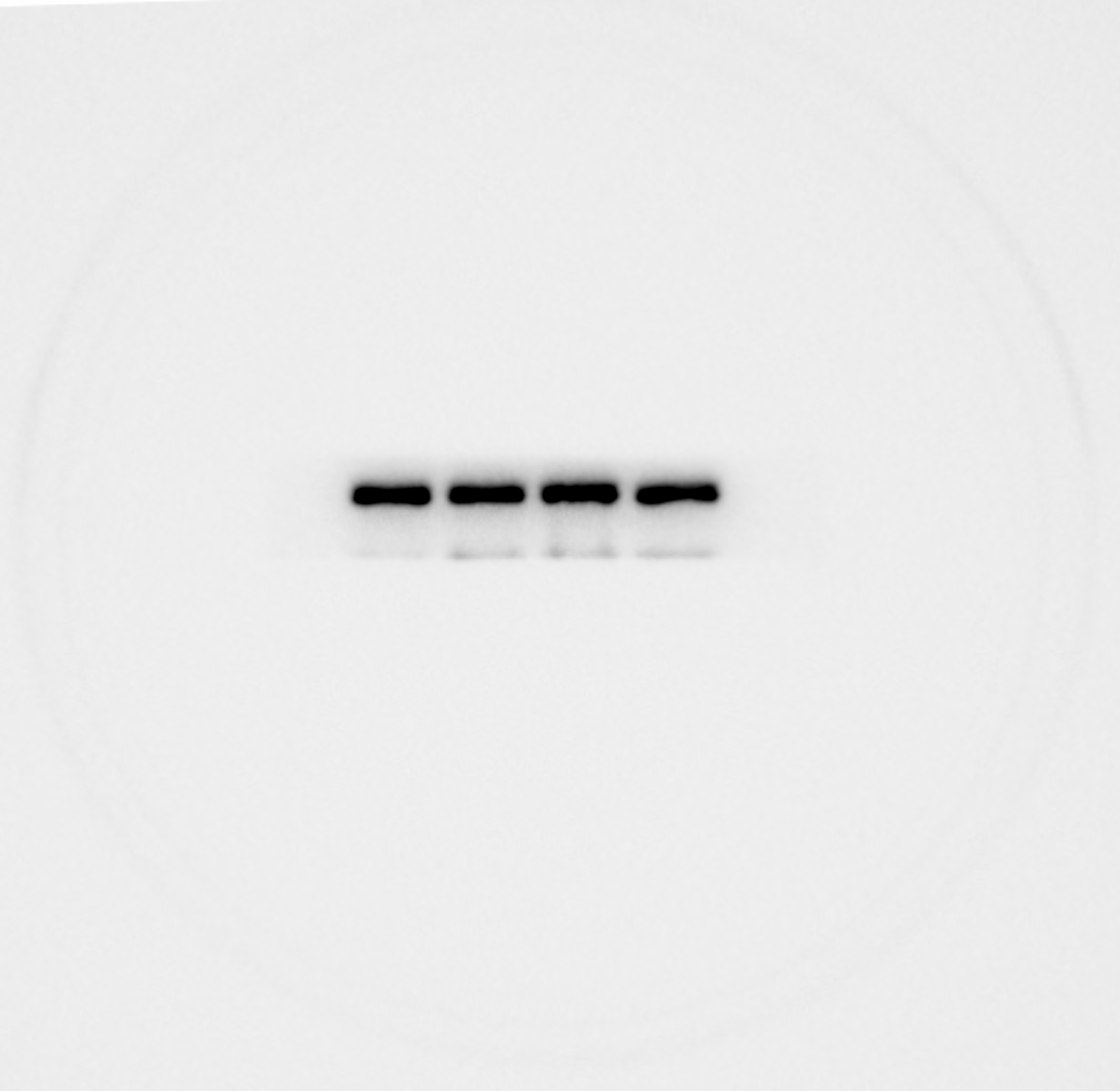

Supplement: Supplementary file 1 [file DataSheet1.ZIP › Oringinal western blots/Nucleus-Nrf2/PCNA/PCNA (1).tif]

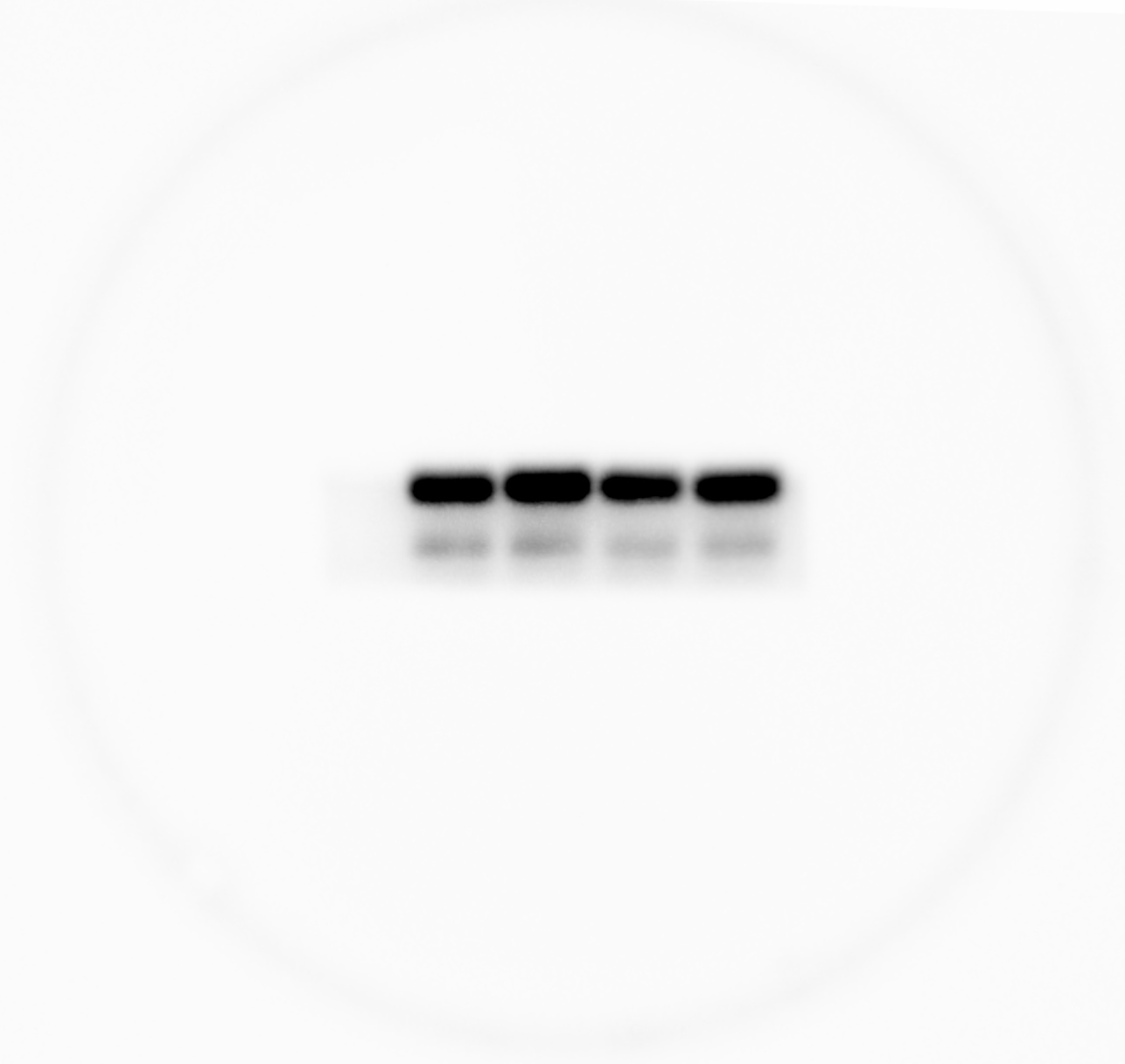

Supplement: Supplementary file 1 [file DataSheet1.ZIP › Oringinal western blots/Nucleus-Nrf2/PCNA/PCNA (2).tif]

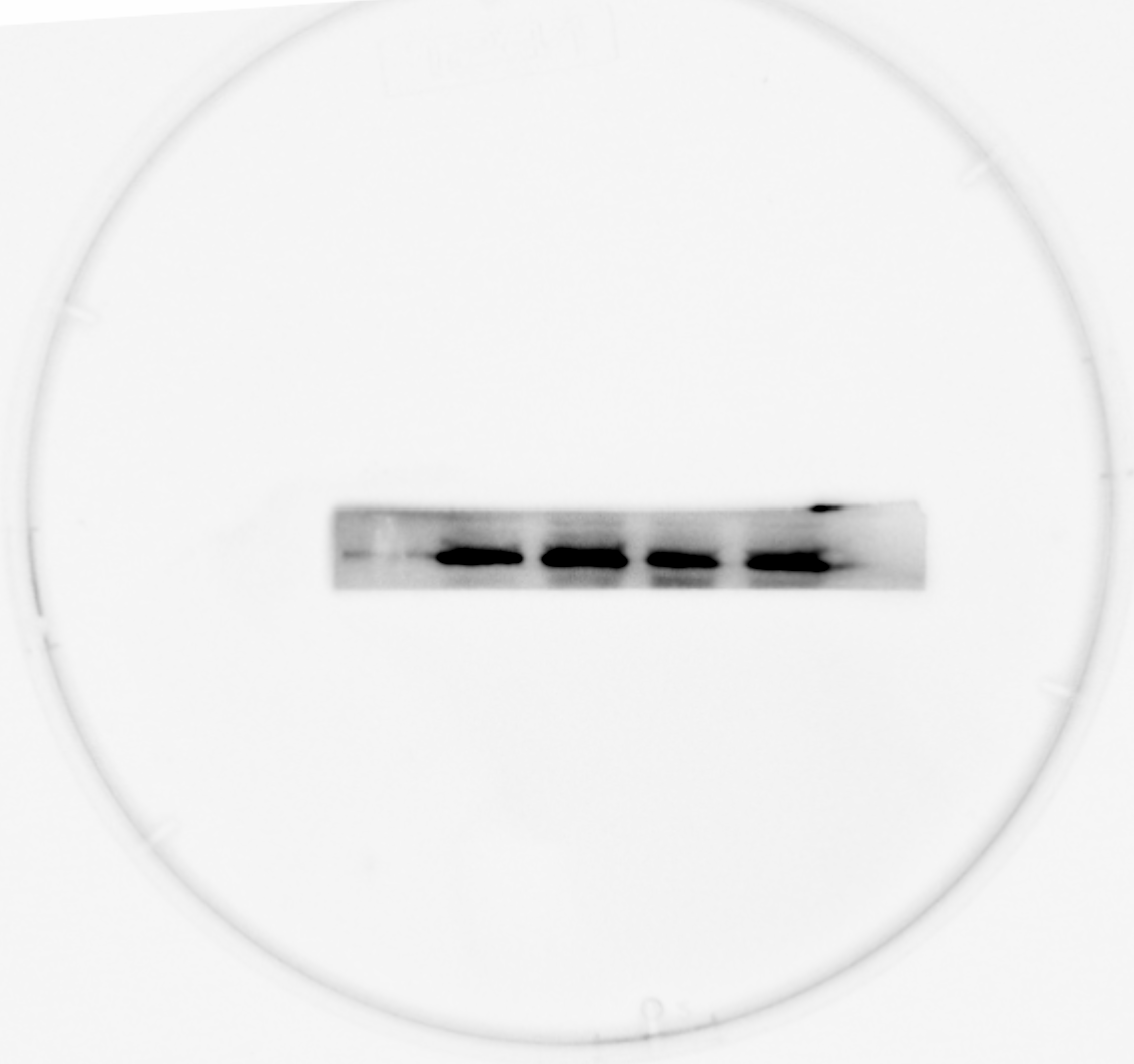

Supplement: Supplementary file 1 [file DataSheet1.ZIP › Oringinal western blots/Nucleus-Nrf2/PCNA/PCNA (3).tif]

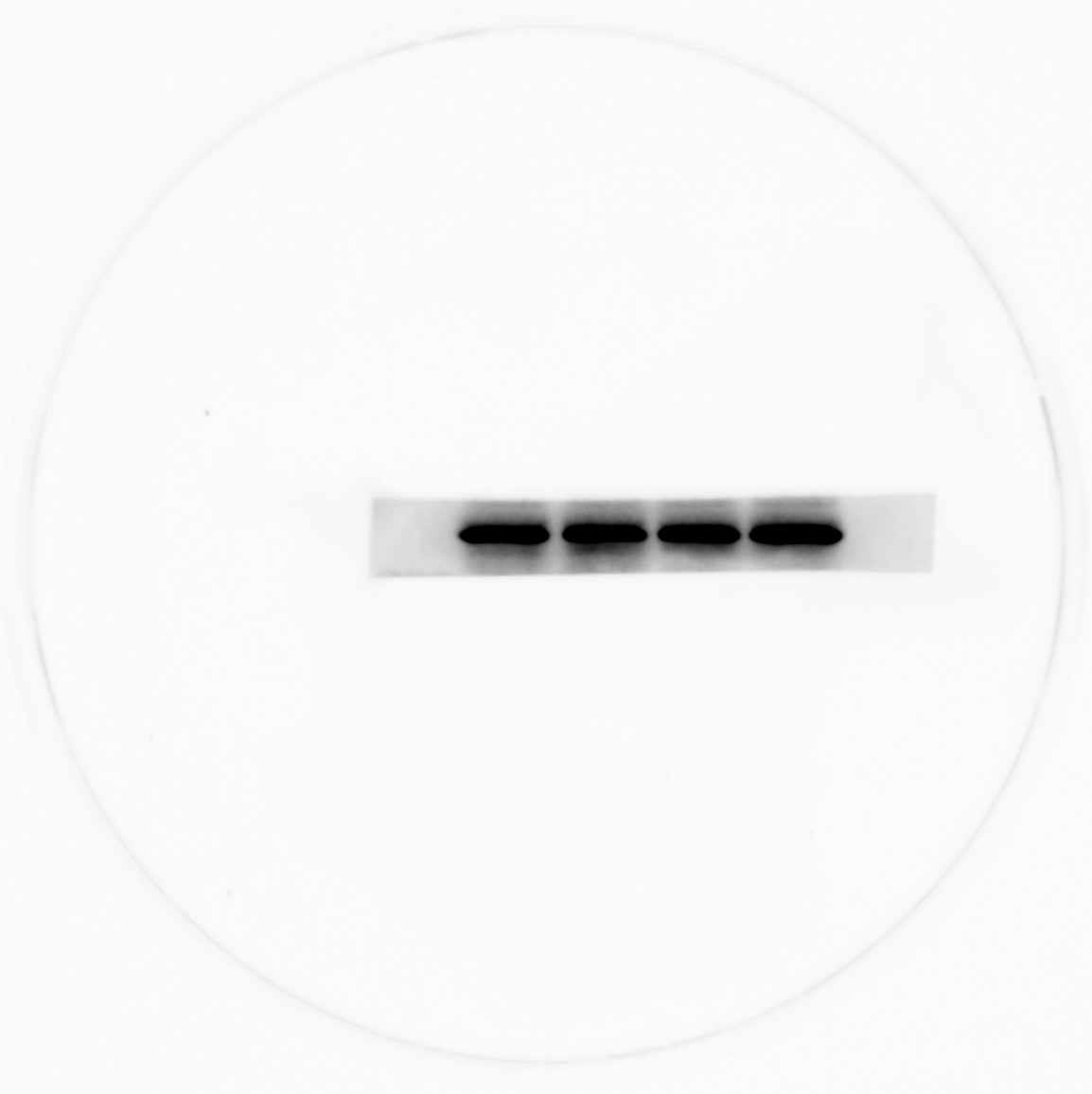

Supplement: Supplementary file 1 [file DataSheet1.ZIP › Oringinal western blots/Nucleus-Nrf2/PCNA/PCNA (4).tif]

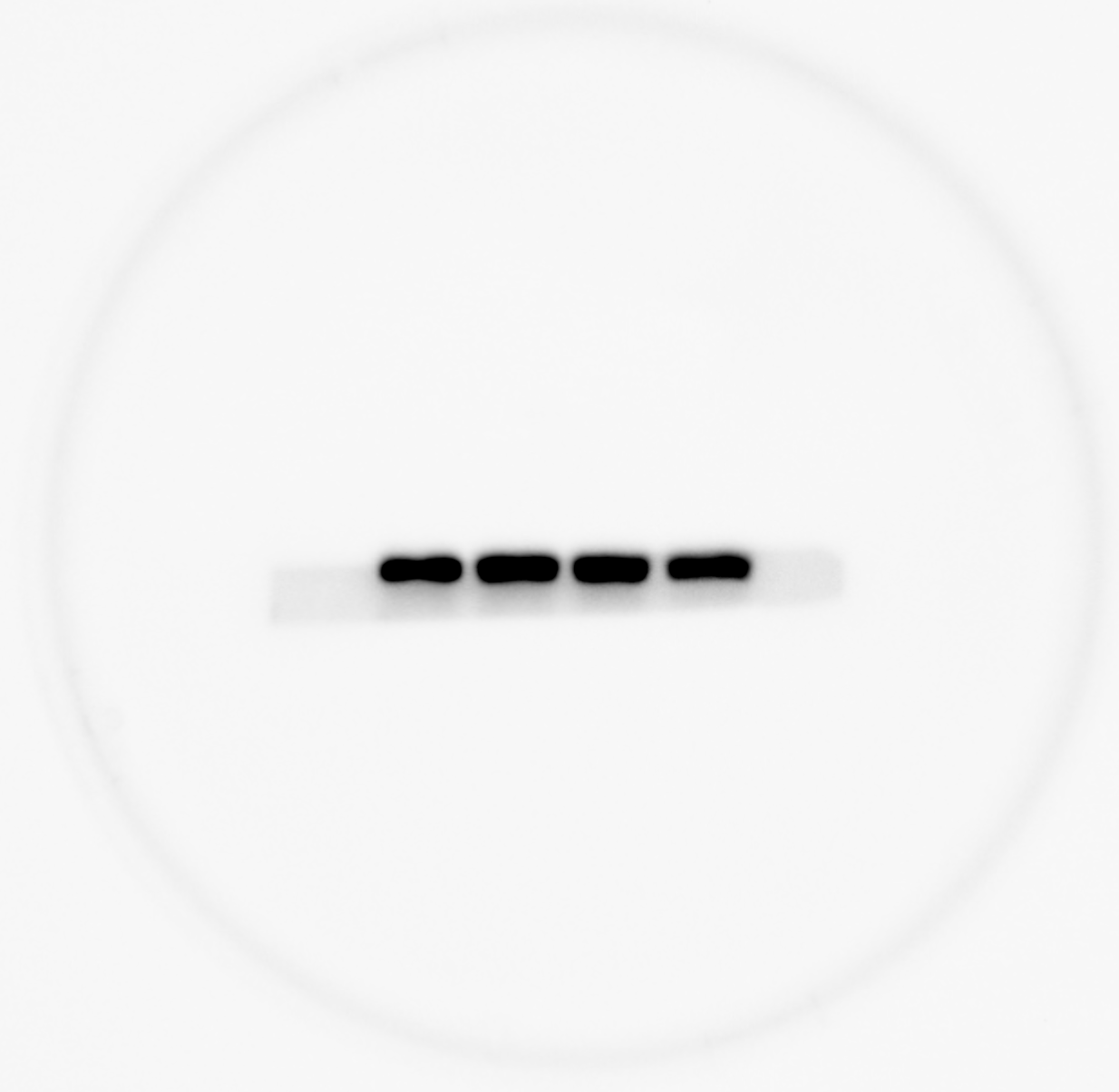

Supplement: Supplementary file 1 [file DataSheet1.ZIP › Oringinal western blots/Nucleus-Nrf2/PCNA/PCNA (5).tif]
